# Glucose Metabolism during Resting State Reveals Abnormal Brain Networks Organization in the Alzheimer’s Disease and Mild Cognitive Impairment

**DOI:** 10.1371/journal.pone.0068860

**Published:** 2013-07-23

**Authors:** Gretel Sanabria-Diaz, Eduardo Martínez-Montes, Lester Melie-Garcia, the Alzheimer’s Disease Neuroimaging Initiative

**Affiliations:** 1 Neurodiagnostic Department, Cuban Neuroscience Center, Havana, Cuba; 2 Neuroinformatics Department, Cuban Neuroscience Center, Havana, Cuba; National Yang-Ming University, Taiwan

## Abstract

This paper aims to study the abnormal patterns of brain glucose metabolism co-variations in Alzheimer disease (AD) and Mild Cognitive Impairment (MCI) patients compared to Normal healthy controls (NC) using the Alzheimer Disease Neuroimaging Initiative (ADNI) database. The local cerebral metabolic rate for glucose (CMRgl) in a set of 90 structures belonging to the AAL atlas was obtained from Fluro-Deoxyglucose Positron Emission Tomography data in resting state. It is assumed that brain regions whose CMRgl values are significantly correlated are functionally associated; therefore, when metabolism is altered in a single region, the alteration will affect the metabolism of other brain areas with which it interrelates. The glucose metabolism network (represented by the matrix of the CMRgl co-variations among all pairs of structures) was studied using the graph theory framework. The highest concurrent fluctuations in CMRgl were basically identified between homologous cortical regions in all groups. Significant differences in CMRgl co-variations in AD and MCI groups as compared to NC were found. The AD and MCI patients showed aberrant patterns in comparison to NC subjects, as detected by global and local network properties (global and local efficiency, clustering index, and others). MCI network’s attributes showed an intermediate position between NC and AD, corroborating it as a transitional stage from normal aging to Alzheimer disease. Our study is an attempt at exploring the complex association between glucose metabolism, CMRgl covariations and the attributes of the brain network organization in AD and MCI.

## Introduction

The Alzheimer Disease is the most common cause of dementia in older adults and accounts for 50%–60% of all cases. This neurodegenerative disorder is characterized by deficits in cognitive and behavioral functions, personality changes and impaired activities of daily living [1] leading to complete need for care within several years after clinical diagnosis. According to the 2011 World Alzheimer report, 36 million people worldwide are living with dementia, with numbers doubling every 20 years to 66 million by 2030 (World Alzheimer report 2011, http://www.alz.co.uk/research/worldreport). With the size of the elderly population rising and incidence of dementia also increasing AD soon could be define as a modern epidemic with an enormous economic impact.

Neurodegenerative diseases like Alzheimer are not random or confluent, especially target large-scale distributed networks. The progression of two principal neuropathological biomarkers: accumulation of b-amyloid (Ab) plaques and neurofibrillary tangles composed of tau amyloid fibrils show a spatiotemporal mechanism through vulnerable pathways connecting coactive distant brain structures rather than through neighbor regions [2]–[6]. On the other hand, it has been evidenced in transgenic mice that the AD develops in an anatomical cascade contrasting the hypothesis of individual failure events [7], [8]. In summary, these studies in animals and humans suggest that the Alzheimer Disease is a ‘*disconnection syndrome*’, where the impairment of high-level cognitive functions is associated with functional and structural disruptions between anatomically distant brain regions [9]. Hence, the brain network analysis could potentially provide new phenotypes and biomarkers of the AD pathology to be used with diagnostic and therapeutic purposes to control the impact of this disease.

Recently, brain network analysis based on graph theory has been applied to study the connectivity patterns and its underlying topological properties in AD through different neuroimaging techniques [10]–[16]. From these studies, as the most relevant issue, it was shown that AD patients exhibit abnormal segregated connectivity patterns and disruptive system integrity in large-scale brain networks (see review in [17]).

Yet, this attractive approach to characterize complex brain networks has not been practically explored using the regional cerebral metabolic rate for glucose (CMRgl) information provided by the 18F-labelled Fluro-Deoxyglucose Positron Emission Tomography (FDG-PET) technique. To date, the measurement of the CMRgl driven by basal neuronal activity is considered as an imaging biomarker with a good sensitivity in the early diagnosis of AD [18], [19]. The CMRgl reductions are thought to reflect declines in the activity or density of terminal neuronal fields or perisynaptic glials cells, mitochondrial or other metabolic dysfunctions, or a combination of these factors. Consistently, several studies have found reduced CMRgl in the entorhinal cortex, hippocampus, precuneus, posterior cingulate, parietal and temporal cortex in AD patients. However, glucose hypometabolism are found in frontal cortex and many brain regions as the illness becomes more severe [20]–[24]. This pattern of regional hypometabolism appears to be strongly associated with AD, yielding sensitivity and specificity between 84%–93% [25], [26]. Thus, FDG-PET technique provides unique information that can be used for tracking physiologically relevant AD disease processes, from the early diagnosis, to monitoring progression and evaluation of AD-modifying treatments in the clinical and preclinical stages of the disease [27], [28].

Albeit FDG-PET technique have been widely employed to explore different processes underlying the normal and pathological brain states, only a short list of papers have studied the glucose uptake co-variations between pairs of brain regions. Some of these studies [29]–[31] suggest that cerebral structures whose CMRgl values are significantly correlated are functionally associated, and that the strength of this association is proportional to the magnitude of the correlation coefficient. In particular, Metter et al. 1984 introduced the first study evaluating CMRgl co-variations patterns in AD patients using FDG-PET technique, assuming that the alteration of the metabolism in a single region will affect the metabolism of other brain areas with which it interrelates. Three years later, Horwitz et al. 1987 studied the patterns of the cerebral metabolic correlations comparing 21 Alzheimer’s disease patients and 21 healthy age-matched controls in resting state. In this case the authors computed the partial correlation coefficients between pairs of 59 anatomical structures accounting for the total CMRgl. Some relevant findings were: a) AD patients had significantly fewer consistent partial correlation coefficients compared to healthy controls and b) the number of reliable correlations between many bilaterally symmetric brains regions was reduced in the Alzheimer patients.

We have identified three others different approaches, making use of the same principle of functional association between brain areas based on correlation in CMRgl, that have also revealed abnormal processes related to default metabolic connectivity in AD subjects. The first uses the SPM (Statistical Parametric Mapping) to explore systematically connectivity based on voxel-wise interregional correlation analysis (IRCA) [32]–[34]. The second employs multivariate decomposition methods such as sICA (spatial Independent Component Analysis) and PCA (Principal Component Analysis) to capture the spatially distributed covariation of CMRgl uptake across the subjects [35]–[39]. The last of these approaches proposes a method based upon inverse covariance estimation (SICE) to identify CMRgl functional connectivity networks for a large number of anatomical regions and small sample sizes [40].

In spite of previous attempts, a general and integral study about the organization of the brain glucose metabolism co-variations when the CMRgl correlation coefficients matrix is considered to represent a metabolic network is an attractive strategy almost unexplored in the literature so far [41]. In the present paper we propose for the first time to study the topology of CMRgl networks through the graph theory framework in AD, Mild Cognitive Impairment (MCI) and healthy elder control (NC) populations. This approach will let us answer questions and shed light on issues such as: How the AD, MCI and NC functional CMRgl networks are organized in terms of global efficiency, clustering index, local efficiency? Are there differences in these primary attributes among AD, MCI and NC networks? Could MCI be considered a transitional stage showing an intermediate position between AD and NC in terms of network attributes? How much the CMRgl covariations could be modified by patterns of abnormal glucose metabolism in AD and MCI?

To carry out our study we made use of FDG-PET data coming from the Alzheimer Disease Neuroimaging Initiative (ADNI) database. ADNI is an ongoing, large, and longitudinal-designed study to develop clinical, imaging, genetic, and biochemical biomarkers for the early detection and tracking of Alzheimer’s disease [42], [43]. More than 360 papers have been published as a direct result of this international database in the first 6 years, providing valuable evidences about the anatomical, morphological and functional processes related to this disease [43]. In line with this, our study can be considered another step to integrate knowledge (based on a same database) in the quest for unraveling the complex processes of this important and urgent health problem worldwide.

## Materials and Methods

### ADNI Database

As mentioned above, data used in the preparation of this article were obtained from the Alzheimer’s Disease Neuroimaging Initiative (ADNI) database (adni.loni.ucla.edu). The ADNI was launched in 2003 by the National Institute on Aging (NIA), the National Institute of Biomedical Imaging and Bioengineering (NIBIB), the Food and Drug Administration (FDA), private pharmaceutical companies and non-profit organizations, as a $60 million, 5-year public-private partnership. The primary goal of ADNI has been to test whether serial magnetic resonance imaging (MRI), positron emission tomography (PET), other biological markers, and clinical and neuropsychological assessment can be combined to measure the progression of mild cognitive impairment (MCI) and early Alzheimer’s disease (AD). Determination of sensitive and specific markers of very early AD progression is intended to aid researchers and clinicians to develop new treatments and monitor their effectiveness, as well as lessen the time and cost of clinical trials.

The Principal Investigator of this initiative is Michael W. Weiner, MD, VA Medical Center and University of California – San Francisco. ADNI is the result of efforts of many co-investigators from a broad range of academic institutions and private corporations, and subjects have been recruited from over 50 sites across the U.S. and Canada. The initial goal of ADNI was to recruit 800 adults, ages 55 to 90, to participate in the research, approximately 200 cognitively normal older individuals to be followed for 3 years, 400 people with MCI to be followed for 3 years and 200 people with early AD to be followed for 2 years. For up-to-date information, see www.adni-info.org. Full details of subject recruitment, PET scanning protocols, and data preprocessing were published elsewhere [23], [42], [44] (http://www.loni.ucla.edu/ADNI/) and only a brief account is given here.

### Ethics Statement

Study subjects gave written informed consent at the time of enrollment for imaging and genetic sample collection and completed questionnaires approved by each participating sites Institutional Review Board (IRB). For more information, please refer to the ADNI website (http://www.adni-info.org). The authors state that they have obtained approval from the ADNI Data Sharing and Publications Committee for use of the data and confirm that the data was analyzed anonymously.

### Subjects

The data used in our study come from a subset of AD, MCI, and cognitively normal ADNI participants who had completed at least two visits at the time of this study and fulfilled other criteria explained below. For ADNI full inclusion/exclusion criteria see http://www.adni-info.org.

Subjects between the ages of 55 and 90 were enrolled in the ADNI study. Eligibility criteria were as follows (see Petersen et al., 2010, for a description of participant recruitment and classification protocol). Normal elderly controls had a Mini Mental State Examination (MMSE) score of 24 or higher [45], a Clinical Dementia Rating (CDR) of 0 [46], and no diagnosis of neurological disease or psychiatric disorder. MCI patients had a MMSE score of 24 or higher, a subjective memory complaint, objective memory loss measured by education adjusted scores on the Wechsler Memory Scale Logical Memory II, a CDR score of 0.5, absence of significant levels of impairment in other cognitive domains, preserved activities of daily living (ADLs), and an absence of dementia [47]. Participants with mild AD were enrolled if they had a MMSE score between 20 and 26 (inclusive), a CDR score of 0.5 or 1.0, and met NINCDS-ADRDA criteria for probable AD [48].

At the initiation of our study, 489 ADNI participants with both baseline FDG-PET and MRI data were available for downloading from the ADNI LONI (University of California, Los Angeles) website (http://www.loni.ucla.edu/ADNI/) to be included in this study.

Finally we used imaging data from 199 participants (69 AD, 62 MCI, 68 NC), whose group wise characteristics are provided in Table 1. These subjects met the following criteria: anatomical study acquired in a 1.5 Tesla MRI machine, right handedness, the period between baseline MRI and PET-FDG acquisitions was less than 2 months, good image quality for MRI and PET acquisitions, and the neuropsychological variables were in accordance with eligibility criteria that characterize the AD, MCI and NC groups defined above.

**Table 1:** Demographic and Neuropsychological Data.

Parameter	AD Group (n = 69)	MCI Group (n = 62)	NC Group (n = 68)	p-value
Subject age+	75.83±7.35	76.49±7.21	76±5.06	0.84
No. of male subjects*	41 (60)	36 (58)	43 (63)	–
Years of education+	14.48±3.05	16.19±3.11	15.47±3.20	0.007^a^
MMSE score+	23.68±2.04	27.02±1.88	29.07±1.14	<0.001a,b,c
CDR score 0	0	0	68	–
CDR score 0.5	29	62	0	–
CDR score 1	40	0	0	–

Note. – MMSE = Mini-Mental, State Examination, CDR = clinical dementia rating scale.

* Data are numbers of subjects, with percentages in parentheses.

+ Mean value, with standard deviation.

Baseline demographic differences between NC, MCI, and AD participants were analyzed using one-way analysis of variance (ANOVA), Fisher’s exact and Chi-square (χ2) tests. Scheffé-multiple comparison test was used to compare the differences between each pair of means.

a AD significantly different from MCI.

b AD significantly different from NC.

c MCI significantly different from NC.

### ADNI FDG-PET Acquisition

The FDG-PET images had been acquired using Siemens, GE and Philips PET scanners according to one of three standard protocols (30–60 minute dynamic, 30–60 minute static, 0–60 minute dynamic) following the intravenous injection of 185±19 MBq of FDG. Data were corrected for both scatter and measured attenuation, which was determined using the CT scan for PET/CT scanners, and a transmissions scan with ^68^Ge or ^137^Cs rotating rod sources for PET-only scanners. Images were reconstructed using scanner-specific algorithms, and sent to the University of Michigan, where they were reviewed for artifacts, de-identified, and transmitted to the Laboratory of Neuroimaging (LONI) for storage. Further details are available in the ADNI PET technical procedures manual (ADNI PET Core) [44].

### FDG-PET Image Pre-processing

The 199 FDG-PET scans matching to the first MRI acquisition were downloaded from LONI Image Data Archive in Nifti format. Each image was examined for major artifacts, and its orientation adjusted if necessary. The 30–60 minute dynamic scans were corrected for patient motion using SPM5 (http://www.fil.ion.ucl.ac.uk/spm/software/spm5) to register each of the subsequent frames rigidly to the image’s first frame. The resulting co-registered frames were averaged to produce a single 30–60 minute static image. For the 0–60 minute dynamic scans, the final six 5-minute frames were extracted, and concatenated into a static image in the same way. This procedure has been used previously by other authors based on ADNI data [49]–[52].

### ADNI MRI Acquisition and Pre-processing

Pre-processed versions of the 199 baseline T1-weighted 1.5 T MRI scans were downloaded from the LONI Image Data Archive in Nifti format. These had been acquired using Siemens, GE and Philips MRI scanners, according to a standard protocol involving two scans per subject that were based on a 3-D MPRAGE imaging sequence. Further details are available in the ADNI MRI technical procedures manual (ADNI MRICore, 2005). From the two images acquired per subject, the ADNI quality assurance team selected the best image for pre-processing, based on the presence and severity of common image artifacts, as well as other criteria. Pre-processing involved the application of a scanner-specific correction for gradient non-linearity distortion (Gradwarp; [53]), followed by a correction for image intensity non-uniformity (B1; [54]), and finally a histogram peak sharpening algorithm for bias field correction (N3; [55]). Only the N3 pre-processing step was necessary for images acquired on Philips scanners, since B1 correction was already implemented, and their gradient systems tended to be linear [54].

### Co-registration between FDG-PET and Structural MRI

For each subject, the pre-processed FDG-PET image was co-registered with the corresponding pre-processed MRI image by means of the between modality coregistration methodology using information theory, and finally re-sampled to the higher resolution of the MRI. The Normalized Mutual Information cost function was employed to estimate a 12-parameter (degree of freedom) affine transformation matrix to transform voxels from PET to MRI space. SPM5 tools (http://www.fil.ion.ucl.ac.uk/spm5/) were used to perform non-linear registration.

### MRI and PET Parcellation. Assessing Brain Structures Volumes Based on the High Resolution MRI Imaging. Construction of the CMRgl Data Matrix using the FDG-PET Imaging

Using IBASPM toolbox (Individual Brain Atlases using the Statistical Parametric Mapping (SPM) available at http://www.fil.ion.ucl.ac.uk/spm/ext/#IBASPM) [56], the gray matter tissue of the T1- weighted and FDG-PET images were automatically segmented into 90 anatomical structures using the AAL atlas described in Tzourio-Mazoyer et al. (2002) (the full list of the structures can be found in Table S1). Volumes of the 90 structures were computed from these segmented T1 images to be used as a covariate to subtract partial volume effects of the matched mean glucose metabolism images. From the segmented FDG-PET images the mean glucose metabolism for each AAL’s structure were extracted to obtain the CMRgl data matrix. CMRgl data matrix is M x N, where ‘M’ rows represent the number of subjects, and ‘N’ the number of AAL structures. In addition, PET images were also segmented in 71 structures using the Jacob Atlas developed at the Montreal Neurological Institute (MNI, http://www.mni.mcgill.ca/) that includes the brainstem. Mean glucose metabolism of the brainstem was used for FDG-PET normalization purposes. The multiregion FDG-PET data using AAL atlas (or equivalent resolution) has been previously used without finding any relevant effect on the results [40], [49], [52]. However a possible non-independence of regions should always be taken into account when interpreting the results.

In short, the IBASPM methodology consists of two main steps. Firstly, the MR image is normalized to MNI (Montreal Neurological Institute) space using a nonlinear normalization to obtain the spatial transformation matrix. Additionally, in this step the individual images are segmented in three different brain tissues (cerebral spinal fluid, gray matter and white matter). Secondly, each individual gray matter voxel is labeled based on an MNI anatomical atlas (constructed by manual segmentation for a group of subjects) and the transformation matrix obtained in the previous step. The volume of each structure was obtained as the number of voxels belonging to each structure multiplied by the voxel’s volume. A flowchart of the IBASPM pipeline can be found at the web page: http://www.thomaskoenig.ch/Lester/ibaspm.htm. The IBASPM procedure has been used by previous authors (cited 67 times so far) for studying volume and surface area morphometric descriptors in normal and pathological brains (for a paper which has been recently published studying AD see [57]).

### FDG-PET Normalization

FDG-PET image normalization is often performed relative to the cerebral global mean. However, due to the nature of the disease process, both MCI and AD patients have a lower glucose metabolic rate than normal subjects across the whole brain. Normalization to the cerebral global mean therefore artificially scales up values from patients, whilst scaling down those from normal subjects, resulting in under-estimation of the relative hypometabolism in patients compared to normal subjects [58]. Recent work suggests that improved group discrimination can be achieved by using the signal intensity in the cerebellum, brainstem, basal ganglia, and sensorimotor cortex [26], relatively preserved regions of the brain for normalization, rather than the cerebral global mean value [59], [60]. Our analysis makes use of this normalization method using the brainstem as ‘reference cluster’ [61], [62], obtained from IBASPM segmentation of the PET-FDG images using Jacob atlas (see previous section).

### CMRgl Connectivity Matrix Construction

Prior to the CMRgl correlation analysis, a linear regression was performed at every region to remove the effects of age, gender, age–gender interaction, total CMRgl (sum of CMRgl of all anatomical structures belonging to the AAL parcellation), structure’s volume and education. The structure’s volume was introduced as a covariate in the linear regression in order to reduce the partial volume effects present in PET images. It was not found significant effect of the education variable on any regional CMRgl (p>0.05) despite the differences between AD and MCI groups reported in Table 1. Therefore this variable was not included in the final linear regression model to remove confound effects. The residuals of this regression then replaced the raw values of the CMRgl data matrix.

We defined a connection as statistical associations in glucose metabolism between each pair of brain regions for a parcellation scheme of 90 anatomical structures [63], [64]. The statistical similarity or synchronized co-variations in glucose metabolism between two regions was measured by computing the Pearson’s correlation coefficient, across subjects. Hence, the interregional Pearson’s correlation matrix (N×N, N is the number of brain regions, here N = 90) of such connections or ‘*CMRgl connectivity matrix’* was obtained using all pairs of anatomical structures. The element C*_ij_* is the value of the Pearson’s correlation between regions *i* and *j*. Self-connections were excluded, implying zeros in the diagonal of the symmetric matrix.

It is important to point out that the partial correlation analysis could not be used in our case because the sample size was not large enough (the number of structures in the AAL parcellation is higher than the number of subjects for each group) for a robust estimation of this measure.

Similar to our preceding works [63], [65], we obtained bootstrap samples of the connectivity matrix by selecting a random subset of the total number of subjects with replacement (from sample to sample) to compute the Pearson’s correlation coefficient. Through this procedure, it was possible to study the significance of changes in network properties between experimental groups (NC, MCI and AD) and taking into account the variability of having different combinations of subjects in the sample. In particular, we acquired Nboot = 300 bootstrap samples using a subset that contains always 80% of the total number of subjects in each group.

All connectivity matrices obtained from the 300 bootstraps were thresholded to create sparse binary graphs. Rather than restricting our analysis to a binarized graph obtained by applying a single threshold value, we explored the properties of the graphs over a range of thresholds to explore metrics with different sparseness [63], [66], [67]. The threshold values R*_k_* (different for each of the 300 connectivity matrices) were calculated to obtain different matrix sparsity that we denote as ‘sparsity degree’. A sparsity degree of 0.9 means that 90% of the connectivity matrix is discarded, therefore only the highest 10% of the connectivity values are taken into account. R*_k_* were computed for sparsity degrees ranging from 0.5 to 0.9, in steps of 0.02, yielding a set of 21 values. This procedure normalizes the networks to have the same number of nodes and edges, enabling the examination of the relative network properties obtained for each group. The range of sparsity degree was chosen to allow for all network properties to be properly estimated and the number of spurious edges in each network minimized as indicated in previous studies [68], [69].

In these matrices, an element was set to 1 if the absolute value of the glucose uptake correlation between two regions ‘*i*’ and ‘*j*’ C*_ij_* was higher than R*_k_*; (| C*_ij_* |> R*_k_*) and 0 otherwise. This binarized connectivity matrix captures the glucose metabolism covariations patterns of the population samples under study.

### Graph Analysis to Characterize CMRgl Network

A great number of natural systems can be represented by complex networks. Graph Theory is usually considered an attractive model for the mathematical treatment of such networks, including those representing brain connectivity [70]. In general, a complex network can be represented as a graph G = [N,K], the components of this system are called nodes (N) and the relations or connections between them are called edges (K) [71]. In our specific case, nodes represent the anatomical structures obtained through IBASPM automatic brain parcellation procedure, whereas edges denote the co-variations in CMRgl between pairs of these brain regions.

It is important to note here that this is a mathematically derived network, whose connections do not necessarily constitute brain functional or physiological mechanisms directly. However, these networks are based on functional data and therefore they indirectly reflect the underlying mechanisms, allowing us at the same time to use them and their properties as possible biomarkers of the differences between normal and pathological brain states.

Thus, we are dealing with what will be called a brain CMRgl network, where each node can be assumed to exchange information directly or indirectly with other part of the network through the synchronized fluctuations of the glucose uptakes. Every network can be represented by a graph with an adjacency or connectivity matrix. The entries of this matrix represent the relationship or interactions between pairs of nodes. Therefore, we have considered the CMRgl covariations matrix as the CMRgl connectivity matrix. The CMRgl network is unweighted because all the edges are assumed to indicate relations of equivalent strength between nodes, and undirected, simply summarizing symmetric relations (such as correlations) between nodes.

We used graph theory to compare the glucose metabolism co-variation networks of NC, MCI and AD groups. This mathematical treatment allows us to characterize the disease-related changes of the global and local phenomena observed when CMRgl perturbations in any structure occurs concurrently with glucose uptake fluctuations in its neighborhoods and other distant brain structures of the network. In other words, graph theory gives us the framework to explore the CMRgl network architecture and how efficiently the information of CMRgl fluctuations is ‘exchanged’ over the network (in terms of the graph theory). Another important point is that these networks cannot be interpreted in terms of temporal causality. Firstly, because CMRgl covariations are assessed across subjects and second since we are using Pearson correlation due to data availability, thus the direct influences between pair of nodes cannot be observed remaining the influence of all other regions.

In particular, we analyzed the following global network attributes: cluster index, local and global efficiency, characteristic path length and sigma (small world attribute). To describe the nodal properties of the network we computed the betweeness centrality attribute that allowed us to identify the network hubs. In the following, these measures will be defined with the traditional interpretation of general networks. However, their usefulness as relevant descriptors of functional (normal or pathological) brain states will become apparent in the next sections.

### Clustering Index (C)

The clustering index *C_i_* of a node ‘*i’* is defined as the number of existing connections between the node’s neighbors divided by all their possible connections. It is a measure of the inherent tendency to cluster nodes into strictly connected neighborhoods [72]. Nodes are considered neighbors when a connection between them exists, which is not reduced to a physical neighborhood concept. The clustering index for the whole graph G is defined as the average clustering around each node:
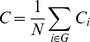
where N represent the number of nodes. Clearly, 0< *C* <1; and *C* = 1 if and only if the network is fully connected, that is, each node is connected to all other nodes.

### Characteristic Path Length (L)

The characteristic path length *L* of the graph G is the smallest number of connections required to connect one node to another, averaged over all pairs of nodes. It is a measure of the typical separation between two nodes (structures) *i* and *j* (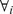,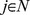), and it is defined as the mean of geodesic lengths 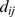 over all pairs of nodes.
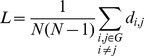



In the unweighted network context (the geodesic length 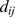 is defined as the number of edges along the shortest path connecting nodes *i* and *j* [71]–[73].

### Small-world Attribute (Sigma)

To examine the small-world properties, the values of characteristic path length (*L^real^*) and clustering index (*C^real^*) were compared with the same metrics estimated in random networks (*L^rand^, C^rand^*) with the same number of nodes, average degree (average of the degree over all node, where the degree 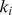 of a node ‘*i’* corresponds to the number of connections to that node), and degree distribution (probability that a randomly selected node has *_k_* connections) as the network of interest. We generated these random graphs using the random rewiring procedure described by Maslov and Sneppen (2002) [74], [75]. The small-world attributes can change with the correlation thresholds. When the threshold is increased, the resulting graphs will become sparser because some weaker connections will be dropped out leading to a decrease of the average degree.

The small-worldness network parameter σ, is defined as those having small average shortest path length, like random networks (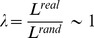), and high clustering index, much larger than random networks (
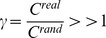
). These 2 conditions can also be summarized into a simple quantitative measurement, small-worldness, 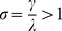.

#### 

##### Network efficiency

The concept of efficiency has also been expressed in terms of information flow [76]. That is, small world networks are very efficient in terms of global and local communication and they are defined to have high global *E_glob_* and local *E_loc_* efficiency. The global efficiency *E_glob_* of a graph G is expressed as:
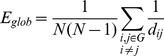



This measure reflects how efficiently the information can be exchanged over the network, considering a parallel system in which each node sends information concurrently along the network. On the other hand, the *E_loc_* of G is defined as the average efficiency of the local subgraphs:
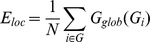
where *G_i_* is the subgraph of the neighbors of ‘*i’*. This measure reveals how much the system is fault tolerant, showing how efficient the communication is among the first neighbors of *i* when it is removed [76]. As above, nodes are considered neighbors when a connection between them exists, which is not reduced to a physical neighborhood concept.

### Nodal Centrality: Normalized betweenness Centrality (NBC)

The ‘betweenness centrality’ B_i_ of a node *i* is defined as the number of shortest paths between any two nodes that run through node *i* [77]. We measured the normalized betweenness centrality as b_i_ = B_i_/<B>, where <B> was the average betweenness of the network. b_i_ is a global centrality measure that captures the influence of a node over information flow between other nodes in the network. The hubs of the network are the regions with high values of b_i_. In our particular case, betweenness centrality b_i_ could be used to reflect the effects of the disease on the global roles of regions in the cortical CMRgl networks.

### Methodology to Explore Nodal betweenness Centrality (NBC) Differences between Groups. Hubs Selection

For each bootstrap sample of the CMRgl connectivity matrix the nodal NBC was computed in every single sparsity degree. Previously to this process, the largest component (see [78]) of all bootstrap samples of the CMRgl covariation matrices was computed. The minimum sparsity degree for the largest connected components (equal to the number of AAL structures) was used as an upper limit of sparsity degree range. This step guarantees that all nodal NBCs come from fully connected CMRgl networks. For every anatomical structure we assessed a NBC mean curve over the bootstrap samples. To test differences between groups a Kruskal Wallis (KW) nonparametric statistical test was used. We performed a Bonferroni adjustment to compensate for multiple comparisons. Those structures with significant corrected KW tests at least a 99% of the sparsity degree values were finally assumed statistically different. This procedure reduces dependences of the nodal NBC differences on a particular sparsity degree.

We find the mean NBC over the sparsity degree range and bootstrap samples (as in [79]), which consequently don’t depend on the thresholding process of the CMRgl covariation matrices. Hubs were selected as those with mean NBC superior to 1.5 (NBC>1.5) (similar to [11], [16], [65], [80]).

### Statistical Methodology to Study CMRgl Differences between Groups

In order to study CMRgl differences between groups a linear regression was performed at every AAL anatomical region using age, gender, age–gender interaction, group, education and the structure’s volume as independent variables and CMRgl as dependent variable. As before, the structure’s volume was introduced as a covariate in the linear regression in order to reduce the partial volume effects present in FDG-PET images. It was not found significant effect of the education variable on any regional CMRgl (p>0.05) despite the differences between AD and MCI groups reported in Table 1. Therefore this variable was not included in the final linear regression model. The variables age, gender, age-gender interaction and structure volume were finally considered as confounds. The effect of the group variable was evaluated contrasting the corrected CMRgl between pairs of groups using the statistic t-Student test. There were explored the hypothesis tests: NC versus AD, NC versus MCI and MCI versus AD. Since this process is performed for every AAL structure; a Bonferroni correction was used to adjust for multiple comparisons.

### Statistical Methods to Compare CMRgl Connectivity Matrices

In order to compare the CMRgl connectivity matrices between AD, MCI and NC groups, the correlation coefficients were converted into z values using Fisher’s r-to-z transformation. This procedure guarantees values with approximately normal distribution. We used the ‘Z’ statistic to compare the transformed z values in order to define the significance of the group differences in CMRgl correlations [81]. The Z statistic is calculated by: 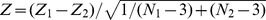, where *N_1_* and *N_2_* are the number of data used to calculate the correlation coefficients *r_1_* and *r_1_* transformed to *z_1_* and *z_2_* using the well-known ‘r’ to ‘z’ Fisher transformation: 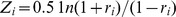, for *i* = 1,2. To adjust for multiple comparisons, a false discovery rate (FDR) procedure was performed at q value of 0.05 [82]. All possible combinations AD vs. MCI, AD vs. NC and MCI vs. NC were tested. This procedure has been used by previous authors for studying the topological organization of the brain networks in AD and MCI [11], [16].

### Methodology for Studying Differences in the CMRgl Covariations Across Brain Lobes

This study is carried out to investigate differences in the spatial distribution of the largest absolute CMRgl covariations between groups. For this purpose we used the anatomical subdivision of the brain in lobes proposed by Tzourio-Mazoyer et al. 2002 [64]. The first 1000 largest CMRgl covariations (24.96% out of total (4005)) were used for this study. We found the number of intra lobe CMRgl covariations in the Limbic, Frontal, Parietal, Occipital, Temporal lobes for each group and all bootstrap samples of the CMRgl connectivity matrices. A nonparametric Kruskal-Wallis statistic was used to test differences between groups corrected by multiple comparisons.

On the other hand, the intra-lobe CMRgl covariations (intra-lobe interconnectivity) were taken as the mean of the absolute correlation coefficient values among intra-lobe structures. As above it was performed for all bootstrap samples of the CMRgl connectivity matrices. This was used to study differences in CMRgl covariations (inter-connectivity) between NC, MCI and AD groups for each brain lobe. Finally, the nonparametric Kruskal Wallis statistics along with the multiple comparisons test were used to find differences between groups.

### Statistical Methods to Compare Global Network Properties between NC, MCI and AD Groups

Network properties (NP) of the CMRgl connectivity matrices were computed for a range of sparsity degree values and different bootstrap samples for the AD, MCI and NC groups. Thus, we had a set of Nboot = 300 curves each with 21 sparsity degree points for every network properties. The area under network attributes curves was obtained to contrast the global behavior of these measures using a Kruskal Wallis nonparametric one-way analysis of variance and multiple comparisons correction. The post hoc tests: AD vs. MCI, AD vs. NC and MCI vs. NC were performed. It is worth noting, that due to the topology of the NP curves is monotonic with the sparsity degree; the area is a suitable descriptor for characterizing its global performance. This descriptor has been used in previous studies [79], [83].

## Results

### Identifying Regions of Abnormal Glucose Metabolism in AD and MCI

**Figure 1:**
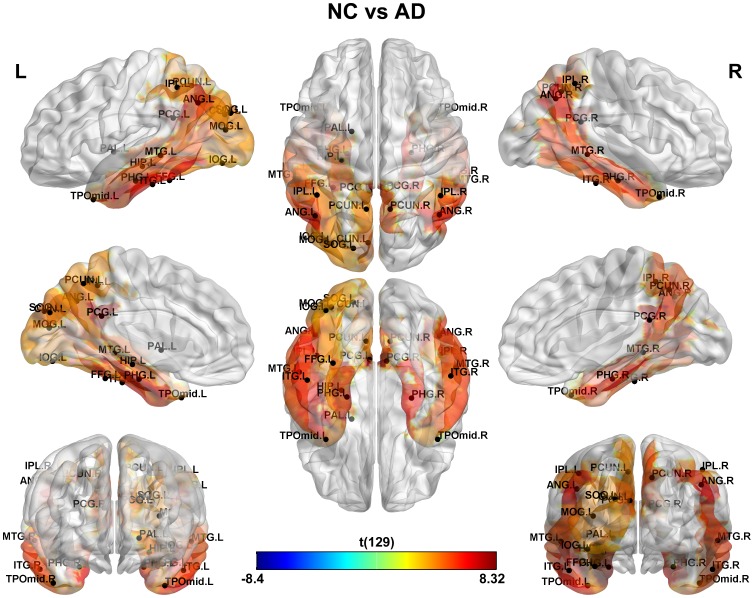
Differences in glucose metabolism between NC and AD groups. It is shown an extended area of hypometabolism in AD patients that include regions described previously in the literature such as those belonging to the limbic, temporal, parietal and occipital lobes.D.

**Figure 2:**
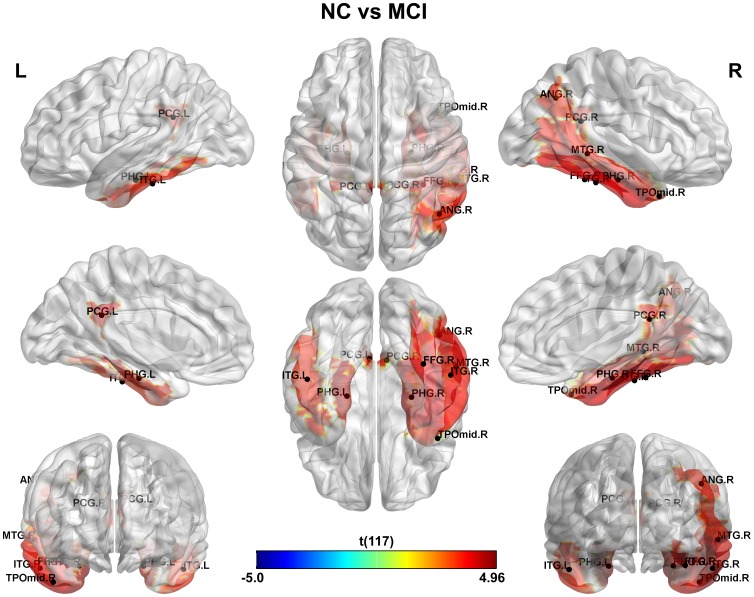
Differences in glucose metabolism between NC and MCI groups. It is depicted areas of glucose hypometabolism in MCI group respect to NC.

**Figure 3:**
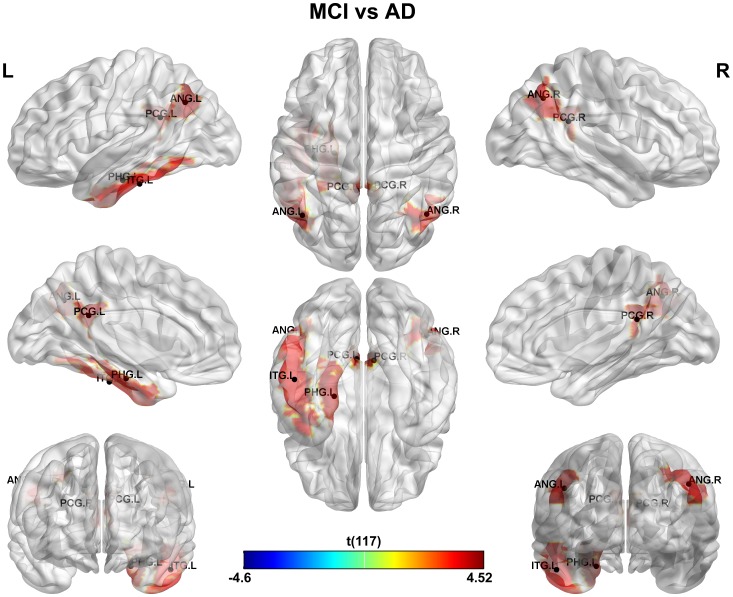
Differences in glucose metabolism between MCI and AD groups. It is shown glucose hypometabolism regions in AD group as compared to MCI.

This study is aimed at identifying the cerebral regions with disrupted glucose metabolism in AD and MCI pathologies. The main results can be found in Figures 1, 2 and 3 where the significant differences between groups: NC vs. AD, NC vs. MCI and MCI vs. AD were shown respectively (see Table S2 for a detailed tabulation of the CMRgl differences between groups). It is important to remark on the fact that only regions of glucose hypometabolism were found in AD and MCI groups as compared with NC (p-values<0.05), and in AD as compared to MCI (p-values<0.05).

AD group showed the higher number of regions with hypometabolism (23 regions) (see Figure 1) which are localized in temporal lobe bilaterally (middle and inferior temporal gyri), limbic (parahippocampal gyrus, posterior cingulate gyrus), parietal lobe and associative occipital structures. It is interesting to note the lateralization of the hypometabolism areas to the left hemisphere mostly in occipital lobe which include middle, inferior and superior occipital gyri, as well as the cuneus and fusiform gyrus.

On the other hand, the number of abnormal CMRgl in the MCI group was reduced to ten regions as compared to NC (see Figure 2). A bilateral pattern of glucose hypometabolism in structures belonging to limbic lobe (posterior cingulate and parahippocampal gyri) and temporal lobe (inferior temporal gyrus) characterized this group. However, a larger area of glucose metabolism damages were observed in the right hemisphere determined by structures such as angular, middle temporal and fusiform gyri as well as the temporal pole.

Finally, areas of glucose hypometabolism in AD group related to MCI were also identified. These cerebral regions (6 in total) were depicted in Figure 3. We found bilateral CMRgl reductions in limbic-parietal lobes regions such as angular and posterior cingulate gyri. Further areas like the parahippocampal and inferior temporal gyri showed a pattern of hypometabolism only in the left hemisphere.

### Evaluating Differences between AD, MCI and NC Groups in CMRgl Connectivity Matrices

**Figure 4:**
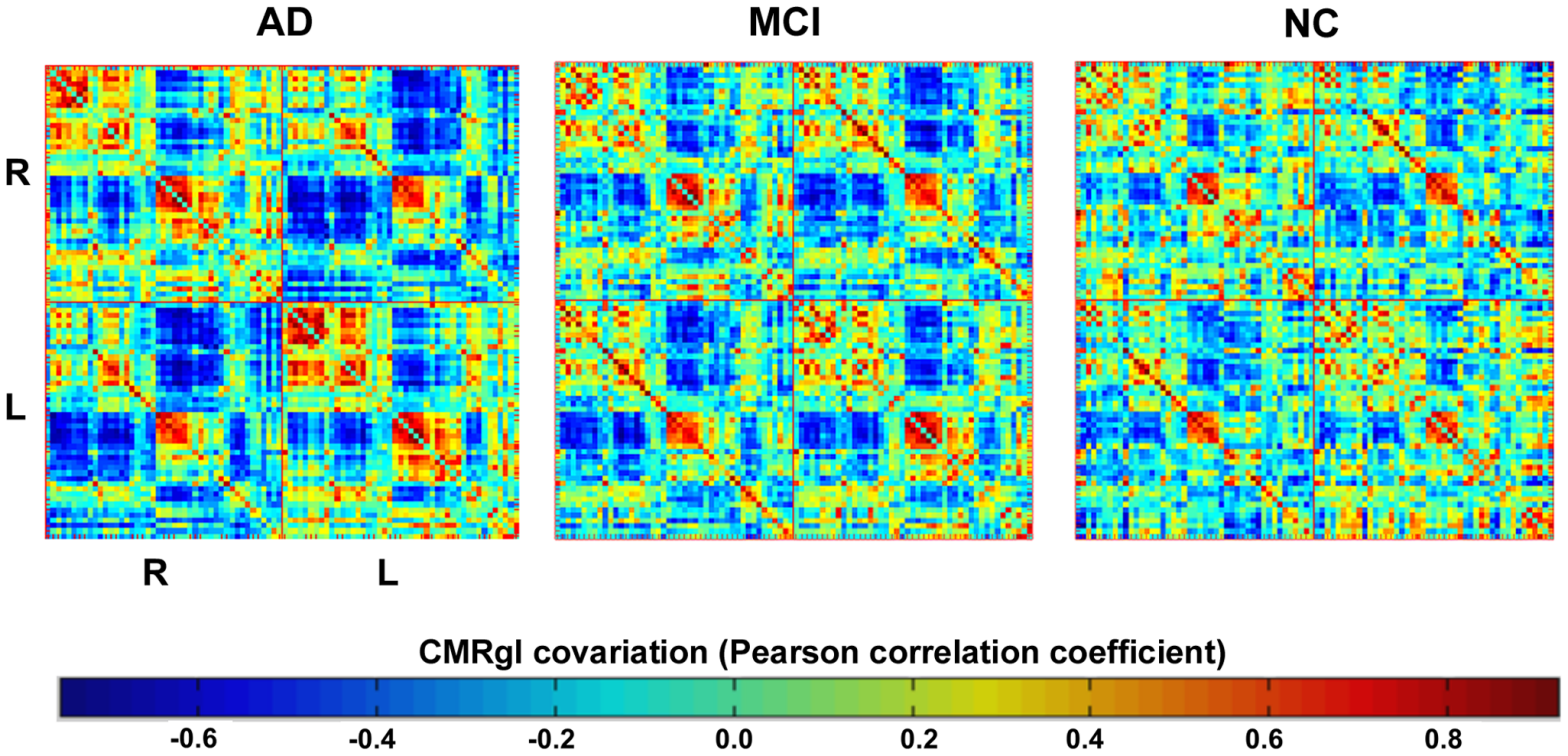
CMRgl connectivity matrices for AD, MCI and NC groups. The color bar indicates the value of the correlation coefficient coming from the glucose metabolism covariations among the 90 anatomical structures. ‘R’ and ‘L’ represent right and left hemisphere respectively. The CMRgl covariation matrices were organized first with structures belonging to the right hemisphere (‘R’ label). The ‘R-R’ and ‘L-L’ quadrants represent the intra-CMRgl covariations belonging to the right and left hemispheres respectively. The ‘R-L’ and ‘L-R’ quadrants depict the inter-hemispheric interactions, where CMRgl covariations between homologous structures can be observed.

**Figure 5:**
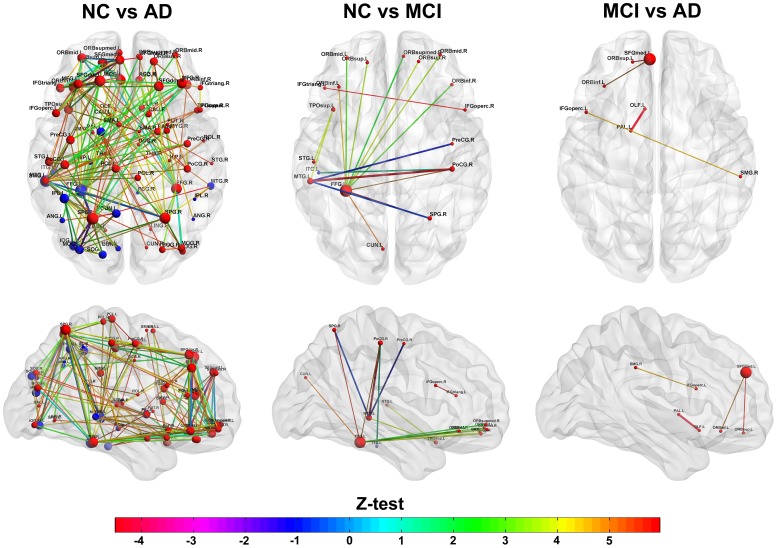
Differences between CMRgl connectivity matrices: NC vs. AD, NC vs. MCI and MCI vs. AD. The spheres (in red and blue) depict the anatomical regions involved in the connections differences. The sphere’s diameter represents the number of times each node is involved in the group differences. Lines illustrate the connections and its color and thickness the Z- statistics. The larger the CMRgl covariation differences the higher the Z statistics. Spheres in blue symbolize the anatomical regions with hypometabolism in AD and MCI groups as compared with NC.

After the previous analysis to identify those areas with reduced glucose metabolism in AD and MCI, it is important to explore the CMRgl covariations between anatomical regions (see Figure 4). Figure 5 depicts the patterns of CMRgl covariations statistically different between groups (NC vs. AD, NC vs. MCI and MCI vs. AD, p<0.05, FDR corrected) (see Table S3 and Text S1 for a complete list of these differences). The sphere’s diameter represents the number of times each anatomical region is involved in group differences. The highest number of differences was found between NC and AD groups (183 pairs of regions). NC and MCI groups were different in 17 pairs of regions whereas the smallest number of differences was found between MCI and AD with only 4 pairs of regions. It can be observed that frontal and occipital regions in AD (see Figure 5, ‘NC vs. AD’ panel) are involved in a considerable number of aberrant CMRgl covariations as compared to NC. With respect to MCI group, it is important to remark on the differences observed in the covariations between fusiform gyrus with the frontal regions (see Figure 5, ‘NC vs. MCI’ panel). The spheres in blue represent the structures with glucose hypometabolism which are implicated in CMRgl covariation differences (see Table S6 for full information). It is worth noting that only one structure with abnormal glucose metabolism is involved in NC vs. MCI differences. In MCI vs. AD was not found any coincident structure.

Large co-variations in local glucose metabolism between homologous regions were present in AD, MCI and NC groups (see Figure 4 and 5). This result is in accordance with previous studies in healthy and pathological subjects [11], [16], [29], [30], [63], [65].

Differences in the CMRgl connectivity matrices between MCI and AD groups involved structures that included the olfactory cortex, the pallidum nucleus and the superior and inferior frontal gyri. Additionally, NC and MCI groups showed significant differences in structures such as the temporal pole, middle temporal gyrus, postcentral and precentral gyri, superior, inferior and middle frontal gyrus. However, significant changes between NC and AD groups were found in the superior, middle and inferior frontal gyrus, superior parietal gyrus, precentral and postcentral gyrus, paracentral lobule, middle temporal gyrus, angular and fusiform gyri.

### Exploring the Spatial Distribution of the Strongest CMRgl Covariations. Differences in Intra-lobe CMRgl Covariations between AD, MCI and NC Groups

We performed an exhaustive study to characterize the spatial distribution differences of the strongest CMRgl covariations among groups. Differences in intra-lobe CMRgl covariations were also explored (for methodological details see in Materials and Methods section, subsection: ‘*Methodology for studying differences in the CMRgl covariations across brain lobes’*).

**Figure 6:**
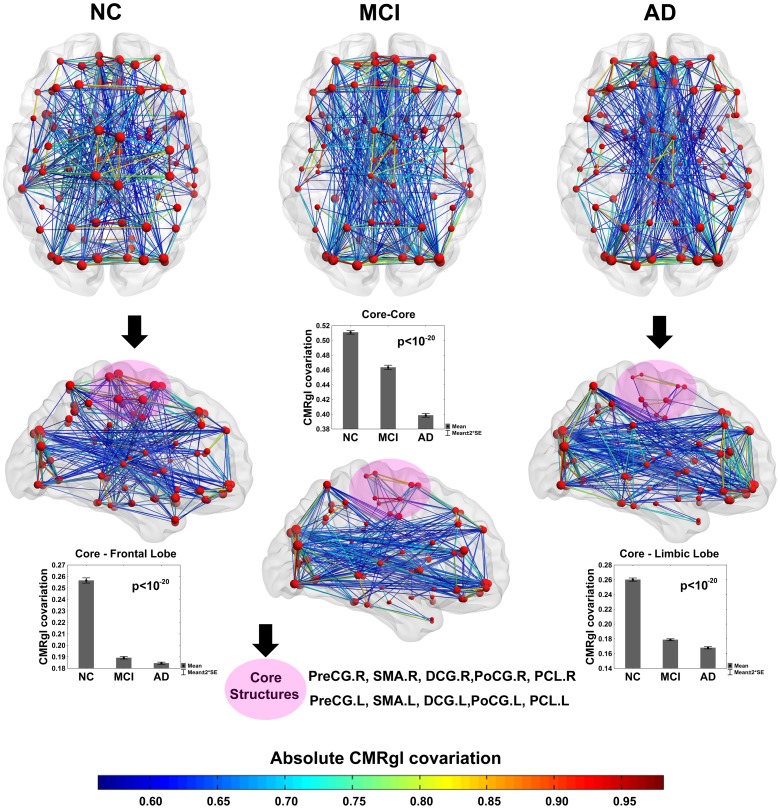
CMRgl networks for NC, MCI and AD groups. The 500 strongest CMRgl covariations are represented. The line color indicates the absolute CMRgl covariation strength. Arrows in black along with the area shaded in pink indicate a set of regions ‘Core’ comprising the following structures: PreCG.R, SMA.R, DCG.R, PoCG.R, PCL.R, PreCG.L, SMA.L, DCG.L, PoCG.L and PCL.L. The ‘Core’ set shows a loss of CMRgl covariations from NC to AD where MCI has an intermediate position (see the bar graph at the center: ‘Core-Core’ panel). This phenomenon is also observed between Core and limbic lobe (‘Core –Limbic’ panel) as well as between Core and frontal lobe (‘Core-Frontal’ panel). The sphere’s diameter denotes the CMRgl covariation of each node with the rest of the network. The error bars in different panels represent twice the standard error. The bar height denotes the CMRgl covariation.

Firstly, we identified a set of structures with distinctive differences between groups in the number of largest CMRgl covariations (denoted here as ‘Core’, see Figure 6, area shaded in pink and black arrows). ‘Core’ comprises regions belonging to the central area, medial surface of frontal lobe and limbic areas in both hemispheres, these were: precentral gyrus (PreCG.R, PreCG.L), supplementary motor area (SMA.R, SMA.L), median cingulate and paracingulate gyri (DCG.R, DCG.L), postcentral gyrus (PoCG.R, PoCG.L), and paracentral lobule (PCL.R, PCL.L). It can be observed significant abnormal behavior (p<10^−20^) of the CMRgl covariations among Core structures in AD and MCI respect to NC. It is interesting to mention that MCI shows an intermediate position between NC and AD. A further remarkable result is the aberrant CMRgl covariations between ‘Core’ and limbic, frontal lobes present in AD group (p<10^−20^). Once more, it is noteworthy that MCI has a middle position. Previous to this analysis we separated from limbic and frontal lobes the structures belonging to ‘Core’.

**Figure 7:**
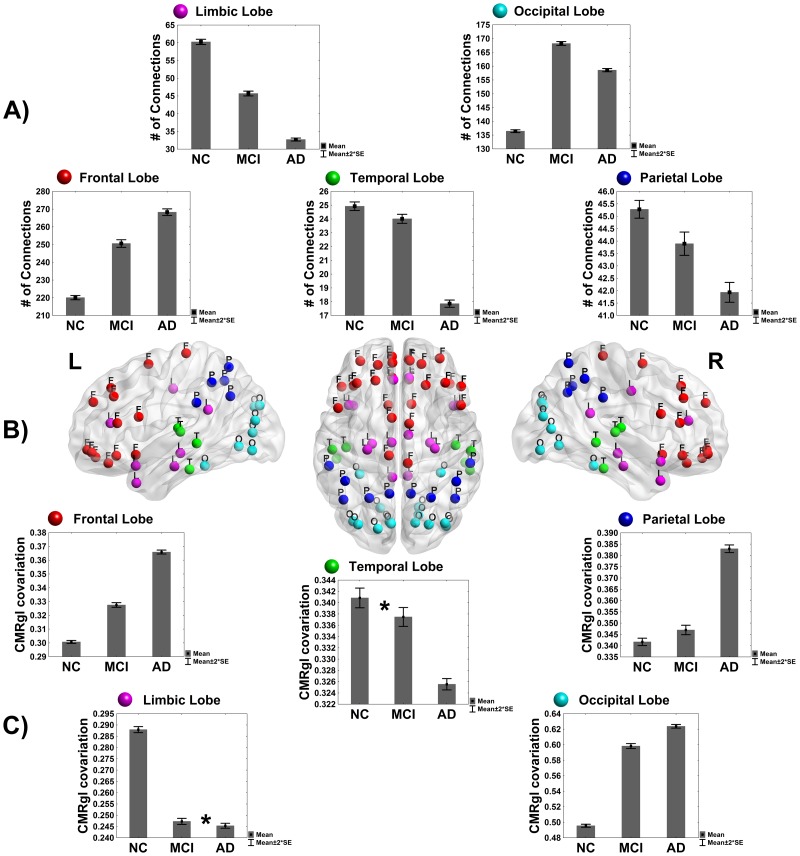
Intra-lobe CMRgl covariations statistics, distribution of the largest CMRgl covariations. A) Differences between groups by lobes in the number of absolute correlation coefficients (bar height) among the 1000 largest CMRgl covariations. In each brain lobe, there were found differences between groups (p-value<0.05). B) Distribution of nodes by lobes in different colors (as defined in Tzourio-Mazoyer et al. 2002). Red: Frontal Lobe; green: Temporal Lobe; cyan: Occipital Lobe; magenta: Limbic Lobe; blue: Parietal Lobe. C) Differences between groups in terms of intra-lobe CMRgl covariations obtained as the mean of the absolute value of the correlation coefficients among all intra-lobe structures. The star in the ‘Temporal lobe’ panel means that there was not difference in intra-lobe CMRgl covariations between NC and MCI (p>0.05). Likewise in the limbic lobe the star denotes that there was not difference between MCI and AD in terms of intra-lobe CMRgl covariations. The error bars in different panels represent twice the standard error.

Figure 7 depicts the group differences in the distribution of the 1000 strongest CMRgl covariations over brain lobes (see panel A, p<10^−20^). It is also shown the intra-lobe CMRgl covariations differences in Frontal, Parietal, Limbic, Occipital and Temporal lobes (panel C). AD and MCI groups showed aberrant behaviors in the temporal, parietal and limbic lobes, in which MCI takes a middle position. This result is consistent with the spatial distribution of abnormal glucose metabolism found in both groups (see Figures 1 and 2). Though, in the frontal and occipital lobes an opposite pattern is found with an increasing number of strongest CMRgl covariations for AD and MCI as compared to NC (p<10^−20^). Note that structures belonging to these lobes were involved in an important number of differences between CMRgl connectivity matrices of AD and MCI groups as compared with NC.

The significant differences in intra-lobe CMRgl covariations are depicted in Panel C. We found AD showing higher intra-lobe CMRgl covariations than NC in frontal, parietal and occipital lobes. MCI group occupies the intermediate position as above. In contrast, AD depicted limbic and temporal lobes with significant loss of the intra-lobe CMRgl covariations respect to NC. The MCI group has varying patterns with no significant alterations in the temporal lobe relative to NC, whereas in the limbic lobe were found no differences respect to AD. Precisely the limbic and temporal lobes showed the highest glucose metabolism deficits in AD and MCI groups. In general, these results might suggest that in MCI and AD pathologies could be coexisting temporarily compensatory mechanisms and disease related-processes.

We also studied the CMRgl covariations among brain lobes in all groups. The CMRgl covariations between occipital and other lobes were the highest for AD and MCI (p<10^−20^) (see Figure S3 for methodological aspects and results), that could justify the presence of higher number of hubs in these groups respect to NC. The MCI group occupied, as before, the intermediate position.

Finally, the CMRgl covariations among regions with hypometabolism in AD were studied across different groups (see Figure 1 and Table S2 for the list of structures). This analysis provides us the information about the strength of the functional association among structures with possible shared vulnerability from normal to AD. The AD showed the highest CMRgl covariations among these structures, whereas the NC the lowest value. The MCI perform in-between NC and AD. All post hoc tests were significant (p<0.05) (NC vs. AD, NC vs. MCI; MCI vs. AD) (see Figure S1 for details).

### Global Network Properties in AD, MCI and NC Groups

Recently, the presence of small-world topology in anatomical networks of normal elderly subjects, MCI and AD patients has been found [11], [16]. These studies demonstrated a less optimal behavior in morphological networks related to covariations of volume and cortical thickness in AD and MCI, showing an increment in clustering index and shortest path length. In our case, the global properties (clustering index, characteristic path length, local efficiency, global efficiency and small world attribute) of the CMRgl connectivity matrix were calculated over a range of sparsity values in order to study differences in network organization features between NC, MCI and AD groups.

**Figure 8:**
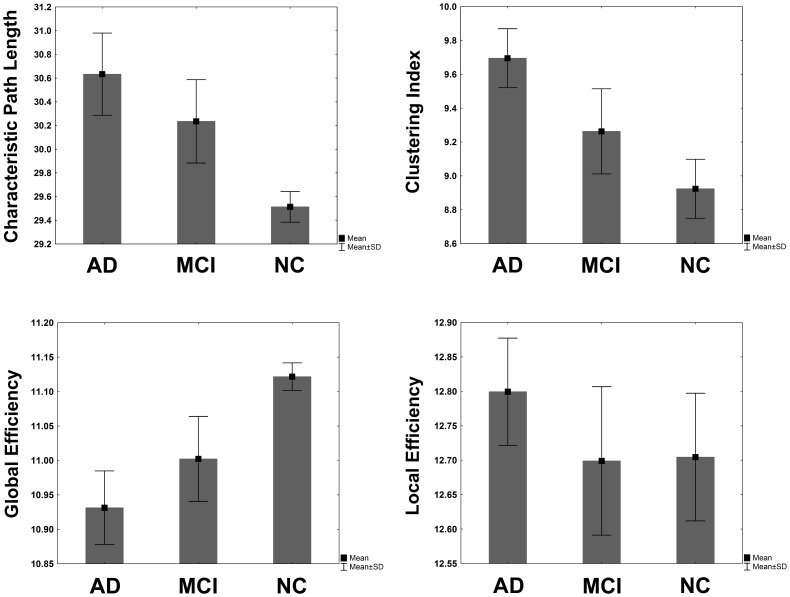
Area under the curves of global properties of the CMRgl covariation networks. Bar height represents the mean of each network properties for AD, MCI and NC groups. Error bars represent standard deviations. AD networks show higher characteristic path length, clustering index and local efficiency values than MCI and NC (p<0.05). The AD patients show lower global efficiency than MCI and NC (p<0.05). In general, MCI network properties have an intermediate position between AD and NC except for the local efficiency.

Figure 8 illustrates the area under the global network attributes curves in AD, MCI and NC groups. The Kruskal-Wallis nonparametric revealed significantly differences between groups (in all cases p<10^−31^) for characteristic path length, clustering index, local efficiency, global efficiency and sigma attributes.

The post hoc comparisons revealed significantly higher characteristic path length, clustering index and local efficiency values in AD networks than in MCI and NC (in all cases: Multiple Comparisons p-value (2-tailed)<10^−20^). In contrast, the AD group showed lower global efficiency than MCI and NC (Multiple Comparisons p-value (2-tailed) <10^−20^) whereas MCI network properties has a middle position between AD and NC except for the local efficiency (AD vs. MCI and AD vs. NC: p-value (2-tailed) <10^−20^; MCI vs. NC: p-value (2-tailed) = 0.92). With respect to sigma attribute, NC depicted higher values than MCI and AD (see Figure S2 for details). The post hoc tests: NC vs. AD and NC vs. MCI where statistically significant (p-value (2-tailed) <10^−20^). However, AD vs. MCI showed no significant differences (p-value (2-tailed) = 0.58)). These results suggest that AD and MCI in different degree could be associated with disruptive system integrity in large scale brain networks expressed by the loss of balance between network segregation and integration processes. [11], [16] [10], [12]–[15], [17].

Similar to Yao et al. 2010 study we found that the MCI network behaves between AD and NC networks in all attributes (p-value (2-tailed)<10^−20^) except for local efficiency and sigma where no significant differences were observed in NC vs. MCI and MCI vs. AD respectively. Our findings provided additional support for the hypothesis that cortical networks have a further loss in efficiency during the progression from normal aging to AD [11], [16].

### Studying Differences in Normalized betweenness Centrality (NBC) between Groups

The normalized betweenness centrality could be interpreted as the influence of a specific structure over the ‘*information flow*’ between other regions in the CMRgl network. Differences observed in this network attribute could provide evidences about the local alterations related to AD and MCI pathologies on global roles of the anatomical structures in the CMRgl network functioning.

**Figure 9:**
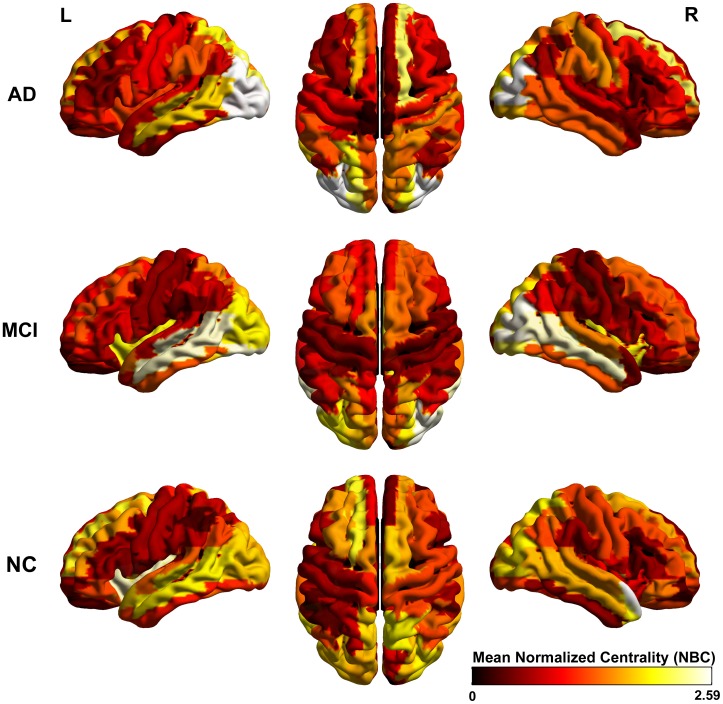
Mean normalized betweenness centrality (NBC) for AD, MCI and NC groups. For each anatomical structure a mean curve of the NBC (over 300 bootstraps) along the sparsity degrees was assessed. The mean of the NBC curve over the number of sparsity degrees yields the mean NBC depicted in this figure. It can be observed in AD group prominent NBC values in occipital regions. The node’s NBC were mapped onto the cortical surfaces using the BrainNet Viewer package (http://www.nitrc.org/projects/bnv).

Figure 9 shows the mean NBC for all regions in each group. We found in NC high NBC values in regions such as the insula, parts of the occipital lobe, middle temporal and superior frontal gyri. Though, the highest NBC values in AD group were located in the frontal and occipital lobes, whereas in MCI group these regions were found in the middle temporal gyrus and occipital areas.

**Figure 10:**
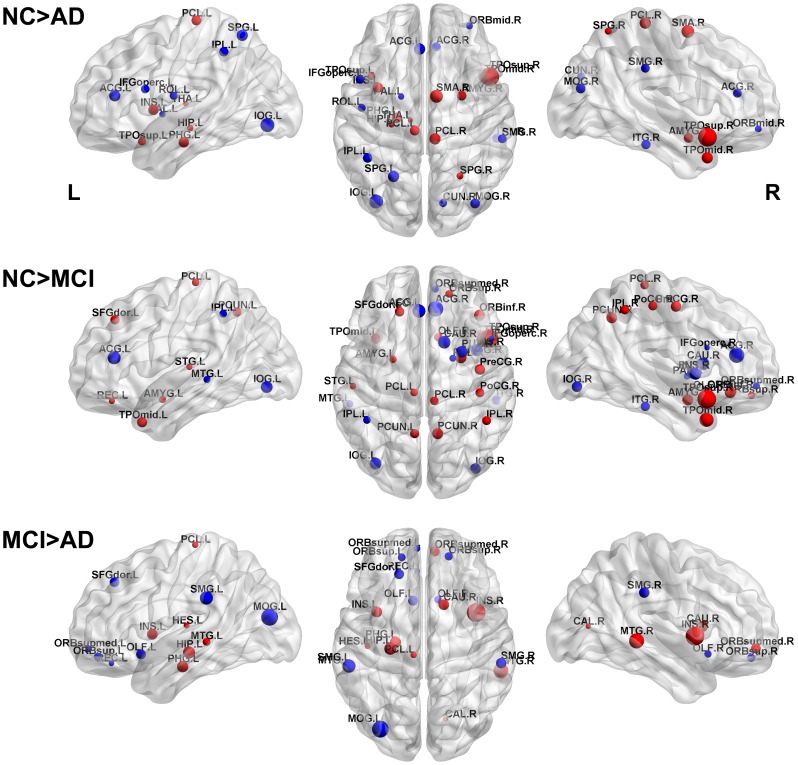
Statistical differences in nodal normalized betweenness centrality (NBC) between groups. First row: NC versus AD, second row: NC versus MCI, third row: MCI versus AD. The sign of the statistical test was represented in red and blue spheres for positive and negative effects respectively. Red spheres at the first row symbolize regions where NC was statistically higher than AD in the NBC, the opposite for blue spheres. The sphere diameter denotes the size of the difference effect. The NBC differences were mapped onto the cortical surfaces using the BrainNet Viewer package (http://www.nitrc.org/projects/bnv).

Additionally, we studied the NBC differences between groups depicted in Figure 10 and tabulated in Table S4 (see Materials and Methods section for methodological aspects). It was observed a decreased NBC in AD respect to NC group in the right supplementary motor area, superior parietal gyrus, left parahippocampal gyrus, left hippocampus, insula and amygdala (see Figure 10, panel ‘NC>AD’). In contrast, in AD other regions showed an increased NBC such as left superior and inferior parietal gyrus, right and left anterior cingulate gyri, right cuneus, inferior temporal gyrus and middle occipital gyrus.

Respect to NC the MCI group depicted NBC reduction in the following structures: right and left paracentral lobe, left superior frontal gyrus, right and left precuneus, left amygdala, left superior temporal gyrus, right superior parietal gyrus and right supplementary motor area (see Figure 10, panel ‘NC>MCI’). An increased NBC in MCI respect to NC was detected in the right and left anterior cingulate gyri, left middle temporal gyrus, left and right inferior occipital gyri and right inferior temporal gyrus among others.

Finally, MCI and AD groups showed the least number of NBC differences (see Figure 10, panel ‘MCI>AD’). This result corresponds with the CMRgl connectivity matrices differences between these two groups. AD group depicted significant NBC reductions in regions related to memory process such as: left parahippocampal gyrus, left hippocampus, left and right middle temporal gyri, Heschl or transverse temporal gyrus. Likewise there were found differences in the right and left insula, right medial orbital part of the superior frontal gyrus, left paracentral lobe among others. It is noteworthy the fact that the differences between these groups were located mainly in the left hemisphere which agrees with the glucose metabolism asymmetry pattern previously found (see Figure 3). In contrast, regions in AD with increased NBC were found in the left middle occipital gyrus, left and right supramarginal gyrus, left superior frontal gyrus, left and right orbital part of the superior frontal gyrus and left and right olfactory cortex. It is important to point out that these anatomical structures were located principally in the frontal and occipital lobes where significant increases in CMRgl covariations have been previously reported in our results (see Figure 7, panel C).

### Exploring Regions with Concurrent NBC Changes and Disrupted Glucose Metabolism

**Figure 11:**
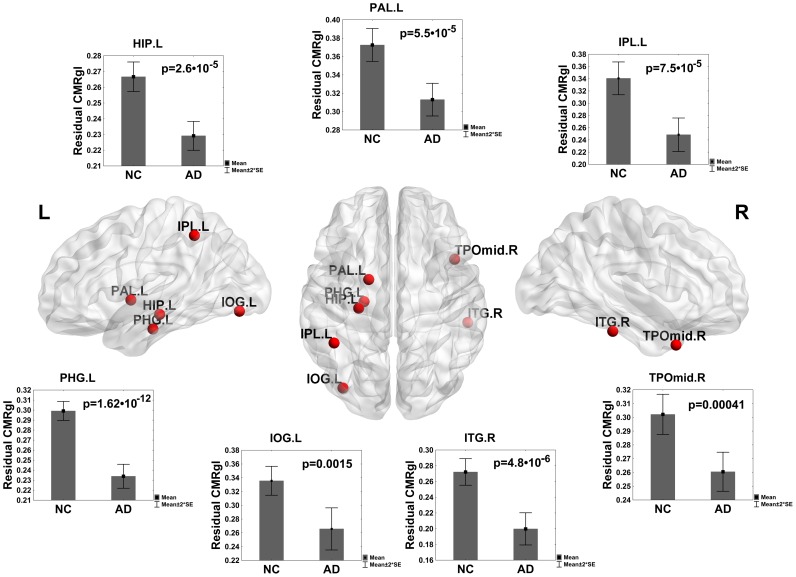
Regions with glucose hypometabolism in AD related to changes in betweenness centrality. Plot of regions found with glucose hypometabolism in AD as compared to NC presenting simultaneously changes in betweenness centrality. The center of the figure shows the spatial localization of these structures. It is plotted the residual of CMRgl covariations (bar height) of the linear regression for each group. Nodes were mapped onto the cortical surfaces using the BrainNet Viewer package (http://www.nitrc.org/projects/bnv).

It is consistent to assume that local alterations in glucose metabolism (hypometabolism) detected through FDG-PET imaging in AD and MCI could be one of the pathophysiological mechanisms to explain aberrant behaviors of the anatomical structures from its global roles in the CMRgl network functioning. To this end, we explored the anatomical regions that concurrently showed NBC alterations and glucose hypometabolism in AD and MCI groups. The results for AD are shown in the Figure 11. These structures were: right temporal pole part of the middle temporal gyrus (TPOmid.R), right inferior temporal gyrus (ITG.R), left hippocampus (HIP.L), left parahippocampal gyrus (PHG.L), left inferior occipital gyrus (IOG.L), left inferior parietal (IPL.L) and left pallidum (PAL.L). However, in the MCI group only a couple of structures were found: right temporal pole part of the middle temporal gyrus (TPOmid.R) and right inferior temporal gyrus (ITG.R).

### Identification of Hubs in the CMRgl Networks of AD, MCI and NC Groups

To identify the network hubs, we select those cortical regions with NBC values above 1.5 (see Materials and Methods). This means that hubs have at least 1.5 times the network’s mean NBC.

**Figure 12:**
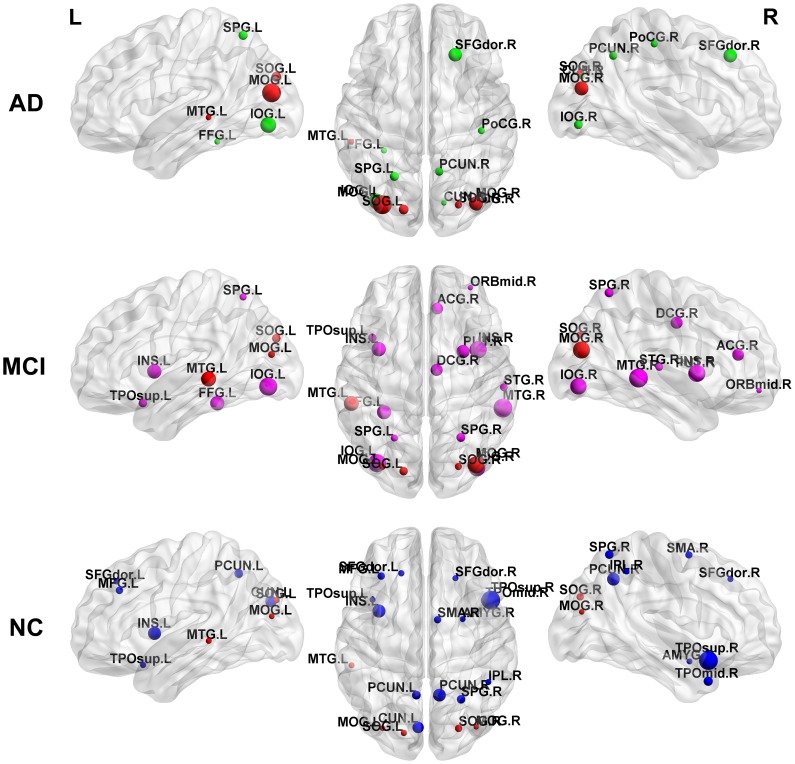
Hubs distribution in AD, MCI and NC groups. First row: AD hubs, second row: MCI hubs, third row: NC hubs. In red are represented the common hub regions. These were: SOG.L, MOG.L, SOG.R, MOG.R and MTG.L. The sphere diameter denotes the nodal betweenness centrality (NBC), in this case NBC>1.5. Nodes were mapped using the BrainNet Viewer package (http://www.nitrc.org/projects/bnv).

We found 19 hubs in NC and MCI groups, representing 21% of the total number of anatomical structures. These were the lowest number in AD group with 13, which represents the 14% of the total number of regions. The anatomical localizations of these regions were different across groups (see Figure 12 and Table S5). Specifically, AD’s hubs were predominantly located in both hemispheres in the lateral and medial surface of the occipital regions (8 out of 13). MCI’s hubs were observed at the lateral and medial surface of the occipital, limbic lobe, lateral surface of the temporal and parietal lobes. In NC group we found that lateral and medial surfaces of the occipital, frontal, temporal and parietal lobes, had the largest number of hubs. It is important to note that the highest number of hubs identified in all groups was predominately located in the association cortex which receives inputs from multiple other cortical regions as described by Mesulam (2000) [84].

### Identifying Hub Regions in NC with Abnormal Glucose Metabolism in AD and MCI Pathologies

**Figure 13:**
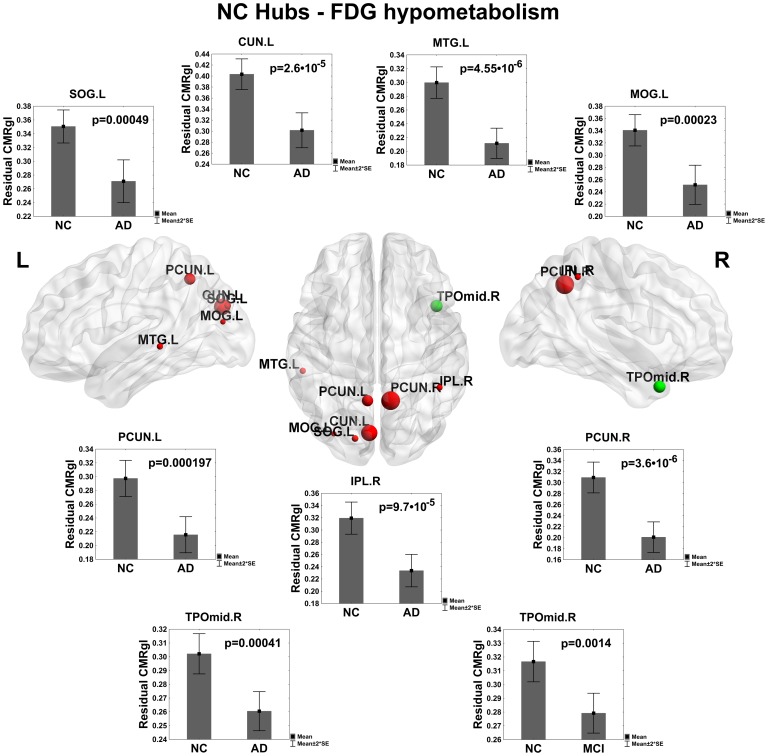
Hub regions in NC with hypometabolism in AD and MCI groups. Plot of hub regions in NC found with glucose hypometabolism in AD and MCI. The center of the figure shows the anatomical localization of these structures. The spheres in red denote the structures in AD with hypometabolism. The sphere in green represents regions found with hypometabolism in both AD and MCI groups. The sphere diameter denotes the nodal betweenness centrality value (NBC>1.5).

A further important aspect explored in our study was whether the extended hypometabolism areas observed in AD and MCI affected hub regions considered crucial to an ‘efficient communication’ in the complex CMRgl networks. For this purpose, we found those areas with hypometabolism in AD and MCI that were identified as hubs in NC (see Figure 13).

The structures found in AD were: right inferior parietal (IPL.R), right precuneus (PCUN.R), right temporal pole part of the middle temporal gyrus (TPOmid.R), left cuneus (CUN.L), left superior occipital gyrus (SOG.L), left middle occipital gyrus (MOG.L), left precuneus (PCUN.L) and left middle temporal gyrus (MTG.L). In MCI only the middle temporal gyrus (TPOmid.R) structure met this condition.

## Discussion

In this paper, we have studied the topological organization of the glucose metabolism covariations between cerebral regions through the resting-state FDG-PET neuroimaging technique. The used methodology is based on applying graph theory analysis to the CMRgl networks obtained after the correlations between glucose uptakes in every pair of 90 cerebral regions are explored. Similarly to previous works, we considered that these covariation patterns reveal information about the organization of the functional brain networks, particularly the CMRgl networks [29], [31], [33], [51], [85]. To our knowledge, this is the first time that graph theory is used to explore the CMRgl networks in Mild Cognitive Impairment (MCI) and Alzheimer disease (AD) patients as well as in elder normal subjects. Our main findings could be summarized as follows: 1) AD and MCI showed a pattern of abnormal glucose metabolism in regions previously reported in the literature; 2) MCI and AD related changes in CMRgl covariations were observed as compared with normal subjects; 3) The CMRgl network attributes (global and local) showed differences in AD and MCI patients as compared to NC suggesting disease-related alterations in the large scale networks; 4) the MCI’s network showed intermediate topological attributes supporting the view of MCI as a transitional stage between normal aging and Alzheimer disease. 5) Hub regions of the CMRgl networks in AD, MCI and NC were different in number and spatial distribution. In the next subsections we will discuss these issues in more detail.

### Abnormal Glucose Metabolism in AD and MCI

The ^18^F-FDG-PET imaging at rest allows characterizing quantitatively the basal local metabolism of the synaptic terminals in astrocyte-neuron functional unit [86], [87]. For a specific region a decline in the glucose uptake means either a reduction in number of synapses or reduced synaptic metabolic activity. It has been demonstrated in previous investigations that FDG-PET findings precede neurodegeneration by showing synaptic dysfunction before cellular loss; therefore this technique can detect early neuro-pathophysiological processes in AD and MCI outperforming, in this sense, the structural MRI.

In our study, we found that both MCI and AD are associated with significant CMRgl reductions in brain regions preferentially affected by the disease. The AD patients displayed more extended areas of hypometabolism than MCI as numerous studies have previously shown [23], [88], [89]. The AD group showed a reduced glucose metabolism as compared with NC in the so-called association cortex that include temporo-parietal, posterior cingulate and occipital associative regions (see Figures 1, 2 and 3, for full list see Table S2) [89]–[94]. These glucose hypometabolism patterns comprise areas involved in memory processing and structurally and functionally related to the default mode network (DMN). DMN is a network that has been consistently found to be impaired during resting state in MCI and AD [95]–[97]. In addition, the medial and lateral visual areas and fusiform gyrus are involved in face recognition and spatial navigation, and impaired visuo-spatial information processing is thought to develop early in AD [98]–[100].

There were not found regions with significant damaged glucose metabolism in frontal associative cortices, which correspond (as reported in previous papers) to a later phenomenon in the course of AD. Although, some frontal regions showed marginal significant hypometabolism in AD and MCI prior to applying Bonferroni correction. This result warns that early frontal glucose metabolism declines could be arising in our data sample.

In summary, our results depicted once more the excellent reproducibility of the FDG-PET technique to detect glucose hypometabolism patterns related to AD and MCI diseases. For that reason, the pattern of temporo-parietal hypometabolism is now considered a reliable hallmark of AD [87].

During next sections we attempt to discuss how the disrupted CMRgl network organization in AD and MCI could be modulated by the abnormal glucose metabolism patterns observed in these brain pathologies.

### Altered CMRgl Co-variation Patterns in MCI and AD Patients

Recently, it has been demonstrated that in some cases the local physiological states (characterized by univariate variables, in our case regional glucose metabolism) modify the capacity of one region to interact with other brain structures (characterized by more complex variables: bivariates and multivariates) [67], [101], [102].

Therefore, it could be thought that disruptions of local function and/or anatomical integrity in one area could modulate the underlying physiological processes operating in other cerebral structures with which it interrelates. In addition, local abnormal processes in many cases could influence aberrant functional associations among different regions [5], [67], [101]. Some of our results support this idea. Firstly, we found that: the larger the areas with abnormal glucose metabolism the higher the number of CMRgl covariations differences. For example, the AD showed the largest number of areas with abnormal glucose metabolism (23 out of 90), followed by MCI (10 out of 90) and the differences between MCI and AD was reduced to 6 structures. Concurrently, the AD and NC were different in 183 CMRgl covariations (see Figure 5 and Text S1); the MCI group as compared with NC showed 17 differences and finally the MCI and AD were only different in four occasions. Secondly, the largest number of areas with hypometabolism in AD was found in the left hemisphere where the number of aberrant CMRgl covariations was the largest. Third, structures with disrupted glucose metabolism were involved in 37.16% (68 out of 183) of the aberrant CMRgl covariations in AD respect to NC. Fourth, the 9.84% (18 out of 183) of the abnormal CMRgl covariations related to AD disease were among structures with significant glucose hypometabolism.

Similar to previous studies, we found significant positive CMRgl covariations between different anatomical regions [29]–[31]. It have been suggested that this phenomenon is explained by different ways: 1) a real functional association between anatomical structures; 2) the common influence exerted by one structure over a set of regions modulating theirs metabolic fluctuations, or 3) the compromise of a set of regions in a common pathological process or shared vulnerability [29], [68], [103]. Consequently, a decreasing of positive CMRgl covariations could imply a significant reduction in the functional association [31].

We observed significant increments in positive CMRgl covariations in AD and MCI in the frontal lobe (see Figure 5, 6 and Figure 7 Panel C: Frontal Lobe). This phenomenon could be explained at least by two possible mechanisms either operating at the same time or not. Firstly, these regions can have shared metabolic failures [104]. It may not be our main factor in view of the fact that was not found any frontal region with significant abnormal glucose metabolism in our data sample (see Figure 1, Figure 2 and Table S2). Even so it should not be entirely discarded since some abnormal neurodegenerative processes can be arising yet not detected by the FDG-PET technique. The hypometabolism in frontal regions is classically considered to be a late phenomenon in the course of AD. However, based on our results, the second and more reliable possible mechanism to explain the increased CMRgl interconnections in the frontal lobe could be related to compensatory effects or cognitive resource allocation processes [105]–[107]. These compensatory mechanisms could be considered very important since they permit the AD and MCI patients to have supplementary cognitive resources to approach a normal level [16]. These findings are similar to those of recent studies using resting-state fMRI which show AD patients with increasing intra-frontal and frontal-prefrontal functional connectivity [13], [15], [108], [109]. Hence, a typical AD pattern emerges in which frontal regions depict relatively higher mean levels of CMRgl synchronization.

On the other hand we also found increased CMRgl covariations among structures of the occipital lobe in AD and MCI (see Figure 7 Panel C: Occipital lobe). In our opinion and contrarily to frontal lobe the shared metabolic failure among structures is the most reliable mechanism operating in this case. The main fact pointing to this (see Figure 1) is that almost half of the regions belonging to occipital lobe (associative occipital cortex) presented glucose metabolism injuries as part of the AD neurodegenerative processes. We hypothesized that these areas with glucose hypometabolism could additionally trigger compensatory processes acting simultaneously with shared metabolic failures. This could also justify the disproportioned increasing of CMRgl covariations in this lobe. It is worth noting that AD, MCI and NC showed only positive CMRgl covariations among occipital structures. Therefore, it could be thought that the extension of the occipital glucose metabolism damages may modulate the strength of the aberrant CMRgl covariations in AD. The increased CMRgl covariations in MCI as compared with NC could be explained by compensatory mechanisms either acting simultaneously or not with early glucose hypometabolism processes not yet detectable by the FDG-PET technique (there were not found any occipital region with hypometabolism in MCI, see Figure 2: NC vs. MCI). Taking into account that MCI is considered a transitional stage between NC and AD, these results would suggest the possibility of detecting early increased CMRgl covariations before the evidence of glucose metabolism damages (regional hypometabolism). It is important to point out that the increased CMRgl covariation in frontal and occipital lobes could be candidate markers for an early detection and characterization of the MCI and AD pathologies. In addition this highlights the importance of using the intra-lobe CMRgl covariations variable to study these diseases.

Another important aspect to discuss is the presence of a set of structures ‘Core’ with intra-CMRgl covariations deficits in AD and MCI (see Figure 6). Core’s structures belong to different brain lobes (frontal, central and limbic bilaterally). It is noteworthy to mention that none of these regions showed abnormal glucose metabolism, which is a further example (as above) showing that local changes in physiological variables (univariate variables) can be insufficient for detecting disease related changes [67]. In addition, this highlights the idea that complex multivariate measures (i.e. co-variations) in many cases are more sensitive in characterizing brain pathologies as it has been found studying other neurological diseases like Schizophrenia [67], [101], [102]. We observed the CMRgl covariations ‘Core’-frontal lobe and ‘Core’-limbic lobe were also damaged in AD (see Figure 6), in which once more MCI has an intermediate position. It is important to remark on the fact that all structures belonging ‘Core’ except one were involved in disrupted CMRgl covariations with other external regions. We hypothesize this phenomenon could be one of the factors influencing the low CMRgl covariations between Core’s structures. It is worth noting that limbic lobe showed a high number of structures with hypometabolism, and the frontal lobe presents incipient abnormal glucose metabolism processes. Aditionally, the composition of Core with structures belonging to different brain lobes brings to light the damaged CMRgl covariations among lobes present in AD and MCI (see Figure S3). Finally, we recommend that covariations among Core’s structures should be investigated with other physiological and morphological variables (i.e. volume, cortical thickness, surface area, blood oxygenation, cerebral blood flow) to explore the specificity and sensibility of this set to characterize AD and MCI. Furthermore, a deeper study to find other clusters of regions, to aid the early detection of AD related-neurodegenerative processes should be carried out in future investigations.

It is important to remark on the presence of negative CMRgl covariations between fusiform gyrus and frontal regions in AD patients, contrary to NC that showed positive interactions between these brain areas. It is known that both cerebral regions are functionally and anatomically connected via the visual ventral stream[110]–[112]. The exact physiological or physiopathological mechanism underlying negative covariations is less evident, but it does correspond to a decreased metabolism in one region when another is metabolically more active [29], [103].We found precisely the fusiform gyrus presenting glucose hypometabolism that could be the source of the aberrant coupling with frontal areas. This result was observed partially in an earlier work (Horwitz et al. 1987) [31], where the authors detected an inversion from positive in NC to negative CMRgl covariations between frontal and occipito-temporal regions in AD.

Interestingly, when MCI and NC groups were compared, once more the negative CMRgl covariations arose between fusiform gyrus and orbifrontal structures, similar to those detected between AD and NC. It is curious that this phenomenon can be observed as early as subjects are still diagnosed as MCI. This result agrees with those of recent works studying functional changes in MCI through resting-state fMRI. It have been found that compared to healthy controls, MCI subjects exhibited a decrease functional activity in regions of the Default Mode Network (DMN) including left fusiform gyrus as well as an increased activity in other regions such as prefrontal cortex [113]. Summarizing, our findings revealed abnormal CMRgl covariations in AD and MCI that could reflect early stages of disorganization of the functional activity preceding the neuronal death for years. The CMRgl covariation patterns represent the high sensitivity of FDG-PET to explore pathophysiological processes at very early stages of neurodegenerative diseases such as Alzheimer and their possible application for predicting cognitive decline and response to disease-modifying therapy. Next section is aimed at discussing the changes of the CMRgl network attributes in AD and MCI where the disruption of underlying processes related to CMRgl covariations play a crucial role.

### CMRgl Network Attributes in AD and MCI

In our analysis the MCI and AD groups showed alterations in the CMRgl network’s attributes, such as ‘cluster index’ and ‘characteristic path length’ that indicate failures of equilibrium between network segregation and integration processes. These results are in compliance with previous works that use different neurophysiological and morphological variables to study brain networks [10]–[16]. They suggested that AD and MCI patients have disruptive neuronal integrity in large-scale structural and functional brain systems underlying high-level cognition. In addition, the abnormal segregated and integrative connectivity patterns support the notion of AD as a disconnection syndrome [9].

The topological properties of the CMRgl networks differ between AD and NC groups. The ‘characteristic path length’ in AD was statistically higher than the NC group. This alteration have been reported consistently in previous studies using other neuroimaging modalities [11], [12], [14], [16]. Our results suggest that this increment could be linked to the compensatory effects and the compromise of a set of regions in a common pathological process or shared vulnerability (occipital and frontal regions). These abnormal processes could influence the redistribution of principal CMRgl covariations (the highest correlation values) to these regions. Consequently, the CMRgl network in AD and MCI suffer a new reorganization where long distance connections play a less important role (for the network integration) inducing a longer characteristic path length. We found some hubs of the NC group not present in AD with disrupted glucose metabolism related to neurodegenerative processes (see Figure 13). Some of them were localized in parietal and temporal regions that link other cortical areas through long-range cortico-cortical connections, indispensable in the sensory integration [17]. The damaged connectivity in these areas could cause increments in the average characteristic path length.

The ‘clustering index’ attribute depicted the same behavior with higher values for AD and MCI patients as compared with NC. This is a measure of how similar CMRgl covariations are among brain structures neighborhoods (not reduced to a physical neighborhood concept). According to the small-world theoretical model (based on graph theory), this increase could be generated by the establishment of new densely connected local clusters which can generate an uncontrolled ‘flow of information’ through the entire network. This measure is related to the local efficiency of the ‘information flow’ of the networks and its abnormal performance could be the characteristic combination of disease-related changes (aberrant circuits) and compensatory mechanisms. The increased CMRgl covariation in frontal (compensatory mechanisms) and occipital (shared vulnerability effect) lobes in AD and MCI could explain the increasing clustering index in these pathologies. In line with this idea, we found that AD showed the largest number of areas with abnormal glucose metabolism (23 out of 90 representing the 25.5% out of total), followed by MCI (with 10 out of 90 that represents the 11.1% out of total). This set of regions with hypometabolism generates extended circuits with high CMRgl covariations that could increase the clustering index. This result may also explain why MCI occupies an intermediate position between NC and AD in terms of clustering index attribute.

It is noteworthy to point out, as previous authors have reported [11], [16], that the CMRgl networks in all groups showed small world architectures (see Figure S2). We found that the small world attribute (sigma) decreased in AD and MCI respect to NC. This network property is an indicator of the optimal balance between local specialization and global integration. Owing to the characteristic path length and clustering index attributes are higher in both pathological groups it could be thought that AD and MCI present an insult of the normal balance to a more regular network. This result is accordance with previous ones that use cortical thickness and volume as morphological variable [11], [16].

The increasing in ‘local efficiency’ was accompanied by a ‘global efficiency’ decline in AD and MCI. In terms of the graph theory these changes affect the network performance pointing to a higher ‘wiring cost’ for parallel ‘information transfer’ between anatomical regions. The weakening of the global efficiency is explained by larger characteristic path length present in AD and MCI. It is important to comment that the decline of the number of hub’s regions in AD as well as a non-uniformity distribution of them over the brain (see Figure 12) influence negatively the efficient communication over the CMRgl network that reduce the integration capacity and so the global efficiency. Moreover, we observed that almost all network attributes in MCI subjects exhibited an intermediate position between normal controls and AD. This fact provides evidences about MCI condition that clinicians believe often represents a transition phase to Alzheimer disease [113], [114].

In summary, the abnormalities found in the CMRgl network properties and the so-called cost-efficiency balance may be associated to the Alzheimer’s disease neurophatological processes such as the synaptic loss, reduced dendritic extent and local cell death.

### Hub Regions in AD and MCI Glucose Metabolism Networks

Hubs determine the pivotal regions that are crucial to an efficient communication in the complex networks [115]. Recently, several studies have suggested that brain regions that belong to the default mode network and areas with dense structural connectivity are most susceptible to the AD disease [5], [116]. The high baseline activity of hub regions may make them especially vulnerable to neurodegenerative changes (‘metabolism hypothesis’ of AD pathogenesis) [61], [116], [117]. In general, our results agreed the above studies in some points.

We found that most of hub regions in all groups were localized within the association cortex. The NC group present the highest number of these pivotal regions whereas in AD were found the lowest amount, almost half of the former. Some of these hubs in NC belong to the Default-mode Network (DMN) such as structures of the ventral medial prefrontal cortex (vMPFC) (supplementary motor cortex, left and right frontal superior cortex), inferior parietal lobe (IPL) (right inferior parietal gyrus) and medial temporal lobe structures [61], [117], [118]. The AD and MCI pathological groups present only one hub in the DMN located in the ventral and dorsal medial prefrontal cortex.

When we compared the spatial distribution of hubs between NC, MCI and AD groups, some aspects called our attention. First, the NC hubs were homogenously distributed over the occipital, limbic, parietal and frontal lobes. In the temporal lobe and nuclei only one hub was identified. On the contrary, a high number of hubs in AD were concentrated in occipital regions (8 out of 13 both brain hemispheres). Partially, it can be explained by the increased number of regions with glucose hypometabolism in this lobe that increments the CMRgl covariations by shared vulnerability processes. This mechanism changes the roles of the individual structures in the CMRgl network functioning. In addition it was observed that the CMRgl covariations over different brain lobes were unbalanced in AD and MCI (see Figure 7). In particular, the CMRgl covariations in occipital lobe is almost twice the values found in other brain lobes that indicates the presence of the strongest ‘connections’ in these regions. This fact could explain the reallocation of hubs to occipital lobe. Another aspect that could shed light on this phenomenon is that CMRgl covariations between occipital and other brain lobes were the highest for AD and MCI (see Figure S3), therefore it is reasonable to expect a high ‘traffic’ through this lobe that justify the presence of higher number of hubs respect to NC. As in other occasions MCI took an intermediate position between AD and NC.

Our findings are in line with previous papers that found precuneus, cuneus, thalamus, putamen, regions of the superior parietal cortex, and superior frontal gyrus as hub regions crucial for the well-functioning of brain networks [119], [120]. However, we observed in our study that all groups presented a weakening in the so-called ‘structure core’ that comprises a set of posterior medial and parietal cortical regions, establishing a densely interconnected and topologically central core (they constitute connector hubs that link all major structural modules) [119]. In the specific case of the NC group, this fact could be explained by normal aging effects that prevent some brain areas such as posterior cingulate cortex, isthmus of the cingulate, and hippocampus to be identified as hubs. These regions are well-known hubs of anatomical networks in young adult subjects [119].

It is noteworthy to mention that was not found a full coincidence between hubs regions found in our study and the ones reported in earlier studies [61]. We ascribed these dissimilarities to the existing differences among studies in terms of in neuroimaging modalities, technical procedures, sample characteristics and hub’s definition.

The smaller number of hubs localized in parietal, temporal and limbic regions in MCI and AD agrees with alterations in the ‘default-mode’ network (DMN) classified as memory related neuronal network and described through fMRI neuroimaging technique [121], [122]. It has been identified that MCI and AD subjects show early changes in cerebral glucose metabolism and blood flow in frontal, medial temporal, and posterior cingulate/precuneus areas of the brain [26], [95], [96], [123], suggesting that the DMN may be compromised. It is striking the fact that was not found any hub in limbic lobe in AD, which could be explained by pathological mechanisms (explained above) that reallocate hubs to the occipital lobe (see Figure S1 and Table S2).

Some hubs in NC presented glucose metabolism failures in AD and MCI. This fact evidences that AD targets some vital regions that could disturb the normal CMRgl networks functioning (Figure 13). It is worth noting that excepting the middle and superior occipital gyri (MOG and SOG) the NBC values of hubs in NC were systematically higher than in AD. Based on this finding we could hypothesize that the local glucose metabolism disruption is one of the mechanisms that change the role of the regions in the CMRgl network. In line with this idea we further found that glucose hypometabolism areas coincide with either increasing or decreasing of the NBC attribute (see Figure 11). Therefore the NBC changes related to glucose metabolism failures is a complex and multifactorial phenomenon that deserves a detailed study. Additionally, it should be noted that despite the NBC is a local attribute, this nodal property uses the global information of whole network. This makes the interpretation of the NBC-hypometabolism coupling even more difficult.

On the other hand, we found that NC subjects have more frontal hubs than AD and MCI groups. Given that frontal cortex structures undergo the largest age-related volumetric changes [124] this is an interesting finding in a group of elderly normal subjects. Nevertheless, a recent study of the topological patterns of the structural brain networks in younger and older cohorts showed no evidence of reduced ‘betweeness centrality’ in the prefrontal cortex between groups [80].The frontal cortex is one of the more flexible structures in the brain, and compensatory processes in the aged brain may largely reside in it [80], [125]. Moreover, this core of frontal hubs are in line with the ‘compensatory scaffolding’ hypothesis which posits that additional circuits are recruited by the aging brain to shore up the declining circuits whose functioning has become inefficient [125]. Creating scaffolds is suggested to be an active process throughout life, but in old age this may be accelerated as a compensatory mechanism.

In summary, the distribution of hubs of the CMRgl networks observed in all groups could be associated to the disease progression and aging related processes.

### Methodological Issues and Future Work

Several methodological issues need to be addressed in the future. First of all, it would be interesting to include NC, MCI and AD subjects from different databases to study the reproducibility of our findings and explore demographic effects. Second, partial correlation analysis should be used instead of the classical Pearson correlation adopted here. The partial correlation analysis could not be used in this study because the sample size was not large enough for a robust estimation of this measure (i.e. the number of structures in the AAL was higher than the number of subjects for each group). Third, we included only CMRgl networks analysis using FDG-PET. It would be very important in a future work to explore how brain network alterations in aging and AD are associated with alterations of anatomical/functional/metabolic variables, by combining structural MRI, fMRI, DWI and PET neuroimaging techniques. Fourth, it should be performed a study to disentangle the contributions of the negative and positive correlation values to the CMRgl networks. It is noteworthy to explore whether the negative correlations can lower the test-retest reliability of the network attributes. This issue has been previously reported in the literature for fMRI studies [126], [127]. Finally, here the MCI and AD related brain networks abnormalities were detected through a cross-sectional data; whereby results could be influenced by potential cohort effects. Future studies would be necessary to study the longitudinal network dynamics.

### Conclusions

In the present paper we have shown that combining graph theory and PET-FDG data allows studying the organizational properties of the glucose metabolism networks in the Alzheimer Disease, Mild Cognitive Impairment and in brain normal states. The possible relation between patterns of axonal (anatomical) and CMRgl covariations is a topic that should be addressed in future works. Our results highlight the importance of examining different physiological variables like glucose metabolism to understand normal/aberrant networks attributes in healthy and pathological brains. We consider our paper contributions shed light on the functional and anatomical connectomics of the Alzheimer and Mild Cognitive Impairment to reveal biological mechanisms underlying such diseases. This study is an attempt at addressing the complex association between glucose metabolism, CMRgl covariations and brain network attributes in AD and MCI based on graph theory.

## Supporting Information

Figure S1 **CMRgl covariations among regions with glucose hypometabolism found in AD.** Statistical differences between groups.
(DOC)

Figure S2 **Small world attribute (sigma).** Statistical differences between AD, MCI and NC groups.
(DOC)

Figure S3 **Mean CMRgl covariations among occipital lobe and other brain lobes.**: Frontal, Parietal, Temporal and Limbic. Statistical differences between groups. CMRgl covariations among brain lobes in AD, MCI and NC groups.
(DOC)

Table S1 **List of anatomical structures in the AAL atlas described in Tzourio-Mazoyer et al. (2002).**
(DOC)

Table S2 **Glucose metabolism (CMRgl) differences between groups.**
(DOC)

Table S3 **Statistical differences between CMRgl correlation matrices and its classification.** Only the main results are represented. For the full list of differences see Text S1.
(DOC)

Table S4 **Differences between groups in normalized betweenness centrality (NBC).**
(DOC)

Table S5 **Mean Normalized Betweenness Centrality (NBC) in NC, MCI and AD groups.** Hub regions.
(DOC)

Table S6 **List of regions with disrupted glucose metabolism in MCI and AD involved in abnormal CMRgl covariations as compared with NC.**
(DOC)

Text S1 **Full list of the statistical differences between CMRgl covariation matrices.**
(DOC)

## References

[1] American Psychiatric Association (1994) Diagnostic and statistical manual of mental disorders. Washington,DC.

[2] Villain N, Desgranges B, Viader F, de lS V, et al (2008) Relationships between hippocampal atrophy, white matter disruption, and gray matter hypometabolism in Alzheimer’s disease. J Neurosci 28: 6174–6181. 18550759 10.1523/JNEUROSCI.1392-08.2008 PMC2902815

[3] Englund E, Brun A, Alling C (1988) White matter changes in dementia of Alzheimer’s type. Biochemical and neuropathological correlates. Brain 111 (Pt 6): 1425–1439. 10.1093/brain/111.6.1425 3208064

[4] Kuczynski B, Targan E, Madison C, Weiner M, Zhang Y, et al (2010) White matter integrity and cortical metabolic associations in aging and dementia. Alzheimers Dement 6: 54–62. 20129319 10.1016/j.jalz.2009.04.1228 PMC2817977

[5] Raj A, Kuceyeski A, Weiner M (2012) A Network Diffusion Model of Disease Progression in Dementia. Neuron 73: 1204–1215. 22445347 10.1016/j.neuron.2011.12.040 PMC3623298

[6] Zhou J, Gennatas E, Kramer J, Miller B, Seeley W (2012) Predicting Regional Neurodegeneration from the Healthy Brain Functional Connectome. Neuron 73: 1216–1227. 22445348 10.1016/j.neuron.2012.03.004 PMC3361461

[7] Liu L, Drouet V, Wu JW, Witter MP, Small SA, et al (2012) Transsynaptic spread of tau pathology in vivo. PLoS One 7: e31302. 22312444 10.1371/journal.pone.0031302 PMC3270029

[8] Ronnback A, Sagelius H, Bergstedt KD, Naslund J, Westermark GT, et al (2012) Amyloid neuropathology in the single Arctic APP transgenic model affects interconnected brain regions. Neurobiol Aging 33: 831–839. 10.1016/j.neurobiolaging.2011.07.012 21880397

[9] Delbeuck X, Van der LM, Collette F (2003) Alzheimer’s disease as a disconnection syndrome? Neuropsychol Rev 13: 79–92. 12887040 10.1023/a:1023832305702

[10] de Haan W, Pijnenburg YA, Strijers RL, van der MY, van der Flier WM, et al (2009) Functional neural network analysis in frontotemporal dementia and Alzheimer’s disease using EEG and graph theory. BMC Neurosci 10: 101. 19698093 10.1186/1471-2202-10-101 PMC2736175

[11] He Y, Chen Z, Evans A (2008) Structural insights into aberrant topological patterns of large-scale cortical networks in Alzheimer’s Disease. Journal of Neuroscience 28: 4756–4766. 18448652 10.1523/JNEUROSCI.0141-08.2008 PMC6670444

[12] Lo CY, Wang PN, Chou KH, Wang J, He Y, et al (2010) Diffusion tensor tractography reveals abnormal topological organization in structural cortical networks in Alzheimer’s disease. J Neurosci 30: 16876–16885. 21159959 10.1523/JNEUROSCI.4136-10.2010 PMC6634928

[13] Sanz-Arigita EJ, Schoonheim MM, Damoiseaux JS, Rombouts SA, Maris E, et al (2010) Loss of ‘small-world’ networks in Alzheimer’s disease: graph analysis of FMRI resting-state functional connectivity. PLoS One 5: e13788. 21072180 10.1371/journal.pone.0013788 PMC2967467

[14] Stam CJ, de HW, Daffertshofer A, Jones BF, Manshanden I, et al (2009) Graph theoretical analysis of magnetoencephalographic functional connectivity in Alzheimer’s disease. Brain 132: 213–224. 18952674 10.1093/brain/awn262

[15] Supekar K, Menon V, Rubin D, Musen M, Greicius MD (2008) Network analysis of intrinsic functional brain connectivity in Alzheimer’s disease. PLoS Comput Biol 4: e1000100. 18584043 10.1371/journal.pcbi.1000100 PMC2435273

[16] Yao Z, Zhang Y, Lin L, Zhou Y, Xu C, et al (2010) Abnormal cortical networks in mild cognitive impairment and Alzheimer’s disease. PLoS Comput Biol 6: e1001006. 21124954 10.1371/journal.pcbi.1001006 PMC2987916

[17] Tijms BM, Wink AM, de HW, van der Flier WM, Stam CJ, et al.. (2013) Alzheimer’s disease: connecting findings from graph theoretical studies of brain networks. Neurobiol Aging. 10.1016/j.neurobiolaging.2013.02.020 23541878

[18] McKhann GM, Knopman DS, Chertkow H, Hyman BT, Jack CR, et al (2011) The diagnosis of dementia due to Alzheimer’s disease: Recommendations from the National Institute on Aging-Alzheimer’s Association workgroups on diagnostic guidelines for Alzheimer’s disease. Alzheimer’s & Dementia 7: 263–269. 10.1016/j.jalz.2011.03.005 PMC3312024 21514250

[19] Sperling RA, Aisen PS, Beckett LA, Bennett DA, Craft S, et al (2011) Toward defining the preclinical stages of Alzheimer’s disease: recommendations from the National Institute on Aging-Alzheimer’s Association workgroups on diagnostic guidelines for Alzheimer’s disease. Alzheimers Dement 7: 280–292. 21514248 10.1016/j.jalz.2011.03.003 PMC3220946

[20] Alexander GE, Chen K, Pietrini P, Rapoport SI, Reiman EM (2002) Longitudinal PET Evaluation of Cerebral Metabolic Decline in Dementia: A Potential Outcome Measure in Alzheimer’s Disease Treatment Studies. Am J Psychiatry 159: 738–745. 11986126 10.1176/appi.ajp.159.5.738

[21] Chen K, Langbaum JB, Fleisher AS, Ayutyanont N, Reschke C, et al (2010) Twelve-month metabolic declines in probable Alzheimer’s disease and amnestic mild cognitive impairment assessed using an empirically pre-defined statistical region-of-interest: findings from the Alzheimer’s Disease Neuroimaging Initiative. Neuroimage 51: 654–664. 20202480 10.1016/j.neuroimage.2010.02.064 PMC2856742

[22] Choo IH, Lee DY, Youn JC, Jhoo JH, Kim KW, et al (2007) Topographic patterns of brain functional impairment progression according to clinical severity staging in 116 Alzheimer disease patients: FDG-PET study. Alzheimer Dis Assoc Disord 21: 77–84. 17545731 10.1097/WAD.0b013e3180687418

[23] Langbaum JB, Chen K, Lee W, Reschke C, Bandy D, et al (2009) Categorical and correlational analyses of baseline fluorodeoxyglucose positron emission tomography images from the Alzheimer’s Disease Neuroimaging Initiative (ADNI). Neuroimage 45: 1107–1116. 19349228 10.1016/j.neuroimage.2008.12.072 PMC2886795

[24] Mosconi L, Mistur R, Switalski R, Tsui WH, Glodzik L, et al (2009) FDG-PET changes in brain glucose metabolism from normal cognition to pathologically verified Alzheimer’s disease. Eur J Nucl Med Mol Imaging 36: 811–822. 19142633 10.1007/s00259-008-1039-z PMC2774795

[25] Silverman DH, Small GW, Chang CY, Lu CS, Kung De Aburto MA, et al (2001) Positron emission tomography in evaluation of dementia: Regional brain metabolism and long-term outcome. JAMA 286: 2120–2127. 11694153 10.1001/jama.286.17.2120

[26] Herholz K, Salmon E, Perani D, Baron JC, Holthoff V, et al (2002) Discrimination between Alzheimer dementia and controls by automated analysis of multicenter FDG PET. Neuroimage 17: 302–316. 12482085 10.1006/nimg.2002.1208

[27] Reiman EM, Langbaum JB, Tariot PN (2010) Alzheimer’s prevention initiative: a proposal to evaluate presymptomatic treatments as quickly as possible. Biomark Med 4: 3–14. 20383319 10.2217/bmm.09.91 PMC2850446

[28] Reiman EM, Langbaum JB, Fleisher AS, Caselli RJ, Chen K, et al (2011) Alzheimer’s Prevention Initiative: a plan to accelerate the evaluation of presymptomatic treatments. J Alzheimers Dis 26 Suppl 3321–329. 21971471 10.3233/JAD-2011-0059 PMC3343739

[29] Metter EJ, Riege WH, Kameyama M, Kuhl DE, Phelps ME (1984) Cerebral metabolic relationships for selected brain regions in Alzheimer’s, Huntington’s, and Parkinson’s diseases. J Cereb Blood Flow Metab 4: 500–506. 6238975 10.1038/jcbfm.1984.74

[30] Horwitz B, Duara R, Rapoport SI (1984) Intercorrelations of glucose metabolic rates between brain regions: application to healthy males in a state of reduced sensory input. J Cereb Blood Flow Metab 4: 484–499. 6501442 10.1038/jcbfm.1984.73

[31] Horwitz B, Grady CL, Schlageter NL, Duara R, Rapoport SI (1987) Intercorrelations of regional cerebral glucose metabolic rates in Alzheimer’s disease. Brain Res 407: 294–306. 3494486 10.1016/0006-8993(87)91107-3

[32] Mosconi L, Perani D, Sorbi S, Herholz K, Nacmias B, et al (2004) MCI conversion to dementia and the APOE genotype: a prediction study with FDG-PET. Neurology 63: 2332–2340. 15623696 10.1212/01.wnl.0000147469.18313.3b

[33] Lee DS, Kang H, Kim H, Park H, Oh JS, et al (2008) Metabolic connectivity by interregional correlation analysis using statistical parametric mapping (SPM) and FDG brain PET; methodological development and patterns of metabolic connectivity in adults. Eur J Nucl Med Mol Imaging 35: 1681–1691. 18491089 10.1007/s00259-008-0808-z

[34] Damoiseaux JS, Rombouts SA, Barkhof F, Scheltens P, Stam CJ, et al (2006) Consistent resting-state networks across healthy subjects. Proc Natl Acad Sci U S A 103: 13848–13853. 16945915 10.1073/pnas.0601417103 PMC1564249

[35] Kerrouche N, Herholz K, Mielke R, Holthoff V, Baron JC (2006) 18FDG PET in vascular dementia: differentiation from Alzheimer’s disease using voxel-based multivariate analysis. J Cereb Blood Flow Metab 26: 1213–1221. 16525414 10.1038/sj.jcbfm.9600296

[36] Pagani M, Salmaso D, Rodriguez G, Nardo D, Nobili F (2009) Principal component analysis in mild and moderate Alzheimer’s disease–a novel approach to clinical diagnosis. Psychiatry Res 173: 8–14. 19443186 10.1016/j.pscychresns.2008.07.016

[37] Markiewicz PJ, Matthews JC, Declerck J, Herholz K (2011) Verification of predicted robustness and accuracy of multivariate analysis. Neuroimage 56: 1382–1385. 21338696 10.1016/j.neuroimage.2011.02.036 PMC3554787

[38] Illán IA, Górriz JM, Ramírez J, Salas-Gonzales D, López MM, et al (2011) 18F-FDG PET imaging analysis for computer aided Alzheimer’s diagnosis. Information Sciences 181: 903–916 10.1016/j.ins.2010.10.027.

[39] Toussaint PJ, Perlbarg V, Bellec P, Desarnaud S, Lacomblez L, et al (2012) Resting state FDG-PET functional connectivity as an early biomarker of Alzheimer’s disease using conjoint univariate and independent component analyses. Neuroimage 63: 936–946. 22510256 10.1016/j.neuroimage.2012.03.091

[40] Huang S, Li J, Sun L, Ye J, Fleisher A, et al (2010) Learning brain connectivity of Alzheimer’s disease by sparse inverse covariance estimation. Neuroimage 50: 935–949. 20079441 10.1016/j.neuroimage.2009.12.120 PMC3068623

[41] Zhang F, Zhang J, Zuo C, Guo W, Wang C (2011) Small-world properties of glucose metabolism based brain functional network. Chinese journal of medical instrumentation 35: 164–168. 21954571

[42] Mueller SG, Weiner MW, Thal LJ, Petersen RC, Jack CR, et al (2005) Ways toward an early diagnosis in Alzheimer’s disease: the Alzheimer’s Disease Neuroimaging Initiative (ADNI). Alzheimers Dement 1: 55–66. 17476317 10.1016/j.jalz.2005.06.003 PMC1864941

[43] Weiner MW, Veitch DP, Aisen PS, Beckett LA, Cairns NJ, et al (2012) The Alzheimer’s Disease Neuroimaging Initiative: a review of papers published since its inception. Alzheimers Dement 8: S1–68. 22047634 10.1016/j.jalz.2011.09.172 PMC3329969

[44] Jagust WJ, Bandy D, Chen K, Foster NL, Landau SM, et al (2010) The Alzheimer’s Disease Neuroimaging Initiative positron emission tomography core. Alzheimers Dement 6: 221–229. 20451870 10.1016/j.jalz.2010.03.003 PMC2920531

[45] Folstein MF, Folstein SE, McHugh PR (1975) “Mini-mental state”. A practical method for grading the cognitive state of patients for the clinician. J Psychiatr Res 12: 189–198. 1202204 10.1016/0022-3956(75)90026-6

[46] Morris JC (1993) The Clinical Dementia Rating (CDR): current version and scoring rules. Neurology 43: 2412–2414. 10.1212/wnl.43.11.2412-a 8232972

[47] Petersen RC, Doody R, Kurz A, Mohs RC, Morris JC, et al (2001) Current concepts in mild cognitive impairment. Arch Neurol 58: 1985–1992. 11735772 10.1001/archneur.58.12.1985

[48] McKhann G, Drachman D, Folstein M, Katzman R, Price D, et al (1984) Clinical diagnosis of Alzheimer’s disease: report of the NINCDS-ADRDA Work Group under the auspices of Department of Health and Human Services Task Force on Alzheimer’s Disease. Neurology 34: 939–944. 6610841 10.1212/wnl.34.7.939

[49] Gray KR, Wolz R, Heckemann RA, Aljabar P, Hammers A, et al (2012) Multiregion analysis of longitudinal FDG-PET for the classification of Alzheimer’s disease. Neuroimage 60: 221–229. 22236449 10.1016/j.neuroimage.2011.12.071 PMC3303084

[50] Landau SM, Harvey D, Madison CM, Koeppe RA, Reiman EM, et al (2011) Associations between cognitive, functional, and FDG-PET measures of decline in AD and MCI. Neurobiol Aging 32: 1207–1218. 19660834 10.1016/j.neurobiolaging.2009.07.002 PMC2891865

[51] Di X, Biswal B (2012) Metabolic Brain Covariant Networks as Revealed by FDG-PET with reference to resting-state fMRI networks. Brain Connect. 10.1089/brain.2012.0086 PMC3621675 23025619

[52] Rasmussen JM, Lakatos A, van Erp TG, Kruggel F, Keator DB, et al (2012) Empirical derivation of the reference region for computing diagnostic sensitive (1)(8)fluorodeoxyglucose ratios in Alzheimer’s disease based on the ADNI sample. Biochim Biophys Acta 1822: 457–466. 21958592 10.1016/j.bbadis.2011.09.008 PMC5849233

[53] Jovicich J, Czanner S, Greve D, Haley E, van der KA, et al (2006) Reliability in multi-site structural MRI studies: effects of gradient non-linearity correction on phantom and human data. Neuroimage 30: 436–443. 16300968 10.1016/j.neuroimage.2005.09.046

[54] Jack CR Jr, Bernstein MA, Fox NC, Thompson P, Alexander G, et al (2008) The Alzheimer’s Disease Neuroimaging Initiative (ADNI): MRI methods. J Magn Reson Imaging 27: 685–691. 18302232 10.1002/jmri.21049 PMC2544629

[55] Sled JG, Zijdenbos AP, Evans AC (1998) A nonparametric method for automatic correction of intensity nonuniformity in MRI data. IEEE Trans Med Imaging 17: 87–97. 9617910 10.1109/42.668698

[56] Alemán-Gómez Y, Melie-García L, Valdes-Hernández P (2006) IBASPM: Toolbox for automatic parcellation of brain structures.

[57] Moeller C, Vrenken H, Jiskoot L, Versteeg A, Barkhof F, et al. (2013) Different patterns of gray matter atrophy in early- and late-onset Alzheimer’s disease. Neurobiology of Aging. Available: http://dx.doi.org/10.1016/j.neurobiolaging.2013.02.013. 10.1016/j.neurobiolaging.2013.02.013 23561509

[58] Yakushev I, Landvogt C, Buchholz HG, Fellgiebel A, Hammers A, et al (2008) Choice of reference area in studies of Alzheimer’s disease using positron emission tomography with fluorodeoxyglucose-F18. Psychiatry Res 164: 143–153. 18930634 10.1016/j.pscychresns.2007.11.004

[59] Borghammer P, Aanerud J, Gjedde A (2009) Data-driven intensity normalization of PET group comparison studies is superior to global mean normalization. Neuroimage 46: 981–988. 19303935 10.1016/j.neuroimage.2009.03.021

[60] Yakushev I, Hammers A, Fellgiebel A, Schmidtmann I, Scheurich A, et al (2009) SPM-based count normalization provides excellent discrimination of mild Alzheimer’s disease and amnestic mild cognitive impairment from healthy aging. Neuroimage 44: 43–50. 18691659 10.1016/j.neuroimage.2008.07.015

[61] Buckner RL, Snyder AZ, Shannon BJ, LaRossa G, Sachs R, et al (2005) Molecular, structural, and functional characterization of Alzheimer’s disease: evidence for a relationship between default activity, amyloid, and memory. J Neurosci 25: 7709–7717. 16120771 10.1523/JNEUROSCI.2177-05.2005 PMC6725245

[62] Blesa R, Mohr E, Miletich RS, Hildebrand K, Sampson M, et al (1996) Cerebral metabolic changes in Alzheimer’s disease: neurobehavioral patterns. Dementia 7: 239–245. 8872413 10.1159/000106886

[63] Sanabria-Diaz G, Melie-Garcia L, Iturria-Medina Y, eman-Gomez Y, Hernandez-Gonzalez G, et al (2010) Surface area and cortical thickness descriptors reveal different attributes of the structural human brain networks. Neuroimage 50: 1497–1510. 20083210 10.1016/j.neuroimage.2010.01.028

[64] Tzourio-Mazoyer N, Landeau B, Papathanassiou D, Crivello F, Etard O, et al (2002) Automated anatomical labeling of activations in SPM using a macroscopic anatomical parcellation of the MNI MRI single-subject brain. Neuroimage 15: 273–289. 11771995 10.1006/nimg.2001.0978

[65] Melie-Garcia L, Sanabria-Diaz G, Sanchez-Catasus C (2013) Studying the topological organization of the cerebral blood flow fluctuations in resting state. Neuroimage 64: 173–184. 22975159 10.1016/j.neuroimage.2012.08.082

[66] Ginestet CE, Nichols TE, Bullmore ET, Simmons A (2011) Brain network analysis: separating cost from topology using cost-integration. PLoS One 6: e21570. 21829437 10.1371/journal.pone.0021570 PMC3145634

[67] Bassett DS, Nelson BG, Mueller BA, Camchong J, Lim KO (2012) Altered resting state complexity in schizophrenia. Neuroimage 59: 2196–2207. 22008374 10.1016/j.neuroimage.2011.10.002 PMC3254701

[68] He Y, Chen ZJ, Evans AC (2007) Small-world anatomical networks in the human brain revealed by cortical thickness from MRI. Cerebral Cortex 17: 2407–2419. 17204824 10.1093/cercor/bhl149

[69] Achard S, Bullmore E (2007) Efficiency and cost of economical brain functional networks. 10.1371/journal.pcbi.0030017 PMC1794324 17274684

[70] Sporns O (2011) The non-random brain: efficiency, economy, and complex dynamics. Front Comput Neurosci 5: 5. 21369354 10.3389/fncom.2011.00005 PMC3037776

[71] Boccaletti S, Latora V, Moreno Y, Chavez M, Hwang D-U (2006) Complex networks: Structure and dynamics. 175–308.

[72] Watts DJ, Strogatz SH (1998) Collective dynamics of small-world networks. 440–442. 10.1038/30918 9623998

[73] Watts DJ (1999) Small Worlds: The Dynamics of Networks between Order and Randomness.

[74] Maslov S, Sneppen K (2002) Specificity and stability in topology of protein networks. 10.1126/science.1065103 11988575

[75] Milo R, Shen-Orr S, Itzkovitz S, Kashan N, Chklovskii D, et al.. (2002) Network motifs: simple building blocks of complex networks. 824–827. 10.1126/science.298.5594.824 12399590

[76] Latora V, Marchiori M (2001) Efficient behavior of small-world networks. Phys Rev Lett 87: 198701. 11690461 10.1103/PhysRevLett.87.198701

[77] Freeman L (1977) A set of measures of centrality based upon betweenness.

[78] Achard S, Salvador R, Whitcher B, Suckling J, Bullmore E (2006) A resilient, lowfrequency, small-world human brain functional network with highly connected association cortical hubs. Journal of Neuroscience 26: 63–72. 16399673 10.1523/JNEUROSCI.3874-05.2006 PMC6674299

[79] Wu K, Taki Y, Sato K, Kinomura S, Goto R, et al.. (2011) Age-related changes in topological organization of structural brain networks in healthy individuals. Hum Brain Mapp. 10.1002/hbm.21232 PMC6870030 21391279

[80] Zhu W, Wen W, He Y, Xia A, Anstey KJ, et al.. (2010) Changing topological patterns in normal aging using large-scale structural networks. Neurobiol Aging. 10.1016/j.neurobiolaging.2010.06.022 20724031

[81] Cohen J, Cohen P (1983) Applied multiple regression/correlation analysis for the behavioral sciences. Hillsdale, NJ: Erlbaum.

[82] Genovese CR, Lazar NA, Nichols T (2002) Thresholding of statistical maps in functional neuroimaging using the false discovery rate. Neuroimage 15: 870–878. 11906227 10.1006/nimg.2001.1037

[83] He Y, Dagher A, Chen Z, Charil A, Zijdenbos A, et al (2009) Impaired small-world efficiency in structural cortical networks in multiple sclerosis associated with white matter lesion load. Brain 132: 3366–3379. 19439423 10.1093/brain/awp089 PMC2792366

[84] Mesulam MM (2000) Principles of Behavioral and Cognitive Neurology. Oxfor: OXFOR University Press.

[85] Morbelli S, Drzezga A, Perneczky R, Frisoni GB, Caroli A, et al (2012) Resting metabolic connectivity in prodromal Alzheimer’s disease. A European Alzheimer Disease Consortium (EADC) project. Neurobiol Aging 33: 2533–2550. 22365486 10.1016/j.neurobiolaging.2012.01.005

[86] Rocher AB, Chapon F, Blaizot X, Baron JC, Chavoix C (2003) Resting-state brain glucose utilization as measured by PET is directly related to regional synaptophysin levels: a study in baboons. Neuroimage 20: 1894–1898. 14642499 10.1016/j.neuroimage.2003.07.002

[87] Nobili F, Morbelli S (2010) 18F-FDG-PET as Biomarker for Early Alzheimer’s Disease. The Open Nuclear Medicine Journal 2: 46–52.

[88] Mosconi L, Tsui WH, Herholz K, Pupi A, Drzezga A, et al (2008) Multicenter standardized 18F-FDG PET diagnosis of mild cognitive impairment, Alzheimer’s disease, and other dementias. J Nucl Med 49: 390–398. 18287270 10.2967/jnumed.107.045385 PMC3703818

[89] Mosconi L (2005) Brain glucose metabolism in the early and specific diagnosis of Alzheimer’s disease. FDG-PET studies in MCI and AD. Eur J Nucl Med Mol Imaging 32: 486–510. 15747152 10.1007/s00259-005-1762-7

[90] Jagust W, Reed B, Mungas D, Ellis W, DeCarli C (2007) What does fluorodeoxyglucose PET imaging add to a clinical diagnosis of dementia? Neurology 69: 871–877. 17724289 10.1212/01.wnl.0000269790.05105.16

[91] Minoshima S, Giordani B, Berent S, Frey KA, Foster NL, et al (1997) Metabolic reduction in the posterior cingulate cortex in very early Alzheimer’s disease. Ann Neurol 42: 85–94. 9225689 10.1002/ana.410420114

[92] Eidelberg D (2009) Metabolic brain networks in neurodegenerative disorders: a functional imaging approach. Trends in Neurosciences 32: 548–557. 19765835 10.1016/j.tins.2009.06.003 PMC2782537

[93] Rapoport SI, Horwitz B, Grady CL, Haxby JV, DeCarli C, et al.. (1991) Abnormal brain glucose metabolism in Alzheimer’s disease, as measured by positron emission tomography. In: Fuel homeostasis and the nervous system. Springer. 231–248. 10.1007/978-1-4684-5931-9_18 1927686

[94] Pietrini P, Azari NP, Grady CL, Salerno JA, Gonzales-Aviles A, et al (1993) Pattern of cerebral metabolic interactions in a subject with isolated amnesia at risk for Alzheimer’s disease: a longitudinal evaluation. Dementia and Geriatric Cognitive Disorders 4: 94–101. 10.1159/000107349 8358518

[95] Greicius MD, Srivastava G, Reiss AL, Menon V (2004) Default-mode network activity distinguishes Alzheimer’s disease from healthy aging: evidence from functional MRI. Proc Natl Acad Sci U S A 101: 4637–4642. 15070770 10.1073/pnas.0308627101 PMC384799

[96] Rombouts SA, Barkhof F, Goekoop R, Stam CJ, Scheltens P (2005) Altered resting state networks in mild cognitive impairment and mild Alzheimer’s disease: an fMRI study. Hum Brain Mapp 26: 231–239. 15954139 10.1002/hbm.20160 PMC6871685

[97] Sorg C, Riedl V, Muhlau M, Calhoun VD, Eichele T, et al (2007) Selective changes of resting-state networks in individuals at risk for Alzheimer’s disease. Proc Natl Acad Sci U S A 104: 18760–18765. 18003904 10.1073/pnas.0708803104 PMC2141850

[98] Duffy CJ (2009) Visual Motion Processing in Aging and Alzheimer’s Disease. Annals of the New York Academy of Sciences 1170: 736–744. 19686221 10.1111/j.1749-6632.2009.04021.x

[99] Rizzo M, Anderson SW, Dawson J, Nawrot M (2000) Vision and cognition in Alzheimer’s disease. Neuropsychologia 38: 1157–1169. 10838150 10.1016/s0028-3932(00)00023-3

[100] Tetewsky SJ, Duffy CJ (1999) Visual loss and getting lost in Alzheimer’s disease. Neurology 52: 958. 10102412 10.1212/wnl.52.5.958

[101] Zalesky A, Fornito A, Egan GF, Pantelis C, Bullmore ET (2012) The relationship between regional and inter-regional functional connectivity deficits in schizophrenia. Hum Brain Mapp 33: 2535–2549. 21922601 10.1002/hbm.21379 PMC6870162

[102] Yu Q, Sui J, Liu J, Plis SM, Kiehl KA, et al (2013) Disrupted correlation between low frequency power and connectivity strength of resting state brain networks in schizophrenia. Schizophrenia Research 143: 165–171. 23182443 10.1016/j.schres.2012.11.001 PMC3540119

[103] Salmon E, Kerrouche N, Perani D, Lekeu F, Holthoff V, et al (2009) On the multivariate nature of brain metabolic impairment in Alzheimer’s disease. Neurobiol Aging 30: 186–197. 17651869 10.1016/j.neurobiolaging.2007.06.010

[104] Cabeza R, Nyberg L (2000) Imaging cognition II: An empirical review of 275 PET and fMRI studies. J Cogn Neurosci 12: 1–47. 10.1162/08989290051137585 10769304

[105] Stern Y (2006) Cognitive reserve and Alzheimer disease. Alzheimer Dis Assoc Disord 20: S69–S74. 16917199 10.1097/00002093-200607001-00010

[106] Grady CL, McIntosh AR, Beig S, Keightley ML, Burian H, et al (2003) Evidence from functional neuroimaging of a compensatory prefrontal network in Alzheimer’s disease. J Neurosci 23: 986–993. 12574428 10.1523/JNEUROSCI.23-03-00986.2003 PMC6741917

[107] Becker JT, Mintun MA, Aleva K, Wiseman MB, Nichols T, et al (1996) Compensatory reallocation of brain resources supporting verbal episodic memory in Alzheimer’s disease. Neurology 46: 692–700. 8618669 10.1212/wnl.46.3.692

[108] Wang K, Liang M, Wang L, Tian L, Zhang X, et al (2007) Altered functional connectivity in early Alzheimer’s disease: a resting-state fMRI study. Hum Brain Mapp 28: 967–978. 17133390 10.1002/hbm.20324 PMC6871392

[109] Agosta F, Pievani M, Geroldi C, Copetti M, Frisoni GB, et al (2012) Resting state fMRI in Alzheimer’s disease: beyond the default mode network. Neurobiol Aging 33: 1564–1578. 21813210 10.1016/j.neurobiolaging.2011.06.007

[110] Barbas H (1988) Anatomic organization of basoventral and mediodorsal visual recipient prefrontal regions in the rhesus monkey. J Comp Neurol 276: 313–342. 3192766 10.1002/cne.902760302

[111] Dolan RJ, Fletcher P, Morris J, Kapur N, Deakin JF, et al (1996) Neural activation during covert processing of positive emotional facial expressions. Neuroimage 4: 194–200. 9345509 10.1006/nimg.1996.0070

[112] Geday J, Gjedde A, Boldsen AS, Kupers R (2003) Emotional valence modulates activity in the posterior fusiform gyrus and inferior medial prefrontal cortex in social perception. Neuroimage 18: 675–684. 12667845 10.1016/s1053-8119(02)00038-1

[113] Qi Z, Wu X, Wang Z, Zhang N, Dong H, et al (2010) Impairment and compensation coexist in amnestic MCI default mode network. Neuroimage 50: 48–55. 20006713 10.1016/j.neuroimage.2009.12.025

[114] Gili T, Cercignani M, Serra L, Perri R, Giove F, et al (2011) Regional brain atrophy and functional disconnection across Alzheimer’s disease evolution. J Neurol Neurosurg Psychiatry 82: 58–66. 20639384 10.1136/jnnp.2009.199935

[115] Bassett DS, Bullmore E (2006) Small-world brain networks. Neuroscientist 12: 512–523. 17079517 10.1177/1073858406293182

[116] Buckner RL, Sepulcre J, Talukdar T, Krienen FM, Liu H, et al (2009) Cortical hubs revealed by intrinsic functional connectivity: mapping, assessment of stability, and relation to Alzheimer’s disease. J Neurosci 29: 1860–1873. 19211893 10.1523/JNEUROSCI.5062-08.2009 PMC2750039

[117] Buckner RL, Andrews-Hanna JR, Schacter DL (2008) The brain’s default network: anatomy, function, and relevance to disease. Ann N Y Acad Sci 1124: 1–38. 18400922 10.1196/annals.1440.011

[118] Acosta-Cabronero J, Williams GB, Pengas G, Nestor PJ (2010) Absolute diffusivities define the landscape of white matter degeneration in Alzheimer’s disease. Brain 133: 529–539. 19914928 10.1093/brain/awp257

[119] Hagmann P, Cammoun L, Gigandet X, Meuli R, Honey CJ, et al (2008) Mapping the structural core of human cerebral cortex. PLoS Biol 6: e159. 18597554 10.1371/journal.pbio.0060159 PMC2443193

[120] van den Heuvel MP, Sporns O (2011) Rich-club organization of the human connectome. J Neurosci 31: 15775–15786. 22049421 10.1523/JNEUROSCI.3539-11.2011 PMC6623027

[121] Greicius MD, Krasnow B, Reiss AL, Menon V (2003) Functional connectivity in the resting brain: a network analysis of the default mode hypothesis. Proc Natl Acad Sci U S A 100: 253–258. 12506194 10.1073/pnas.0135058100 PMC140943

[122] Raichle ME, Snyder AZ (2007) A default mode of brain function: a brief history of an evolving idea. Neuroimage 37: 1083–1090. 17719799 10.1016/j.neuroimage.2007.02.041

[123] Kogure D, Matsuda H, Ohnishi T, Asada T, Uno M, et al (2000) Longitudinal evaluation of early Alzheimer’s disease using brain perfusion SPECT. J Nucl Med 41: 1155–1162. 10914904

[124] Resnick SM, Pham DL, Kraut MA, Zonderman AB, Davatzikos C (2003) Longitudinal magnetic resonance imaging studies of older adults: a shrinking brain. J Neurosci 23: 3295–3301. 12716936 10.1523/JNEUROSCI.23-08-03295.2003 PMC6742337

[125] Park DC, Reuter-Lorenz P (2009) The adaptive brain: aging and neurocognitive scaffolding. Annu Rev Psychol 60: 173–196. 19035823 10.1146/annurev.psych.59.103006.093656 PMC3359129

[126] Wang JH, Zuo XN, Gohel S, Milham MP, Biswal BB, et al (2011) Graph theoretical analysis of functional brain networks: test-retest evaluation on short- and long-term resting-state functional MRI data. PLoS One 6: e21976. 21818285 10.1371/journal.pone.0021976 PMC3139595

[127] Schwarz AJ, McGonigle J (2011) Negative edges and soft thresholding in complex network analysis of resting state functional connectivity data. Neuroimage 55: 1132–1146. 21194570 10.1016/j.neuroimage.2010.12.047

