# Supplementary material for: Glucose Metabolism during Resting State Reveals Abnormal Brain Networks Organization in the Alzheimer’s Disease and Mild Cognitive Impairment
Source: PLoS One. 2013 Jul 23;8(7):e68860. doi: 10.1371/journal.pone.0068860 (PMC3720883; doi:10.1371/journal.pone.0068860)

**Supporting Information Figure S1**

It is shown the differences among groups (NC, MCI and AD) in CMRgl covariations between regions with hypometabolism found in AD group. The list of the 23 regions with abnormal glucose metabolism in AD can be found in Supporting Information Tables S2, Table S2.1 NC vs. AD. The nonparametric Kruskal-Wallis test was used to study differences. All post hoc tests were significant (NC vs. AD; NC vs. MCI and MCI vs. AD). Details are found in tables below.


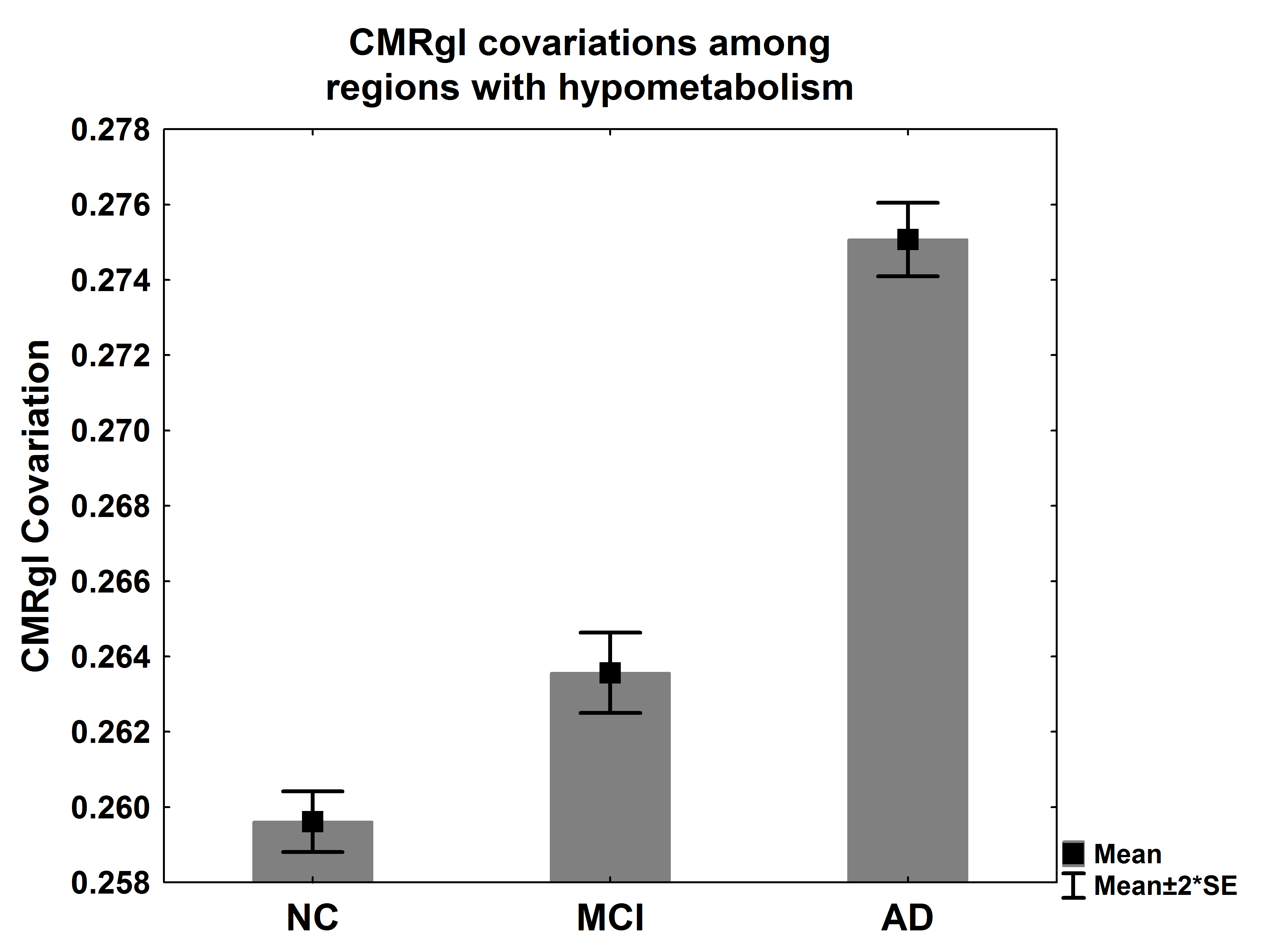

Supplement: Figure S1 — CMRgl covariations among regions with glucose hypometabolism found in AD. Statistical differences between groups. (DOC) [file pone.0068860.s001.doc]
