# Supplementary material for: Glucose Metabolism during Resting State Reveals Abnormal Brain Networks Organization in the Alzheimer’s Disease and Mild Cognitive Impairment
Source: PLoS One. 2013 Jul 23;8(7):e68860. doi: 10.1371/journal.pone.0068860 (PMC3720883; doi:10.1371/journal.pone.0068860)
Supplement: Figure S2 — Small world attribute (sigma). Statistical differences between AD, MCI and NC groups. (DOC) [file pone.0068860.s002.doc]

**Supporting Information Figure S2**

It is shown the small world attribute for all groups (NC, MCI and AD). This network property is an indicator of the optimal balance between local specialization and global integration. The nonparametric Kruskal-Wallis test was used a to study differences among groups. Details are found in tables below. The MCI and AD groups were not different (p=0.58). These groups as compared with NC were different (p<10^-20^).


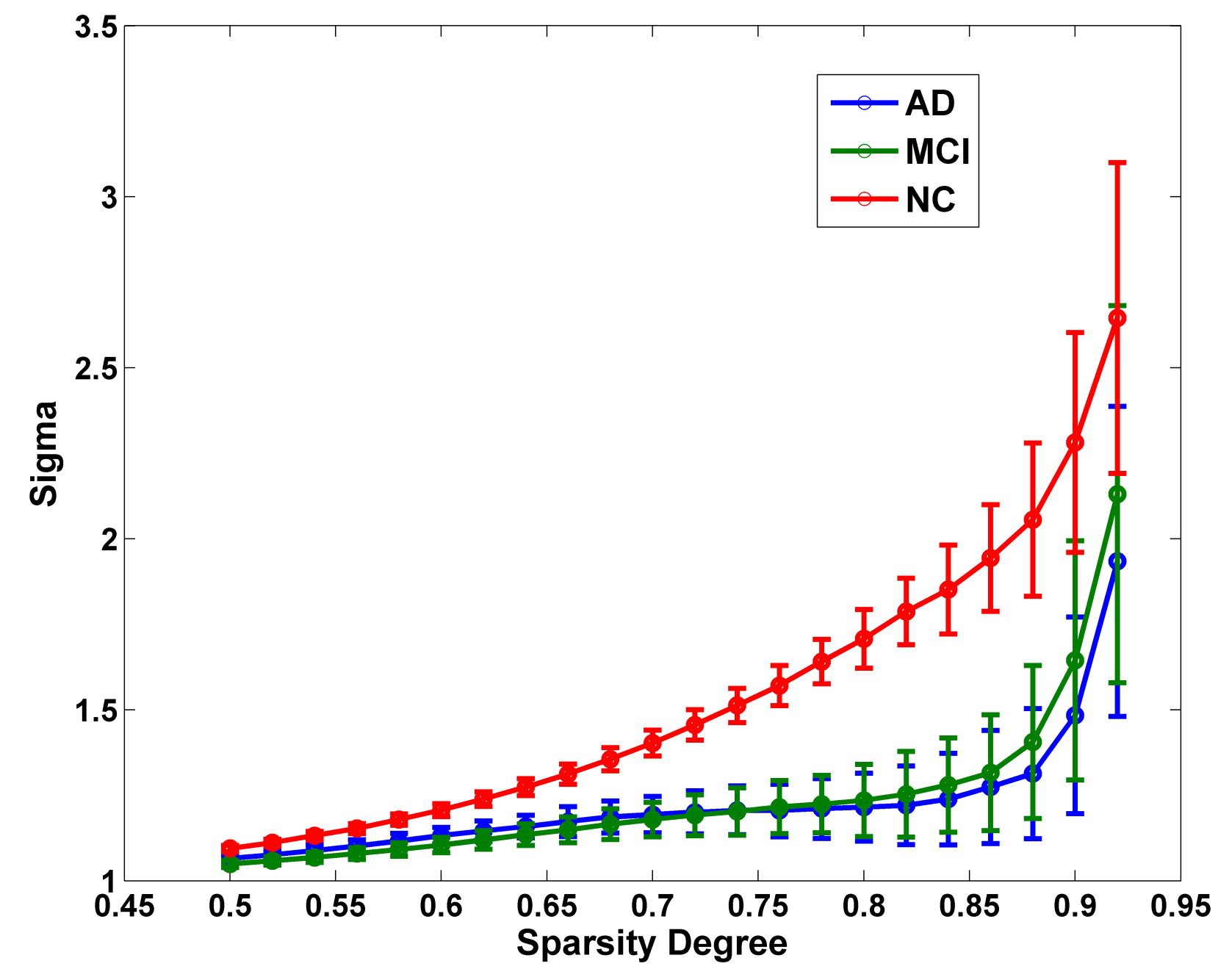


**Statistical Test Tables**
