# Supplementary material for: Glucose Metabolism during Resting State Reveals Abnormal Brain Networks Organization in the Alzheimer’s Disease and Mild Cognitive Impairment
Source: PLoS One. 2013 Jul 23;8(7):e68860. doi: 10.1371/journal.pone.0068860 (PMC3720883; doi:10.1371/journal.pone.0068860)
Supplement: Figure S3 — Mean CMRgl covariations among occipital lobe and other brain lobes.: Frontal, Parietal, Temporal and Limbic. Statistical differences between groups. CMRgl covariations among brain lobes in AD, MCI and NC groups. (DOC) [file pone.0068860.s003.doc]

**Supporting Information Figure S3**

**Figure S3.1.** It is depicted the mean CMRgl covariation of the occipital lobe with the other brain lobes: Frontal, Parietal, Temporal and Limbic.

**
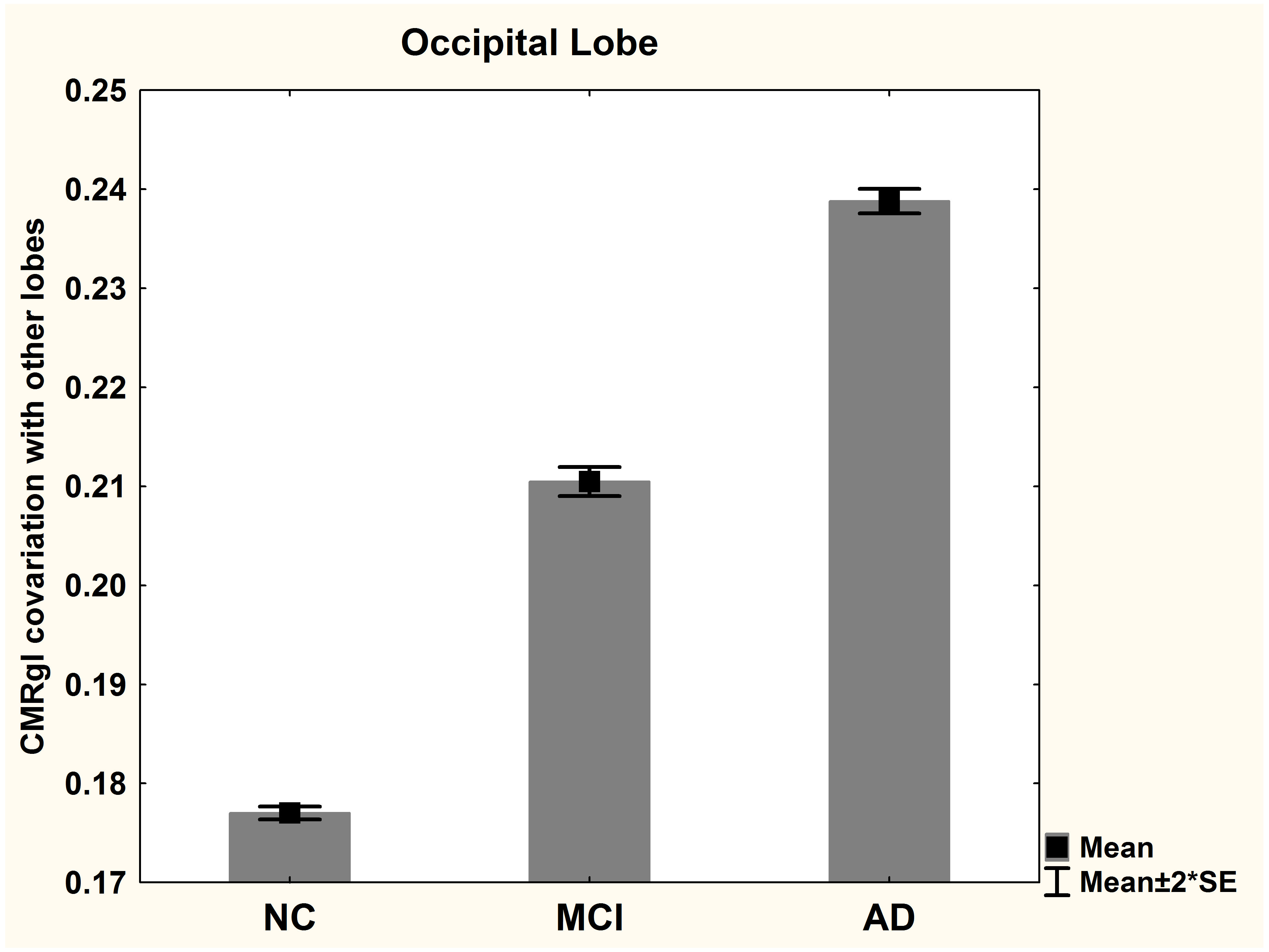
**

Here below are shown the results of the Kruskal-Wallis test to evaluate differences among NC, MCI and AD groups.

**Figure S3.2.** CMRgl covariations among brain lobes in AD group

**
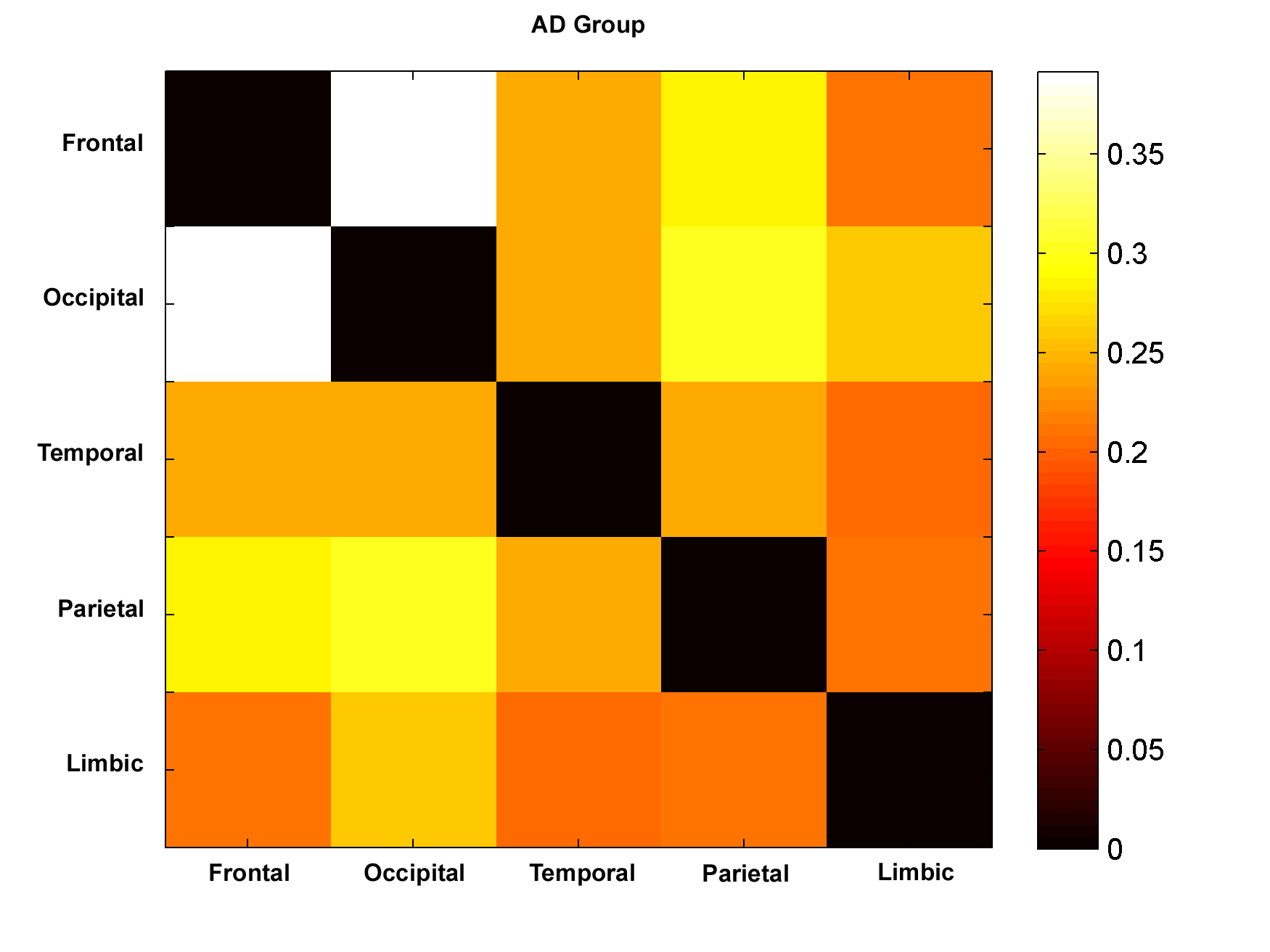
**

**Figure S3.3.** CMRgl covariations among brain lobes in MCI group

**
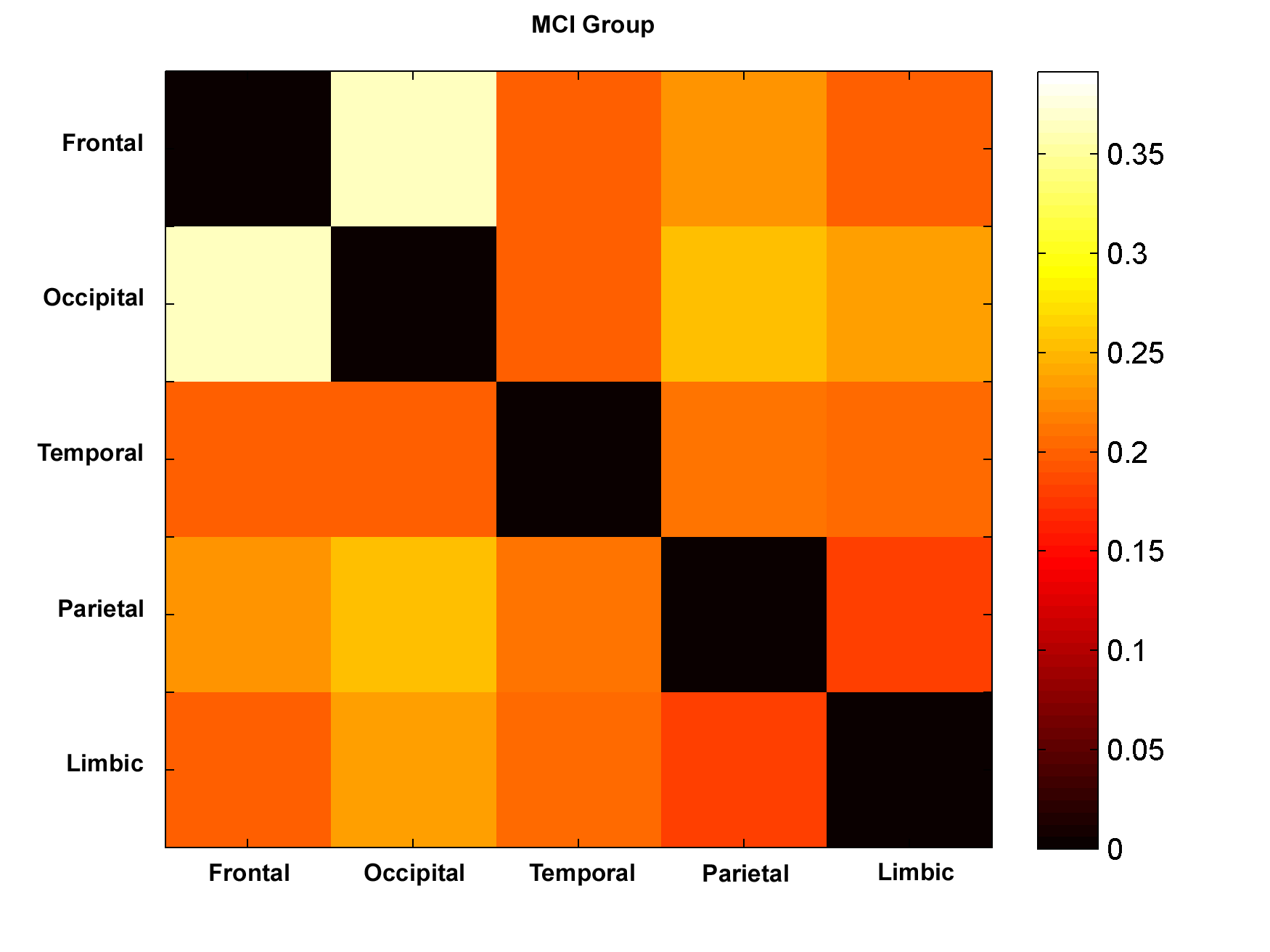
**

**Figure S3.4.** CMRgl covariations among brain lobes in NC group

**
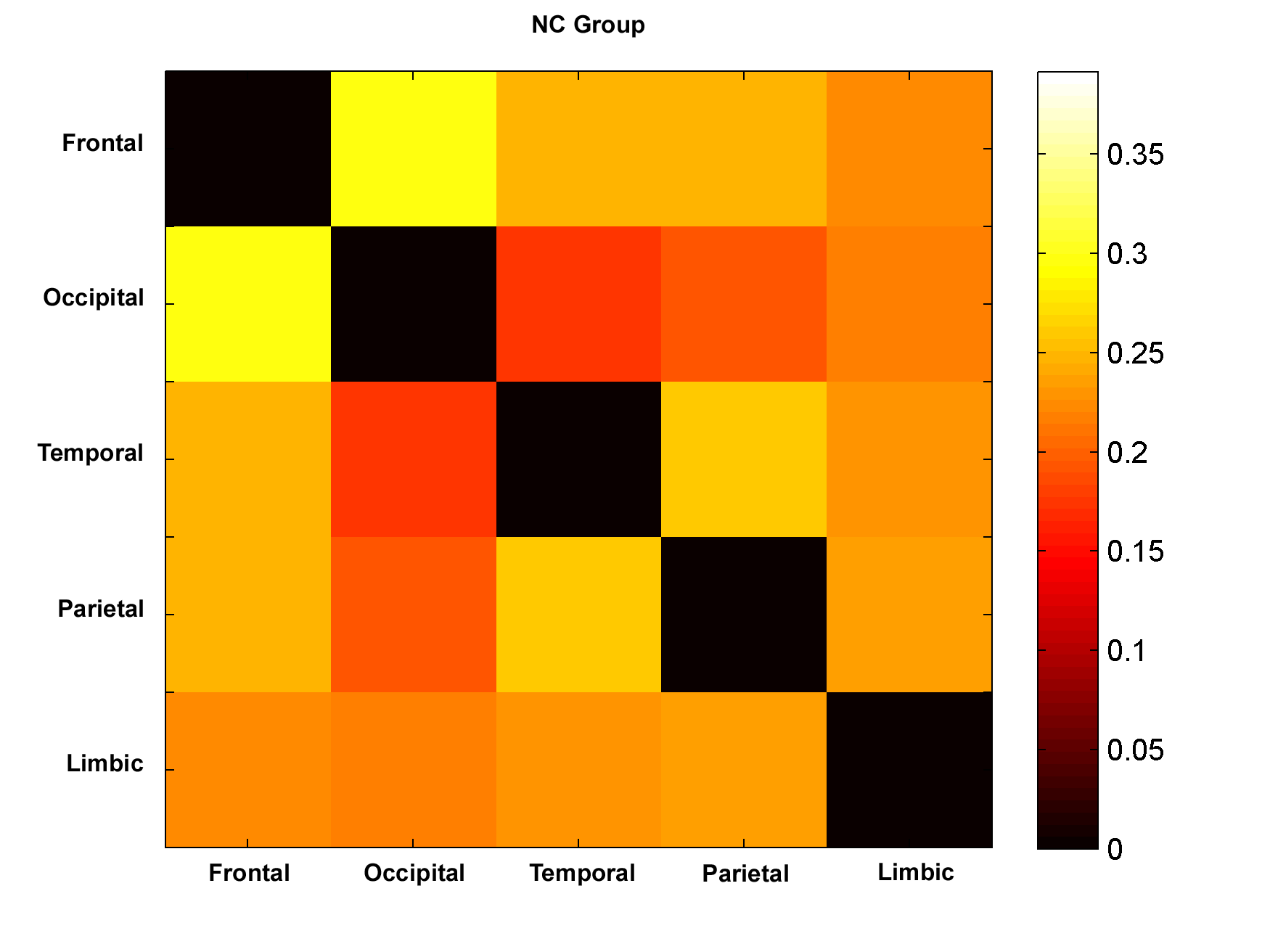
**
