# Supplementary material for: Glucose Metabolism during Resting State Reveals Abnormal Brain Networks Organization in the Alzheimer’s Disease and Mild Cognitive Impairment
Source: PLoS One. 2013 Jul 23;8(7):e68860. doi: 10.1371/journal.pone.0068860 (PMC3720883; doi:10.1371/journal.pone.0068860)
Supplement: Table S1 — List of anatomical structures in the AAL atlas described in Tzourio-Mazoyer et al. (2002). (DOC) [file pone.0068860.s004.doc]

**Supporting Information Table S1**

List of anatomical structures in the AAL atlas described in Tzourio-Mazoyer et al. (2002). Only the first 90 structures were used, cerebellum was not included. The full structure names as well as the respectively short names used in some figures and the body of the main manuscript are specified. The lobe to which the structure belongs is also included.

Legend:

- Brain Lobe: L: Limbic, I: insula, PM: parietal medial surface, PL: parietal lateral surface, OM: occipital medial surface, OL: occipital lateral surface, FL: frontal lateral surface, FM: frontal medial surface, FO: frontal orbital surface, TL: temporal lateral surface, C: central region, N: Sub cortical gray nuclei.

References:

- Tzourio-Mazoyer, N., Papathanassiou, D., Crivello, F., Etard, O., Delcroix, N., Mayozer, B., Joliot, M., (2002). Automated Anatomical Labeling of Activations in SPM Using a Macroscopic Anatomical Parcellation of the MNI MRI Single-Subject Brain. Neuroimage 15, pp. 273-289.

| **Code** | **Structure Full name** | **Structure**  **Short Name** | **Brain**  **Lobe** |
| --- | --- | --- | --- |
| 1 | Right Precentral gyrus | PreCG.R | C |
| 2 | Right Superior frontal gyrus, dorsolateral | SFGdor.R | FL |
| 3 | Right Superior frontal gyrus, orbital part | ORBsup.R | FO |
| 4 | Right Middle frontal gyrus | MFG.R | FL |
| 5 | Right Middle frontal gyrus, orbital part | ORBmid.R | FO |
| 6 | Right Inferior frontal gyrus, opercular part | IFGoperc.R | FL |
| 7 | Right Inferior frontal gyrus, triangular part | IFGtriang.R | FL |
| 8 | Right Inferior frontal gyrus, orbital part | ORBinf.R | FO |
| 9 | Right Rolandic operculum | ROL.R | C |
| 10 | Right Supplementary motor area | SMA.R | FM |
| 11 | Right Olfactory cortex | OLF.R | FO |
| 12 | Right Superior frontal gyrus, medial | SFGmed.R | FM |
| 13 | Right Superior frontal gyrus, medial orbital | ORBsupmed.R | FO |
| 14 | Right Gyrus rectus | REC.R | FO |
| 15 | Right Insula | INS.R | I |
| 16 | Right Anterior cingulate and paracingulate gyri | ACG.R | L |
| 17 | Right Median cingulate and paracingulate gyri | DCG.R | L |
| 18 | Right Posterior cingulate gyrus | PCG.R | L |
| 19 | Right Hippocampus | HIP.R | L |
| 20 | Right Parahippocampal gyrus | PHG.R | L |
| 21 | Right Amygdala | AMYG.R | N |
| 22 | Right Calcarine fissure and surrounding cortex | CAL.R | OM |
| 23 | Right Cuneus | CUN.R | OM |
| 24 | Right Lingual gyrus | LING.R | OM |
| 25 | Right Superior occipital gyrus | SOG.R | OL |
| 26 | Right Middle occipital gyrus | MOG.R | OL |
| 27 | Right Inferior occipital gyrus | IOG.R | OL |
| 28 | Right Fusiform gyrus | FFG.R | OM |
| 29 | Right Postcentral gyrus | PoCG.R | C |
| 30 | Right Superior parietal gyrus | SPG.R | PL |
| 31 | Right Inferior parietal, but supramarginal and angular gyri | IPL.R | PL |
| 32 | Right Supramarginal gyrus | SMG.R | PL |
| 33 | Right Angular gyrus | ANG.R | PL |
| 34 | Right Precuneus | PCUN.R | PM |
| 35 | Right Paracentral lobule | PCL.R | FM |
| 36 | Right Caudate nucleus | CAU.R | N |
| 37 | Right Lenticular nucleus, putamen | PUT.R | N |
| 38 | Right Lenticular nucleus, pallidum | PAL.R | N |
| 39 | Right Thalamus | THA.R | N |
| 40 | Right Heschl gyrus | HES.R | TL |
| 41 | Right Superior temporal gyrus | STG.R | TL |
| 42 | Right Temporal pole: superior temporal gyrus | TPOsup.R | L |
| 43 | Right Middle temporal gyrus | MTG.R | TL |
| 44 | Right Temporal pole: middle temporal gyrus | TPOmid.R | L |
| 45 | Right Inferior temporal gyrus | ITG.R | TL |
| 1 | Left Precentral gyrus | PreCG.L | C |
| 2 | Left Superior frontal gyrus, dorsolateral | SFGdor.L | FL |
| 3 | Left Superior frontal gyrus, orbital part | ORBsup.L | FO |
| 4 | Left Middle frontal gyrus | MFG.L | FL |
| 5 | Left Middle frontal gyrus, orbital part | ORBmid.L | FO |
| 6 | Left Inferior frontal gyrus, opercular part | IFGoperc.L | FL |
| 7 | Left Inferior frontal gyrus, triangular part | IFGtriang.L | FL |
| 8 | Left Inferior frontal gyrus, orbital part | ORBinf.L | FO |
| 9 | Left Rolandic operculum | ROL.L | C |
| 10 | Left Supplementary motor area | SMA.L | FM |
| 11 | Left Olfactory cortex | OLF.L | FO |
| 12 | Left Superior frontal gyrus, medial | SFGmed.L | FM |
| 13 | Left Superior frontal gyrus, medial orbital | ORBsupmed.L | FO |
| 14 | Left Gyrus rectus | REC.L | FO |
| 15 | Left Insula | INS.L | I |
| 16 | Left Anterior cingulate and paracingulate gyri | ACG.L | L |
| 17 | Left Median cingulate and paracingulate gyri | DCG.L | L |
| 18 | Left Posterior cingulate gyrus | PCG.L | L |
| 19 | Left Hippocampus | HIP.L | L |
| 20 | Left Parahippocampal gyrus | PHG.L | L |
| 21 | Left Amygdala | AMYG.L | N |
| 22 | Left Calcarine fissure and surrounding cortex | CAL.L | OM |
| 23 | Left Cuneus | CUN.L | OM |
| 24 | Left Lingual gyrus | LING.L | OM |
| 25 | Left Superior occipital gyrus | SOG.L | OL |
| 26 | Left Middle occipital gyrus | MOG.L | OL |
| 27 | Left Inferior occipital gyrus | IOG.L | OL |
| 28 | Left Fusiform gyrus | FFG.L | OM |
| 29 | Left Postcentral gyrus | PoCG.L | C |
| 30 | Left Superior parietal gyrus | SPG.L | PL |
| 31 | Left Inferior parietal, but supramarginal and angular gyri | IPL.L | PL |
| 32 | Left Supramarginal gyrus | SMG.L | PL |
| 33 | Left Angular gyrus | ANG.L | PL |
| 34 | Left Precuneus | PCUN.L | PM |
| 35 | Left Paracentral lobule | PCL.L | FM |
| 36 | Left Caudate nucleus | CAU.L | N |
| 37 | Left Lenticular nucleus, putamen | PUT.L | N |
| 38 | Left Lenticular nucleus, pallidum | PAL.L | N |
| 39 | Left Thalamus | THA.L | N |
| 40 | Left Heschl gyrus | HES.L | TL |
| 41 | Left Superior temporal gyrus | STG.L | TL |
| 42 | Left Temporal pole: superior temporal gyrus | TPOsup.L | L |
| 43 | Left Middle temporal gyrus | MTG.L | TL |
| 44 | Left Temporal pole: middle temporal gyrus | TPOmid.L | L |
| 45 | Left Inferior temporal gyrus | ITG.L | TL |
