# Supplementary material for: Glucose Metabolism during Resting State Reveals Abnormal Brain Networks Organization in the Alzheimer’s Disease and Mild Cognitive Impairment
Source: PLoS One. 2013 Jul 23;8(7):e68860. doi: 10.1371/journal.pone.0068860 (PMC3720883; doi:10.1371/journal.pone.0068860)
Supplement: Table S2 — Glucose metabolism (CMRgl) differences between groups. (DOC) [file pone.0068860.s005.doc]

**Supporting Information Tables S2**

Glucose metabolism (CMRgl) differences between groups.

*Note*: Please see Supporting Information Table S1 for the full name of the anatomical structures.

**Table S2.1.** **NC vs. AD**

| **Structure** | **NC (Residual CMRgl)** | **AD (Residual CMRgl)** | **T(129)** | **p-value** | **Brain Lobe** |
| --- | --- | --- | --- | --- | --- |
| **PCG.R** | 0.45 | 0.26 | 8.31 | 3.17·10^-13^ | Limbic |
| **PHG.R** | 0.27 | 0.22 | 6.28 | 1.16·10^-8^ | Limbic |
| **IPL.R** | 0.32 | 0.23 | 4.19 | 9.76·10^-5^ | Parietal |
| **ANG.R** | 0.34 | 0.19 | 6.80 | 8.85·10^-10^ | Parietal |
| **PCUN.R** | 0.31 | 0.20 | 5.02 | 3.65·10^-6^ | Parietal |
| **MTG.R** | 0.32 | 0.23 | 5.44 | 5.90·10^-7^ | Temporal |
| **TPOmid.R** | 0.30 | 0.26 | 3.80 | 4.01·10^-4^ | Limbic |
| **ITG.R** | 0.27 | 0.20 | 4.95 | 4.81·10^-6^ | Temporal |
| **PCG.L** | 0.46 | 0.27 | 8.31 | 3.05·10^-13^ | Limbic |
| **HIP.L** | 0.27 | 0.23 | 4.54 | 2.58·10^-5^ | Limbic |
| **PHG.L** | 0.30 | 0.24 | 8.01 | 1.62·10^-12^ | Limbic |
| **CUN.L** | 0.40 | 0.30 | 4.54 | 2.58·10^-5^ | Occipital |
| **SOG.L** | 0.35 | 0.27 | 3.74 | 4.98·10^-4^ | Occipital |
| **MOG.L** | 0.34 | 0.25 | 3.96 | 2.31·10^-4^ | Occipital |
| **IOG.L** | 0.33 | 0.27 | 3.39 | 1.52·10^-3^ | Occipital |
| **FFG.L** | 0.29 | 0.24 | 3.96 | 2.27·10^-4^ | Occipital |
| **IPL.L** | 0.34 | 0.25 | 4.27 | 7.48·10^-5^ | Parietal |
| **ANG.L** | 0.34 | 0.17 | 6.92 | 4.75·10^-10^ | Parietal |
| **PCUN.L** | 0.30 | 0.21 | 4.00 | 1.96·10^-4^ | Parietal |
| **PAL.L** | 0.37 | 0.31 | 4.35 | 5.49·10^-5^ | Nucleus |
| **MTG.L** | 0.30 | 0.21 | 4.97 | 4.55·10^-6^ | Temporal |
| **TPOmid.L** | 0.30 | 0.25 | 4.18 | 1.03·10^-4^ | Limbic |
| **ITG.L** | 0.29 | 0.20 | 6.17 | 1.97·10^-8^ | Temporal |

**Table S2.2. NC vs. MCI**

| **Structure** | **NC (Residual CMRgl)** | **MCI (Residual CMRgl)** | **T(117)** | **p-value** | **Brain Lobe** |
| --- | --- | --- | --- | --- | --- |
| **PCG.R** | 0.46 | 0.38 | 3.63 | 7.39·10^-4^ | Limbic |
| **PHG.R** | 0.26 | 0.23 | 4.96 | 5.10·10^-6^ | Limbic |
| **FFG.R** | 0.32 | 0.28 | 3.73 | 5.24·10^-4^ | Occipital |
| **ANG.R** | 0.40 | 0.32 | 3.47 | 1.22·10^-3^ | Parietal |
| **MTG.R** | 0.37 | 0.31 | 3.56 | 9.08·10^-4^ | Temporal |
| **TPOmid.R** | 0.32 | 0.28 | 3.43 | 1.39·10^-3^ | Limbic |
| **ITG.R** | 0.33 | 0.27 | 3.93 | 2.61·10^-4^ | Temporal |
| **PCG.L** | 0.49 | 0.39 | 4.16 | 1.16·10^-4^ | Limbic |
| **PHG.L** | 0.30 | 0.26 | 4.61 | 2.10·10^-5^ | Limbic |
| **ITG.L** | 0.35 | 0.31 | 3.47 | 1.22·10^-3^ | Temporal |

**Table S2.3. MCI vs. AD**

| **Structure** | **MCI (Residual CMRgl)** | **AD (Residual CMRgl)** | **T(117)** | **p-value** | **Brain Lobe** |
| --- | --- | --- | --- | --- | --- |
| **PCG.R** | 0.34 | 0.23 | 4.52 | 3.0·10^-5^ | Limbic |
| **ANG.R** | 0.24 | 0.15 | 3.69 | 6.08·10^-4^ | Parietal |
| **PCG.L** | 0.33 | 0.23 | 4.21 | 9.60·10^-5^ | Limbic |
| **PHG.L** | 0.26 | 0.23 | 4.05 | 1.73·10^-4^ | Limbic |
| **ANG.L** | 0.25 | 0.16 | 4.16 | 1.18·10^-4^ | Parietal |
| **ITG.L** | 0.25 | 0.20 | 3.35 | 1.79·10^-3^ | Temporal |
