# Supplementary material for: Glucose Metabolism during Resting State Reveals Abnormal Brain Networks Organization in the Alzheimer’s Disease and Mild Cognitive Impairment
Source: PLoS One. 2013 Jul 23;8(7):e68860. doi: 10.1371/journal.pone.0068860 (PMC3720883; doi:10.1371/journal.pone.0068860)
Supplement: Table S3 — Statistical differences between CMRgl correlation matrices and its classification. Only the main results are represented. For the full list of differences see Text S1. (DOC) [file pone.0068860.s006.doc]

**Supporting Information Table S3**

Principal Statistical differences between CMRgl correlation matrices and its classification. Only the main results are presented. For the full list of differences see Supporting Information Text S1.

**Table S3.1**. Significant differences between AD and MCI groups in CMRgl connectivity matrices.

| **MCI vs. AD** | | | | |
| --- | --- | --- | --- | --- |
| **Structure 1** | **Structure 2** | **Z** | **MCI** | **AD** |
| **Decreased positive correlations in AD** |  |  |  |  |
| *---------------------------------------------* | *---------------------------------------------* | ------- | ------- | ------- |
| **Increased positive correlations in AD** |  |  |  |  |
| Pallidum L | Olfactory cortex L | -5.58 | -0.04 | 0.74 |
| Superior frontal gyrus, medial L | Inferior frontal gyrus, orbital part L | -4.05 | 0.27 | 0.76 |
| Superior frontal gyrus, medial L | Superior frontal gyrus, orbital part L | -3.90 | 0.36 | 0.79 |
| **Decreased negative correlations in AD** |  |  |  |  |
| **--------------------------------------------** | *---------------------------------------------* | ------- | ------- | ------- |
| **Increased negative correlations in AD** |  |  |  |  |
| Inferior frontal gyrus, opercular part L | Supramarginal gyrus R | 4.10 | 0.04 | -0.60 |

Z denotes the z test value.

**Table S3.2**. Significant differences between NC and MCI groups in CMRgl connectivity matrices.

| **NC vs. MCI** | | | | |
| --- | --- | --- | --- | --- |
| **Structure 1** | **Structure 2** | **Z** | **NC** | **MCI** |
| **Decreased positive correlations in MCI** |  |  |  |  |
| Middle temporal gyrus L | Temporal pole L Superior temporal | 3.79 | 0.62 | 0.04 |
| Temporal pole L | gyrus L | 3.79 | 0.69 | 0.16 |
| **Increased positive correlations in MCI** |  |  |  |  |
| Middle temporal gyrus L | Postcentral gyrus R | -4.76 | -0.67 | 0.04 |
| Middle temporal gyrus L | Superior parietal gyrus R | -4.31 | -0.63 | 0.03 |
| Inferior temporal gyrus L | Postcentral gyrus R | -4.10 | -0.63 | 0.01 |
| Fusiform gyrus L | Postcentral gyrus R | -3.66 | -0.38 | 0.25 |
| Fusiform gyrus L | Cuneus L | -3.63 | -0.01 | 0.57 |
| Fusiform gyrus L | Superior Parietal gyrus R | -3.56 | -0.27 | 0.34 |
| Inferior frontal gyrus, triangular part L | Inferior frontal gyrus, opercular part R | -3.54 | -0.18 | 0.43 |
| **Decreased negative correlations in MCI** |  |  |  |  |
| Middle temporal gyrus L | Precentral gyrus right | -4.35 | -0.75 | -0.16 |
| **Increased negative correlations in MCI** |  |  |  |  |
| Fusiform gyrus L | Inferior frontal gyrus, orbital part L | 3.71 | 0.36 | -0.28 |
| Fusiform gyrus L | Superior frontal gyrus, orbital part R | 3.78 | 0.25 | -0.40 |
| Fusiform gyrus L | Superior frontal gyrus, orbital part L | 3.81 | 0.31 | -0.34 |
| Fusiform gyrus L | Middle frontal gyrus, orbital part R | 3.82 | 0.21 | -0.44 |
| Fusiform gyrus L | Middle frontal gyrus, orbital part L | 3.90 | 0.25 | -0.42 |
| Fusiform gyrus L | Inferior frontal gyrus, orbital part R | 3.90 | 0.23 | -0.43 |
| Fusiform gyrus L | Superior frontal gyrus, medial orbital R | 3.96 | 0.26 | -0.42 |

Z denotes the z test value.

**Table S3.3**. Significant differences between NC and AD groups in CMRgl connectivity matrices.

| **NC vs. AD** | | | | |
| --- | --- | --- | --- | --- |
| **Structure 1** | **Structure 2** | **Z** | **NC** | **AD** |
| **Decreased positive correlations in AD** |  |  |  |  |
| Superior frontal gyrus L | Middle frontal gyrus R | 4.01 | 0.73 | 0.23 |
| Paracentral lobule L | Superior parietal gyrus R | 3.90 | 0.65 | 0.10 |
| Postcentral gyrus L | Precentral gyrus R | 3.74 | 0.67 | 0.15 |
| Precentral gyrus L | Superior frontal gyrus R | 3.58 | 0.56 | 0.01 |
| Superior parietal gyrus L | Paracentral lobule R | 3.57 | 0.65 | 0.16 |
| **Increased positive correlations in AD** |  |  |  |  |
| Inferior frontal gyrus, orbital part L | Superior frontal gyrus L | -5.97 | -0.20 | 0.69 |
| Superior frontal gyrus, medial L | Inferior frontal gyrus, orbital part L | -5.77 | -0.01 | 0.76 |
| Inferior frontal gyrus, orbital part L | Middle frontal gyrus L | -5.43 | 0.09 | 0.78 |
| Superior parietal gyrus L | Inferior occipital gyrus L | -5.40 | -0.10 | 0.69 |
| Middle temporal gyrus L | Superior parietal gyrus L | -5.37 | -0.39 | 0.48 |
| **Decreased negative correlations in AD** |  |  |  |  |
| Middle temporal gyrus L | Paracentral lobule L | -3.49 | -0.63 | -0.14 |
| Middle temporal gyrus L | Superior frontal gyrus L | -3.45 | -0.58 | -0.06 |
| Angular gyrus R | Amygdala R | -3.28 | -0.56 | -0.06 |
| Middle temporal gyrus L | Paracentral lobule R | -3.22 | -0.64 | -0.20 |
| Middle temporal gyrus L | Postcentral gyrus R | -3.22 | -0.67 | -0.24 |
| **Increased negative correlations in AD** |  |  |  |  |
| Superior frontal gyrus, orbital part L | Fusiform gyrus R | 4.51 | 0.09 | -0.60 |
| Superior parietal gyrus L | Middle frontal gyrus R | 4.16 | 0.20 | -0.48 |
| Gyrus rectus L | Fusiform gyrus R | 4.12 | 0.13 | -0.53 |
| Fusiform gyrus L | Gyrus rectus R | 4.12 | 0.30 | -0.39 |
| Fusiform gyrus L | Inferior frontal gyrus, orbital part R | 4.11 | 0.23 | -0.44 |

Only the five highest differences in each classification were shown whereby 20 pairs of structures are depicted. The full list of 183 differences can be found in Text S1. Z denotes the z test value.
