# Supplementary material for: Glucose Metabolism during Resting State Reveals Abnormal Brain Networks Organization in the Alzheimer’s Disease and Mild Cognitive Impairment
Source: PLoS One. 2013 Jul 23;8(7):e68860. doi: 10.1371/journal.pone.0068860 (PMC3720883; doi:10.1371/journal.pone.0068860)
Supplement: Table S4 — Differences between groups in normalized betweenness centrality (NBC). (DOC) [file pone.0068860.s007.doc]

**Supporting Information Table S4**

Differences between groups in normalized betweenness centrality (NBC). In bold are represented the structures where first group has higher NBC than the second group. The hub regions are represented in red.

**Table S4.1 NC vs. AD**

| **Structure** | **NC**  **NBC value** | **AD**  **NBC value** | **Brain Lobe** |
| --- | --- | --- | --- |
| ORBmid.R | 0.38 | 0.79 | Frontal |
| **SMA.R** | **1.68** | **0.38** | Frontal |
| ACG.R | 0.25 | 0.95 | Limbic |
| **AMYG.R** | **1.56** | **0.72** | Nucleus |
| CUN.R | 0.93 | 1.53 | Occipital |
| MOG.R | 1.51 | 2.36 | Occipital |
| **SPG.R** | **1.79** | **1.37** | Parietal |
| SMG.R | 0.72 | 1.42 | Parietal |
| **PCL.R** | **1.25** | **0.19** | Frontal |
| **TPOsup.R** | **2.70** | **0.52** | Limbic |
| **TPOmid.R** | **1.85** | **0.78** | Limbic |
| ITG.R | 0.52 | 1.30 | Temporal |
| IFGoperc.L | 0.54 | 1.22 | Frontal |
| ROL.L | 0.41 | 0.99 | Central |
| **INS.L** | **2.20** | **1.27** | Insula |
| ACG.L | 0.19 | 1.26 | Limbic |
| **HIP.L** | **0.98** | **0.66** | Limbic |
| **PHG.L** | **1.17** | **0.25** | Limbic |
| IOG.L | 1.08 | 2.54 | Occipital |
| SPG.L | 0.82 | 1.89 | Parietal |
| IPL.L | 0.35 | 1.15 | Parietal |
| **PCL.L** | **1.09** | **0.23** | Frontal |
| PAL.L | 0.54 | 0.86 | Nucleus |
| **THA.L** | **0.29** | **0.09** | Nucleus |
| **TPOsup.L** | **1.57** | **1.02** | Limbic |

**Table S4.2. NC vs. MCI**

| **Structure** | **NC**  **NBC value** | **MCI**  **NBC value** | **Brain Lobe** |
| --- | --- | --- | --- |
| **PreCG.R** | **1.29** | **0.37** | Central |
| **ORBsup.R** | **1.05** | **0.53** | Frontal |
| IFGoperc.R | 0.44 | 0.66 | Frontal |
| **ORBinf.R** | **1.50** | **0.48** | Frontal |
| **OLF.R** | **1.04** | **0.41** | Frontal |
| ORBsupmed.R | 0.81 | 1.27 | Frontal |
| INS.R | 0.99 | 2.14 | Insula |
| ACG.R | 0.25 | 1.85 | Limbic |
| **AMYG.R** | **1.56** | **0.54** | Nucleus |
| IOG.R | 1.17 | 2.09 | Occipital |
| **PoCG.R** | **1.36** | **0.64** | Central |
| **IPL.R** | **1.56** | **0.86** | Parietal |
| **PCUN.R** | **2.17** | **1.18** | Parietal |
| **PCL.R** | **1.25** | **0.59** | Frontal |
| CAU.R | 0.09 | 1.31 | Nucleus |
| PUT.R | 0.83 | 1.92 | Nucleus |
| PAL.R | 0.65 | 1.06 | Nucleus |
| **TPOsup.R** | **2.70** | **0.62** | Limbic |
| **TPOmid.R** | **1.85** | **0.41** | Limbic |
| ITG.R | 0.52 | 1.27 | Temporal |
| **SFGdor.L** | **1.60** | **0.81** | Frontal |
| **REC.L** | **0.80** | **0.45** | Frontal |
| ACG.L | 0.19 | 1.49 | Limbic |
| **AMYG.L** | **0.76** | **0.29** | Nucleus |
| IOG.L | 1.07 | 2.18 | Occipital |
| IPL.L | 0.35 | 0.89 | Parietal |
| **PCUN.L** | **1.81** | **1.08** | Parietal |
| **PCL.L** | **1.09** | **0.57** | Frontal |
| **STG.L** | **1.11** | **0.78** | Temporal |
| MTG.L | 1.57 | 2.02 | Temporal |
| **TPOmid.L** | **1.35** | **0.48** | Limbic |

**Table S4.3. MCI vs. AD**

| **Structure** | **MCI**  **NBC value** | **AD**  **NBC value** | **Brain Lobe** |
| --- | --- | --- | --- |
| ORBsup.R | 0.53 | 1.02 | Frontal |
| OLF.R | 0.41 | 0.83 | Frontal |
| **ORBsupmed.R** | **1.27** | **0.64** | Frontal |
| **INS.R** | **2.14** | **0.71** | Insula |
| **CAL.R** | **0.61** | **0.37** | Occipital |
| SMG.R | 0.73 | 1.42 | Parietal |
| **CAU.R** | **1.31** | **0.60** | Nucleus |
| **MTG.R** | **2.22** | **1.12** | Temporal |
| SFGdor.L | 0.81 | 1.45 | Frontal |
| ORBsup.L | 0.48 | 0.93 | Frontal |
| OLF.L | 0.65 | 1.28 | Frontal |
| ORBsupmed.L | 0.46 | 0.82 | Frontal |
| REC.L | 0.46 | 0.72 | Frontal |
| **INS.L** | **1.98** | **1.27** | Insula |
| **HIP.L** | **1.49** | **0.66** | Limbic |
| **PHG.L** | **1.01** | **0.25** | Limbic |
| MOG.L | 1.58 | 2.83 | Occipital |
| SMG.L | 0.43 | 1.36 | Parietal |
| **PCL.L** | **0.57** | **0.23** | Frontal |
| **HES.L** | **0.45** | **0.17** | Temporal |
| **MTG.L** | **2.02** | **1.56** | Temporal |
