# Supplementary material for: Glucose Metabolism during Resting State Reveals Abnormal Brain Networks Organization in the Alzheimer’s Disease and Mild Cognitive Impairment
Source: PLoS One. 2013 Jul 23;8(7):e68860. doi: 10.1371/journal.pone.0068860 (PMC3720883; doi:10.1371/journal.pone.0068860)
Supplement: Table S5 — Mean Normalized Betweenness Centrality (NBC) in NC, MCI and AD groups. Hub regions. (DOC) [file pone.0068860.s008.doc]

**Supporting Information Table S5**

Mean Normalized Betweenness Centrality (NBC) in NC, MCI and AD groups. In bold are represented the brain regions found as hubs.

Legend:

- Mean Betweenness Centrality (NBC): mean NBC over the sparsity degree range. The sparsity degree range comprised 19 values.
- Region Name: Name of anatomical regions, L and R represent the left and right hemispheres respectively.
- Brain Lobe: L: Limbic, I: insula, PM: parietal medial surface, PL: parietal lateral surface, OM: occipital medial surface, OL: occipital lateral surface, FL: frontal lateral surface, FM: frontal medial surface, FO: frontal orbital surface, TL: temporal lateral surface, C: central region, N: Sub cortical gray nuclei.
- Number of times as hub: This is the number of times out the total number of sparsity degrees (19 in our case) where the structure reached the hub condition in the CMRgl network (NBC>1.5).

**NC group**

| Mean Betweenness Centrality (NBC) | Region Name | Number of times as hub | Brain  Lobe |
| --- | --- | --- | --- |
| **2.7058** | **Temporal Pole Sup R** | **19** | **L** |
| **2.2041** | **Insula L** | **19** | **I** |
| **2.1736** | **Precuneus R** | **19** | **PM** |
| **2.054** | **Cuneus L** | **17** | **OM** |
| **1.8557** | **Temporal Pole Mid R** | **16** | **L** |
| **1.8131** | **Precuneus L** | **19** | **PM** |
| **1.7922** | **Parietal Sup R** | **14** | **PL** |
| **1.6956** | **Frontal Mid L** | **19** | **FL** |
| **1.693** | **Occipital Sup R** | **14** | **OL** |
| **1.6775** | **Supp Motor Area R** | **19** | **FM** |
| **1.5975** | **Frontal Sup L** | **11** | **FL** |
| **1.5871** | **Occipital Sup L** | **10** | **OL** |
| **1.5721** | **Temporal Pole Sup L** | **17** | **L** |
| **1.5717** | **Temporal Mid L** | **8** | **TL** |
| **1.5713** | **Frontal Sup R** | **10** | **FL** |
| **1.5642** | **Parietal Inf R** | **11** | **PL** |
| **1.5566** | **Amygdala R** | **9** | **N** |
| **1.5253** | **Occipital Mid L** | **8** | **OL** |
| **1.5137** | **Occipital Mid R** | **6** | **OL** |
| 1.499 | Frontal Inf Orb R | 11 | FO |
| 1.4615 | Temporal Mid R | 9 | TL |
| 1.4492 | Supp Motor Area L | 0 | FM |
| 1.4194 | Frontal Mid R | 7 | FL |
| 1.3827 | Angular L | 2 | PL |
| 1.3671 | Fusiform L | 0 | OM |
| 1.3616 | Postcentral R | 0 | C |
| 1.3551 | Temporal Pole Mid L | 6 | L |
| 1.3395 | Fusiform R | 5 | OM |
| 1.2869 | Precentral R | 0 | C |
| 1.258 | Angular R | 0 | PL |
| 1.2515 | Paracentral Lobule R | 0 | FM |
| 1.1865 | Temporal Sup R | 3 | TL |
| 1.1722 | Occipital Inf R | 0 | OL |
| 1.1711 | ParaHippocampal L | 0 | L |
| 1.1112 | Temporal Sup L | 2 | TL |
| 1.0921 | Paracentral Lobule L | 0 | FM |
| 1.0756 | Occipital Inf L | 0 | OL |
| 1.0501 | Frontal Sup Orb R | 0 | FO |
| 1.0426 | Olfactory R | 0 | FO |
| 1.0259 | Putamen L | 0 | N |
| 1.0117 | Frontal Inf Orb L | 0 | FO |
| 0.9875 | Insula R | 0 | I |
| 0.9831 | Hippocampus L | 0 | L |
| 0.9335 | Cuneus R | 0 | OM |
| 0.9011 | Temporal Inf L | 0 | TL |
| 0.8767 | Cingulum Mid R | 0 | L |
| 0.8687 | Rolandic Oper R | 0 | C |
| 0.8618 | Caudate L | 0 | N |
| 0.8284 | Putamen R | 0 | N |
| 0.8225 | Parietal Sup L | 0 | PL |
| 0.8123 | Frontal Med Orb R | 0 | FO |
| 0.7994 | Rectus L | 0 | FO |
| 0.7737 | Cingulum Mid L | 0 | L |
| 0.7637 | Amygdala L | 0 | N |
| 0.7617 | Frontal Med Orb L | 0 | FO |
| 0.7591 | Calcarine L | 0 | OM |
| 0.7492 | Postcentral L | 0 | C |
| 0.7162 | SupraMarginal R | 0 | PL |
| 0.7110 | Frontal Sup Medial L | 0 | FM |
| 0.7013 | Frontal Inf Tri L | 0 | FL |
| 0.6857 | Olfactory L | 0 | FO |
| 0.6511 | Pallidum R | 0 | N |
| 0.6491 | Calcarine R | 0 | OM |
| 0.6463 | Heschl R | 0 | TL |
| 0.6442 | SupraMarginal L | 0 | PL |
| 0.6415 | Frontal Inf Tri R | 0 | FL |
| 0.5750 | Rectus R | 0 | FO |
| 0.5423 | Frontal Inf Oper L | 0 | FL |
| 0.5389 | Frontal Mid Orb L | 0 | FO |
| 0.5375 | Pallidum L | 0 | N |
| 0.5360 | Precentral L | 0 | C |
| 0.5323 | Frontal Sup Orb L | 0 | FO |
| 0.5196 | Temporal Inf R | 0 | TL |
| 0.4733 | Lingual R | 0 | OM |
| 0.4594 | Heschl L | 0 | TL |
| 0.4436 | Frontal Inf Oper R | 0 | FL |
| 0.4337 | Frontal Sup Medial R | 0 | FM |
| 0.4119 | Rolandic Oper L | 0 | C |
| 0.4076 | Lingual L | 0 | OM |
| 0.3963 | ParaHippocampal R | 0 | L |
| 0.3839 | Frontal Mid Orb R | 0 | FO |
| 0.3590 | Thalamus R | 0 | N |
| 0.3492 | Parietal Inf L | 0 | PL |
| 0.3174 | Cingulum Post L | 0 | L |
| 0.2880 | Thalamus L | 0 | N |
| 0.2538 | Cingulum Ant R | 0 | L |
| 0.2030 | Cingulum Post R | 0 | L |
| 0.1927 | Cingulum Ant L | 0 | L |
| 0.1275 | Hippocampus R | 0 | L |
| 0.0927 | Caudate R | 0 | N |

**MCI group**

| Mean Betweenness Centrality (NBC) | Region Name | Number of times as hub | Brain  Lobe |
| --- | --- | --- | --- |
| **2.2249** | **Temporal Mid R** | **19** | **TL** |
| **2.1843** | **Occipital Inf L** | **19** | **OL** |
| **2.1398** | **Insula R** | **19** | **I** |
| **2.1278** | **Occipital Mid R** | **19** | **OL** |
| **2.0955** | **Occipital Inf R** | **19** | **OL** |
| **2.0191** | **Temporal Mid L** | **19** | **TL** |
| **1.9846** | **Insula L** | **19** | **I** |
| **1.9769** | **Fusiform L** | **17** | **OM** |
| **1.9243** | **Putamen R** | **19** | **N** |
| **1.8648** | **Cingulum Mid R** | **15** | **L** |
| **1.8476** | **Cingulum Ant R** | **19** | **L** |
| **1.6915** | **Parietal Sup R** | **14** | **PL** |
| **1.6707** | **Occipital Sup L** | **11** | **OL** |
| **1.661** | **Temporal Pole Sup L** | **15** | **L** |
| **1.6298** | **Temporal Sup R** | **15** | **TL** |
| **1.5951** | **Occipital Sup R** | **10** | **OL** |
| **1.5895** | **Parietal Sup L** | **11** | **PL** |
| **1.5795** | **Occipital Mid L** | **18** | **OL** |
| **1.5025** | **Frontal Mid Orb R** | **10** | **FO** |
| 1.4938 | Cingulum Ant L | 5 | L |
| 1.492 | Hippocampus L | 8 | L |
| 1.4545 | Cuneus L | 7 | OM |
| 1.4064 | Supp Motor Area R | 7 | FM |
| 1.3135 | Supp Motor Area L | 0 | FM |
| 1.3077 | Caudate R | 2 | N |
| 1.2713 | Frontal Med Orb R | 6 | FO |
| 1.2697 | Temporal Inf R | 1 | TL |
| 1.2472 | Frontal Mid R | 3 | FL |
| 1.1847 | Fusiform R | 0 | OM |
| 1.1827 | Precuneus R | 0 | PM |
| 1.1616 | Frontal Sup R | 2 | FL |
| 1.1381 | Cuneus R | 2 | OM |
| 1.0869 | Temporal Inf L | 5 | TL |
| 1.0851 | Precuneus L | 0 | PM |
| 1.0806 | Frontal Mid Orb L | 0 | FO |
| 1.0649 | Pallidum R | 2 | N |
| 1.013 | ParaHippocampal L | 4 | L |
| 1.0032 | Angular R | 0 | PL |
| 0.9655 | Frontal Inf Oper L | 0 | FL |
| 0.9293 | Frontal Mid L | 0 | FL |
| 0.9224 | Frontal Sup Medial R | 0 | FM |
| 0.8935 | Frontal Sup Medial L | 0 | FM |
| 0.8902 | Parietal Inf L | 0 | PL |
| 0.8861 | Frontal Inf Tri L | 0 | FL |
| 0.8711 | Cingulum Mid L | 0 | L |
| 0.8560 | Parietal Inf R | 0 | PL |
| 0.8168 | Putamen L | 0 | N |
| 0.8090 | Frontal Sup L | 0 | FL |
| 0.7907 | Lingual R | 0 | OM |
| 0.783 | Rolandic Oper R | 0 | C |
| 0.7779 | Thalamus L | 0 | N |
| 0.7776 | Temporal Sup L | 0 | TL |
| 0.7641 | Rolandic Oper L | 0 | C |
| 0.7403 | Frontal Inf Tri R | 0 | FL |
| 0.7348 | Caudate L | 0 | N |
| 0.7332 | SupraMarginal R | 0 | PL |
| 0.6595 | Frontal Inf Oper R | 0 | FL |
| 0.6582 | Lingual L | 0 | OM |
| 0.6557 | Frontal Inf Orb L | 0 | FO |
| 0.6467 | Olfactory L | 0 | FO |
| 0.6428 | Postcentral R | 0 | C |
| 0.6166 | Temporal Pole Sup R | 0 | L |
| 0.6137 | Calcarine R | 0 | OM |
| 0.5989 | Rectus R | 0 | FO |
| 0.5952 | Paracentral Lobule R | 0 | FM |
| 0.5888 | Postcentral L | 0 | C |
| 0.5700 | Paracentral Lobule L | 0 | FM |
| 0.5631 | Pallidum L | 0 | N |
| 0.5540 | Angular L | 0 | PL |
| 0.5432 | Amygdala R | 0 | N |
| 0.5311 | Calcarine L | 0 | OM |
| 0.5266 | Frontal Sup Orb R | 0 | FO |
| 0.4838 | Temporal Pole Mid L | 0 | L |
| 0.4800 | Frontal Sup Orb L | 0 | FO |
| 0.4798 | Frontal Inf Orb R | 0 | FO |
| 0.4607 | Precentral L | 0 | C |
| 0.4571 | Frontal Med Orb L | 0 | FO |
| 0.4559 | Rectus L | 0 | FO |
| 0.4510 | Heschl L | 0 | TL |
| 0.4439 | Thalamus R | 0 | N |
| 0.4344 | SupraMarginal L | 0 | PL |
| 0.4132 | Temporal Pole Mid R | 0 | L |
| 0.4108 | Olfactory R | 0 | FO |
| 0.4074 | ParaHippocampal R | 0 | L |
| 0.3668 | Precentral R | 0 | C |
| 0.3333 | Cingulum Post L | 0 | L |
| 0.3009 | Cingulum Post R | 0 | L |
| 0.2882 | Amygdala L | 0 | N |
| 0.1893 | Hippocampus R | 0 | L |
| 0.0697 | Heschl R | 0 | TL |

**AD group**

| Mean Betweenness Centrality (NBC) | Region Name | Number of times as hub | Brain  Lobe |
| --- | --- | --- | --- |
| **2.8272** | **Occipital Mid L** | **19** | **OL** |
| **2.5372** | **Occipital Inf L** | **19** | **OL** |
| **2.3577** | **Occipital Mid R** | **19** | **OL** |
| **2.317** | **Frontal Sup R** | **19** | **FL** |
| **1.9579** | **Occipital Sup L** | **17** | **OL** |
| **1.888** | **Parietal Sup L** | **16** | **PL** |
| **1.8629** | **Occipital Inf R** | **18** | **OL** |
| **1.7956** | **Precuneus R** | **16** | **PM** |
| **1.7064** | **Occipital Sup R** | **17** | **OL** |
| **1.6788** | **Postcentral R** | **15** | **C** |
| **1.5841** | **Fusiform L** | **11** | **OM** |
| **1.5601** | **Temporal Mid L** | **13** | **TL** |
| **1.531** | **Cuneus R** | **13** | **OM** |
| 1.4949 | Cingulum Mid R | 10 | L |
| 1.489 | Cuneus L | 5 | OM |
| 1.4535 | Frontal Sup L | 6 | FL |
| 1.4216 | SupraMarginal R | 8 | PL |
| 1.3665 | Parietal Sup R | 5 | PL |
| 1.3605 | SupraMarginal L | 7 | PL |
| 1.3372 | Lingual L | 0 | OM |
| 1.296 | Temporal Inf R | 3 | TL |
| 1.281 | Olfactory L | 0 | FO |
| 1.2674 | Insula L | 0 | I |
| 1.2606 | Cingulum Ant L | 5 | L |
| 1.2582 | Precentral R | 0 | C |
| 1.2477 | Precuneus L | 7 | PM |
| 1.2356 | Frontal Sup Medial L | 0 | FM |
| 1.2221 | Frontal Inf Oper L | 0 | FL |
| 1.2079 | Cingulum Mid L | 2 | L |
| 1.1823 | Putamen L | 2 | N |
| 1.1613 | Fusiform R | 6 | OM |
| 1.1537 | Frontal Inf Orb R | 3 | FO |
| 1.1461 | Parietal Inf L | 0 | PL |
| 1.1224 | Temporal Mid R | 0 | TL |
| 1.0983 | Precentral L | 0 | C |
| 1.0296 | Temporal Pole Mid L | 3 | L |
| 1.0222 | Putamen R | 0 | N |
| 1.0189 | Frontal Sup Orb R | 0 | FO |
| 1.017 | Temporal Pole Sup L | 0 | L |
| 1.0156 | Postcentral L | 0 | C |
| 1.0066 | Frontal Inf Orb L | 0 | FO |
| 0.98636 | Rolandic Oper L | 0 | C |
| 0.9727 | Frontal Mid R | 0 | FL |
| 0.96648 | Rolandic Oper R | 0 | C |
| 0.9638 | Rectus R | 0 | FO |
| 0.95303 | Cingulum Ant R | 0 | L |
| 0.92873 | Frontal Sup Orb L | 0 | FO |
| 0.91232 | Temporal Sup L | 0 | TL |
| 0.86219 | ParaHippocampal R | 0 | L |
| 0.85968 | Pallidum L | 0 | N |
| 0.83212 | Olfactory R | 0 | FO |
| 0.82512 | Frontal Med Orb L | 0 | FO |
| 0.80719 | Temporal Sup R | 0 | TL |
| 0.79327 | Frontal Mid Orb R | 0 | FO |
| 0.79278 | Angular L | 0 | PL |
| 0.78207 | Temporal Pole Mid R | 0 | L |
| 0.76256 | Pallidum R | 0 | N |
| 0.72569 | Thalamus R | 0 | N |
| 0.72157 | Frontal Mid Orb L | 0 | FO |
| 0.72042 | Supp Motor Area L | 1 | FM |
| 0.72008 | Rectus L | 0 | FO |
| 0.71859 | Amygdala R | 0 | N |
| 0.71214 | Insula R | 0 | I |
| 0.65695 | Hippocampus L | 0 | L |
| 0.6521 | Frontal Mid L | 0 | FL |
| 0.64958 | Caudate L | 0 | N |
| 0.63954 | Frontal Med Orb R | 0 | FO |
| 0.6154 | Frontal Inf Tri L | 0 | FL |
| 0.61175 | Frontal Inf Tri R | 0 | FL |
| 0.61123 | Calcarine L | 0 | OM |
| 0.59831 | Caudate R | 0 | N |
| 0.57378 | Frontal Sup Medial R | 0 | FM |
| 0.53334 | Parietal Inf R | 0 | PL |
| 0.53148 | Angular R | 0 | PL |
| 0.5244 | Frontal Inf Oper R | 0 | FL |
| 0.51847 | Temporal Pole Sup R | 0 | L |
| 0.49317 | Lingual R | 0 | OM |
| 0.48984 | Temporal Inf L | 0 | TL |
| 0.43079 | Amygdala L | 0 | N |
| 0.42301 | Heschl R | 0 | TL |
| 0.39481 | Hippocampus R | 0 | L |
| 0.38108 | Supp Motor Area R | 0 | FM |
| 0.36997 | Calcarine R | 0 | OM |
| 0.25201 | ParaHippocampal L | 0 | L |
| 0.23463 | Paracentral Lobule L | 0 | FM |
| 0.19086 | Paracentral Lobule R | 0 | FM |
| 0.17298 | Cingulum Post R | 0 | L |
| 0.17001 | Heschl L | 0 | TL |
| 0.094456 | Cingulum Post L | 0 | L |
| 0.089551 | Thalamus L | 0 | N |
