# Supplementary material for: Glucose Metabolism during Resting State Reveals Abnormal Brain Networks Organization in the Alzheimer’s Disease and Mild Cognitive Impairment
Source: PLoS One. 2013 Jul 23;8(7):e68860. doi: 10.1371/journal.pone.0068860 (PMC3720883; doi:10.1371/journal.pone.0068860)
Supplement: Table S6 — List of regions with disrupted glucose metabolism in MCI and AD involved in abnormal CMRgl covariations as compared with NC. (DOC) [file pone.0068860.s009.doc]

**Supporting Information Table S6**

List of regions with disrupted glucose metabolism in MCI and AD involved in abnormal CMRgl covariations as compared with NC. The significant CMRgl covariations (correlation coefficients) are represented in bold. The pairs of structures with glucose hypometabolism and significant CMRgl covariations differences between groups are represented in bold.

**NC vs. MCI**

| Structure with hypometabolism | Brain regions with which the structure is connected | Z value | NC _(Correlation Coefficient)_ | MCI _(Correlation Coefficient)_ |
| --- | --- | --- | --- | --- |
| **ITG.L** | Postcentral R (PoCG.R) | 4.0997 | 0.002 | **-0.626** |

**NC vs.** AD

| Structure with hypometabolism | Brain regions with which the structure is connected | Z value | NC _(Correlation Coefficient)_ | AD _(Correlation Coefficient)_ |
| --- | --- | --- | --- | --- |
| **PCG.R** | Rolandic Oper R (ROL.R) | -3.120 | **-0.468** | 0.037 |
| **IPL.L** | **Occipital Inf L (IOG.L)**  **Occipital Mid L (MOG.L)**  **Fusiform L (FFG.L)**  **Occipital Sup L (SOG.L)**  Parietal Sup L (SPG.L)  **Cuneus L (CUN.L)**  **Temporal Mid L (MTG.L)**  Occipital Inf R (IOG.R) | -4.129  -4.067  -3.748  -3.679  -3.303  -3.299  -3.178  -2.950 | -0.104  0.068  -0.064  0.081  **0.487**  0.062  0.082  **-0.343** | **0.549 0.652**  **0.529**  **0.619**  **0.804**  **0.564**  **0.563**  0.156 |
| **IPL.R** | **Temporal Mid R (MTG.R)** | -3.104 | 0.093 | **0.562** |
| **ANG.R** | Amygdala R (AMYG.L) | -3.284 | **-0.557** | -0.055 |
| **ANG.L** | Frontal Sup L (SFGdor.R)  **Precuneus L (PCUN.L)** | -3.908  -3.072 | **-0.405**  0.156 | **0.248**  **0.600** |
| **PCUN.L** | **Temporal Mid L** **(MTG.L)**  **Fusiform L** **(FFG.L)**  **Occipital Inf L (IOG.L)**  **Occipital Mid L (MOG.L)**  **Angular L (ANG.L)**  Cingulum Mid R (DCG.R) | -3.983  -3.548  -3.388  -3.195  -3.072  3.366 | **-0.279**  -0.200  -0.191  0.021  0.156  **0.599** | **0.388**  **0.394**  **0.378**  **0.523**  **0.600**  0.103 |
| **MTG.R** | Parietal Sup R (SPG.R)  **Parietal Inf R (IPL.R)**  Parietal Sup L (SPG.L)  Frontal Inf Orb L (ORBinf.R) | -3.339  -3.104  -2.881  3.124 | -0.049  0.093  **-0.342**  0.045 | **0.489**  **0.562**  0.146  **-0.463** |
| **MTG.L** | Parietal Sup L (SPG.L)  Parietal Sup R (SPG.R)  **Precuneus L (PCUN.L)**  Precentral L (PreCG.L)  Paracentral Lobule L (PCL.L)  Frontal Sup L (SFGdor.L)  Paracentral Lobule R (PCL.R)  Postcentral R (PoCG.R)  **Parietal Inf L (IPL.L)**  Precentral R (PreCG.R)  Postcentral L (PoCG.L)  Frontal Inf Orb L (ORBinf.L)  Frontal Inf Orb R (ORBinf.R)  Temporal Pole Sup R (TPOsup.R)  Pallidum R (PAL.R)  Amygdala R (AMYG.R)  Olfactory R (OLF.R) | -5.373  -4.793  -3.983  -3.563  -3.489  -3.452  -3.219  -3.178  -3.171  -2.954  -2.930  3.044  -3.074  3.097  3.214  -3.214  -3.789 | **-0.395**  **-0.634**  **-0.279**  **-0.373**  **-0.633**  **-0.578**  **-0.641**  **-0.671**  0.082  **-0.753**  **-0.307**  **0.492**  0.077  0.197  0.049  0.079  0.057 | **0.478**  0.089  **0.388**  0.227  -0.136  -0.056  -0.196  **-0.245**  **0.563**  **-0.402**  0.196  0.027  **-0.426**  **-0.325**  **-0.455**  **-0.448**  **-0.540** |
| **ITG.L** | Parietal Sup L (SPG.L)  Parietal Sup R (SPG.R)  Frontal Sup L (SFGdor.L)  Precentral L (PreCG.L)  Olfactory R (OLF.R)  Frontal Inf Orb L (ORBinf.R) | -3.972  -3.498  -2.963  -2.907  2.921  3.038 | **-0.331**  **-0.535**  **-0.359**  **-0.313**  0.051  **0.584** | **0.337**  0.014  0.141  0.181  **-0.429**  0.137 |
| **HIP.L** | SupraMarginal L (SMG.L)  Temporal Sup L (STG.L) | 3.028  3.235 | **0.457**  **0.551** | -0.035  0.055 |
| **CUN.L** | Parietal Sup L (SPG.L)  **Parietal Inf L (IPL.L)** | -3.599  -3.299 | 0.119  0.062 | **0.634**  **0.564** |
| **SOG.L** | Parietal Sup L (SPG.L)  **Parietal Inf L (IPL.L)**  **Occipital Mid L (MOG.L)**  **Fusiform L (FFG.L)**  **Occipital Inf L** **(IOG.L)** | -3.817  -3.679  -3.450  -2.942  -3.443 | **0.393**  0.081  **0.778**  0.105  **0.594** | **0.794**  **0.619**  **0.928**  **0.551**  **0.858** |
| **MOG.L** | Parietal Sup L (SPG.L)  **Parietal Inf L (IPL.L)**  **Occipital Sup L (SOG.L)**  Parietal Sup R (SPG.R)  **Precuneus L (PCUN.L)**  **Occipital Inf L** **(IOG.L)**  **Pallidum L (PAL.L)** | -5.289  -4.067  -3.450  -3.282  -3.195  -3.146  -3.438 | 0.106  0.068  **0.777**  -0.025  0.021  **0.825**  0.146 | **0.774**  **0.652**  **0.927**  **0.499**  **0.523**  **0.938**  **-0.425** |
| **IOG.L** | Parietal Sup L (SPG.L)  **Parietal Inf L (IPL.L)**  Parietal Sup R (SPG.R)  **Occipital Sup L** **(SOG.L)**  **Precuneus L (PCUN.L)**  **Occipital Mid L (MOG.L)**  **Pallidum L (PAL.L)** | -5.401  -4.129  -3.867  -3.443  -3.388  -3.146  3.327 | -0.098  -0.104  -0.140  **0.594**  -0.191  **0.825**  0.101 | **0.689**  **0.548**  **0.489**  **0.858**  **0.378**  **0.938**  **-0.447** |
| **FFG.L** | Parietal Sup L (SPG.L)  **Parietal Inf L (IPL.L)**  Parietal Sup R (SPG.R)  **Precuneus L (PCUN.L)**  Postcentral L (PoCG.L)  Precentral L (PreCG.L)  **Occipital Sup L** **(SOG.L)**  Pallidum R (PAL.R)  Temporal Pole Sup R (TPOsup.R)  Frontal Mid Orb L (ORBmid.L)  Frontal Inf Orb L (ORBinf.L)  Frontal Med Orb L (ORBsupmed.L)  Olfactory R (OLF.R)  Rectus L (REC.L)  Frontal Sup Orb L (ORBsup.L)  Frontal Mid Orb R (ORBmid.R)  Frontal Sup Orb R (ORBsup.R)  Frontal Med Orb R (ORBsupmed.R)  Amygdala R (AMYG.R)  Frontal Inf Orb R (ORBinf.R)  Rectus R (REC.R) | -4.883  -3.748  -3.559  -3.548  -3.432  -3.388  -2.942  3.035  3.156  3.366  3.454  3.577  3.628  3.738  3.857  3.880  4.013  4.017  4.023  4.111  4.118 | -0.092  -0.065  **-0.273**  -0.200  **-0.317**  **-0.310**  0.105  0.023  **0.342**  **0.248**  **0.363**  0.193  0.186  **0.341**  **0.314**  0.211  **0.253**  **0.259**  **0.316**  0.235  **0.299** | **0.641**  **0.529**  **0.329**  **0.394**  **0.265**  **0.265**  **0.551**  **-0.468**  -0.193  **-0.323**  -0.219  **-0.405**  **-0.418**  **-0.289**  **-0.335**  **-0.433**  **-0.416**  **-0.411**  **-0.359**  **-0.445**  **-0.389** |
| **PAL.L** | Olfactory L (OLF.L)  Occipital Sup R (SOG.R)  Cuneus R (CUN.R)  Occipital Mid R (MOG.R)  **Occipital Inf L** **(IOG.L)**  **Occipital Mid L (MOG.L)** | -4.081  2.938  3.028  3.039  3.327  3.438 | 0.238  -0.100  -0.113  -0.067  0.101  0.146 | **0.742**  **-0.547**  **-0.567**  **-0.536**  **-0.447**  **-0.425** |

**MCI vs. AD**

There was not any coincidence.
