# Supplementary material for: Glucose Metabolism during Resting State Reveals Abnormal Brain Networks Organization in the Alzheimer’s Disease and Mild Cognitive Impairment
Source: PLoS One. 2013 Jul 23;8(7):e68860. doi: 10.1371/journal.pone.0068860 (PMC3720883; doi:10.1371/journal.pone.0068860)
Supplement: Text S1 — Full list of the statistical differences between CMRgl covariation matrices. (DOC) [file pone.0068860.s010.doc]

**Supporting Information Text S1**

**Statistical differences between CMRgl covariation matrices.** The CMRgl covariation is obtained through the Pearson correlation coefficient.

Legend: **Structure A** <--> **Structure B: Z test: Correlation value Group 1: Correlation value Group 2**

**MCI vs. AD**

**Structure A** <--> **Structure B: Z test: Correlation value MCI: Correlation value AD**

1 -- Frontal_Inf_Oper_L <--> SupraMarginal_R : 4.0978 0.044157 -0.59799

2 -- Frontal_Sup_Medial_L <--> Frontal_Sup_Orb_L : -3.8969 0.35868 0.7908

3 -- Frontal_Sup_Medial_L <--> Frontal_Inf_Orb_L : -4.0538 0.27283 0.7642

4 -- Pallidum_L <--> Olfactory_L : -5.5791 -0.044029 0.74228

**NC vs. MCI**

**Structure A** <--> **Structure B: Z test: Correlation value NC: Correlation value MCI**

1 -- Fusiform_L <--> Frontal_Med_Orb_R : 3.9609 0.25862 -0.41992

2 -- Fusiform_L <--> Frontal_Inf_Orb_R : 3.9012 0.235 -0.43172

3 -- Fusiform_L <--> Frontal_Mid_Orb_L : 3.9007 0.24781 -0.42053

4 -- Fusiform_L <--> Frontal_Mid_Orb_R : 3.8221 0.21116 -0.44053

5 -- Fusiform_L <--> Frontal_Sup_Orb_L : 3.8144 0.31435 -0.34568

6 -- Temporal_Pole_Sup_L <--> Temporal_Sup_L : 3.7917 0.68739 0.15981

7 -- Temporal_Mid_L <--> Temporal_Pole_Sup_L : 3.7881 0.61934 0.042749

8 -- Fusiform_L <--> Frontal_Sup_Orb_R : 3.7807 0.25293 -0.39803

9 -- Fusiform_L <--> Frontal_Inf_Orb_L : 3.709 0.36303 -0.27896

10 -- Frontal_Inf_Tri_L <--> Frontal_Inf_Oper_R : -3.5445 -0.17848 0.42759

11 -- Fusiform_L <--> Parietal_Sup_R : -3.5595 -0.27297 0.34519

12 -- Fusiform_L <--> Cuneus_L : -3.6342 -0.00017466 0.5739

13 -- Fusiform_L <--> Postcentral_R : -3.6642 -0.38419 0.2486

14 -- Temporal_Inf_L <--> Postcentral_R : -4.0997 -0.62647 0.0016081

15 -- Temporal_Mid_L <--> Parietal_Sup_R : -4.3143 -0.63386 0.027929

16 -- Temporal_Mid_L <--> Precentral_R : -4.348 -0.75307 -0.19561

17 -- Temporal_Mid_L <--> Postcentral_R : -4.7555 -0.6709 0.042723

**NC vs. AD**

**Structure A** <--> **Structure B: Z test: Correlation value NC: Correlation value AD**

1 -- Frontal_Sup_Orb_L <--> Fusiform_R : 4.5108 0.088952 -0.60377

2 -- Parietal_Sup_L <--> Frontal_Mid_R : 4.1567 0.20131 -0.47946

3 -- Rectus_L <--> Fusiform_R : 4.1236 0.13512 -0.52602

4 -- Fusiform_L <--> Rectus_R : 4.1183 0.29905 -0.38948

5 -- Fusiform_L <--> Frontal_Inf_Orb_R : 4.1112 0.235 -0.44539

6 -- Postcentral_L <--> Frontal_Mid_R : 4.1038 0.29152 -0.39431

7 -- Parietal_Sup_L <--> Frontal_Sup_R : 4.0299 0.34755 -0.32886

8 -- Fusiform_L <--> Amygdala_R : 4.0232 0.31573 -0.35934

9 -- Fusiform_L <--> Frontal_Med_Orb_R : 4.0172 0.25862 -0.41146

10 -- Fusiform_L <--> Frontal_Sup_Orb_R : 4.0126 0.25293 -0.41584

11 -- Frontal_Sup_L <--> Frontal_Mid_R : 4.0062 0.7306 0.22597

12 -- Temporal_Sup_L <--> Pallidum_R : 3.9063 0.25525 -0.39827

13 -- Paracentral_Lobule_L <--> Parietal_Sup_R : 3.9051 0.65419 0.099857

14 -- Precentral_L <--> Frontal_Mid_R : 3.8801 0.42964 -0.21516

15 -- Fusiform_L <--> Frontal_Mid_Orb_R : 3.88 0.21116 -0.43303

16 -- Fusiform_L <--> Frontal_Sup_Orb_L : 3.8566 0.31435 -0.33511

17 -- Postcentral_L <--> Frontal_Sup_R : 3.839 0.41072 -0.23017

18 -- Temporal_Mid_L <--> Olfactory_R : 3.7899 0.057923 -0.54009

19 -- Fusiform_R <--> Frontal_Sup_Orb_R : 3.7837 0.30377 -0.33418

20 -- Postcentral_L <--> Precentral_R : 3.741 0.66875 0.15354

21 -- Frontal_Inf_Orb_L <--> Fusiform_R : 3.7404 -0.0077777 -0.57929

22 -- Fusiform_L <--> Rectus_L : 3.7378 0.34083 -0.2896

23 -- Frontal_Med_Orb_L <--> Fusiform_R : 3.7274 -0.019134 -0.5853

24 -- Fusiform_L <--> Olfactory_R : 3.6284 0.18661 -0.41796

25 -- Fusiform_R <--> Rectus_R : 3.6191 0.23223 -0.37642

26 -- Precentral_L <--> Frontal_Sup_R : 3.5778 0.55901 0.0061912

27 -- Fusiform_L <--> Frontal_Med_Orb_L : 3.5767 0.19266 -0.40525

28 -- Parietal_Sup_L <--> Paracentral_Lobule_R : 3.5667 0.65364 0.15704

29 -- Fusiform_L <--> Frontal_Inf_Orb_L : 3.4542 0.36303 -0.2196

30 -- Parietal_Sup_R <--> Frontal_Sup_R : 3.4502 0.41139 -0.16412

31 -- Pallidum_L <--> Occipital_Mid_L : 3.4385 0.14625 -0.42483

32 -- Paracentral_Lobule_R <--> Parietal_Sup_R : 3.421 0.69595 0.2558

33 -- Postcentral_R <--> Precentral_R : 3.3851 0.90175 0.71139

34 -- SupraMarginal_L <--> Hippocampus_R : 3.3706 0.18249 -0.38374

35 -- Fusiform_L <--> Frontal_Mid_Orb_L : 3.3662 0.24781 -0.32314

36 -- Precuneus_L <--> Cingulum_Mid_R : 3.3661 0.59871 0.10256

37 -- Cingulum_Ant_L <--> Parietal_Sup_R : 3.3624 -0.10785 -0.60172

38 -- Pallidum_L <--> Occipital_Inf_L : 3.3275 0.10067 -0.44661

39 -- Frontal_Sup_L <--> Parietal_Sup_R : 3.3024 0.18982 -0.36698

40 -- Frontal_Mid_L <--> Thalamus_R : 3.2857 0.29031 -0.26852

41 -- Fusiform_R <--> Frontal_Med_Orb_R : 3.2805 0.12612 -0.41899

42 -- Frontal_Sup_L <--> Frontal_Sup_R : 3.2434 0.81714 0.52367

43 -- Temporal_Sup_L <--> Hippocampus_L : 3.2354 0.55138 0.054937

44 -- Postcentral_R <--> Frontal_Sup_R : 3.2313 0.55711 0.063899

45 -- Temporal_Pole_Sup_L <--> Temporal_Sup_L : 3.2168 0.68739 0.27371

46 -- Temporal_Mid_L <--> Amygdala_R : 3.2145 0.079511 -0.44787

47 -- Paracentral_Lobule_L <--> Parietal_Sup_L : 3.2009 0.60961 0.14785

48 -- Fusiform_L <--> Temporal_Pole_Sup_R : 3.1556 0.34161 -0.19305

49 -- Frontal_Inf_Orb_L <--> Temporal_Mid_R : 3.1244 0.045123 -0.46277

50 -- Temporal_Mid_L <--> Pallidum_R : 3.0966 0.049807 -0.45523

51 -- Frontal_Mid_Orb_L <--> Fusiform_R : 3.09 -0.082937 -0.55328

52 -- Temporal_Mid_L <--> Temporal_Pole_Sup_R : 3.0737 0.19732 -0.32496

53 -- Parietal_Sup_L <--> Cingulum_Ant_R : 3.0517 -0.11512 -0.57093

54 -- Fusiform_R <--> Frontal_Inf_Orb_R : 3.045 0.36957 -0.14319

55 -- Temporal_Mid_L <--> Frontal_Inf_Orb_R : 3.0444 0.076609 -0.4262

56 -- Pallidum_L <--> Occipital_Mid_R : 3.0393 -0.067518 -0.53614

57 -- Temporal_Inf_L <--> Frontal_Inf_Orb_L : 3.038 0.58389 0.13661

58 -- Fusiform_L <--> Pallidum_R : 3.0346 0.023279 -0.4676

59 -- Frontal_Inf_Orb_L <--> Lingual_R : 3.0297 0.039537 -0.45411

60 -- Pallidum_L <--> Cuneus_R : 3.0278 -0.11323 -0.56682

61 -- SupraMarginal_L <--> Hippocampus_L : 3.0276 0.4571 -0.035411

62 -- Temporal_Sup_L <--> Temporal_Pole_Sup_R : 3.005 0.37061 -0.13514

63 -- Frontal_Inf_Orb_L <--> Temporal_Sup_R : 2.962 -0.00022251 -0.47602

64 -- Pallidum_L <--> Occipital_Sup_R : 2.9383 -0.10048 -0.54713

65 -- Parietal_Sup_L <--> Cingulum_Ant_L : 2.938 -0.053881 -0.5134

66 -- Paracentral_Lobule_L <--> Precentral_R : 2.9348 0.76909 0.46623

67 -- Frontal_Sup_L <--> Postcentral_R : 2.9306 0.29662 -0.20342

68 -- Temporal_Mid_L <--> Frontal_Inf_Orb_L : 2.9304 0.49233 0.027042

69 -- Temporal_Inf_L <--> Olfactory_R : 2.9215 0.051016 -0.42965

70 -- Frontal_Sup_Medial_L <--> Frontal_Sup_Medial_R : 2.8718 0.86019 0.65969

71 -- Parietal_Sup_L <--> Temporal_Mid_R : -2.8817 -0.34215 0.14598

72 -- Frontal_Sup_Orb_R <--> Frontal_Sup_R : -2.8822 0.19383 0.60434

73 -- Temporal_Inf_L <--> Precentral_L : -2.9067 -0.31346 0.18151

74 -- Temporal_Pole_Sup_L <--> Frontal_Sup_Medial_L : -2.9113 -0.037434 0.43924

75 -- Rectus_L <--> Frontal_Sup_L : -2.9128 0.15086 0.57903

76 -- Frontal_Inf_Orb_L <--> Frontal_Inf_Oper_L : -2.9319 0.31636 0.68577

77 -- Fusiform_L <--> Occipital_Sup_L : -2.9423 0.10549 0.55116

78 -- Parietal_Inf_L <--> Occipital_Inf_R : -2.9504 -0.34353 0.15619

79 -- Temporal_Mid_L <--> Postcentral_L : -2.9542 -0.30724 0.19616

80 -- Temporal_Inf_L <--> Frontal_Sup_L : -2.9633 -0.35933 0.14077

81 -- Frontal_Inf_Orb_R <--> Frontal_Mid_Orb_R : -2.971 0.72215 0.89193

82 -- Caudate_R <--> Frontal_Inf_Orb_R : -2.9769 0.040393 0.50843

83 -- Rectus_R <--> Frontal_Sup_Medial_R : -3.003 0.312 0.68977

84 -- Temporal_Sup_L <--> Parietal_Sup_L : -3.0124 -0.31219 0.20067

85 -- Cingulum_Ant_L <--> Frontal_Inf_Tri_L : -3.0389 -0.0065359 0.48117

86 -- Frontal_Med_Orb_L <--> Frontal_Inf_Oper_L : -3.0453 0.032917 0.51174

87 -- Occipital_Mid_R <--> Occipital_Sup_R : -3.0536 0.82815 0.93736

88 -- Temporal_Pole_Sup_L <--> Paracentral_Lobule_L : -3.062 -0.56294 -0.10171

89 -- Frontal_Inf_Oper_R <--> Frontal_Mid_R : -3.0706 0.41279 0.75113

90 -- Precuneus_L <--> Angular_L : -3.0723 0.15567 0.60043

91 -- Temporal_Pole_Sup_L <--> Supp_Motor_Area_R : -3.0798 -0.62987 -0.20029

92 -- Parietal_Sup_R <--> Occipital_Inf_R : -3.0853 0.13565 0.58867

93 -- Rectus_L <--> Frontal_Sup_Medial_L : -3.0912 0.38081 0.73577

94 -- Temporal_Mid_R <--> Parietal_Inf_R : -3.1043 0.092678 0.56177

95 -- Supp_Motor_Area_L <--> Frontal_Inf_Orb_L : -3.1193 -0.38449 0.13886

96 -- Cingulum_Post_R <--> Rolandic_Oper_R : -3.1202 -0.46838 0.037245

97 -- Occipital_Inf_L <--> Occipital_Mid_L : -3.1458 0.8246 0.93795

98 -- Frontal_Mid_R <--> Frontal_Sup_Orb_R : -3.1527 0.20696 0.64161

99 -- Parietal_Sup_L <--> Calcarine_L : -3.1705 -0.17629 0.35912

100 -- Temporal_Mid_L <--> Precentral_R : -3.171 -0.75307 -0.40188

101 -- Pallidum_R <--> Putamen_R : -3.1775 0.19536 0.63703

102 -- Temporal_Mid_L <--> Parietal_Inf_L : -3.1783 0.082321 0.56347

103 -- Caudate_R <--> Frontal_Sup_Orb_R : -3.1887 0.01251 0.51516

104 -- Precuneus_L <--> Occipital_Mid_L : -3.1954 0.021562 0.52263

105 -- Temporal_Mid_L <--> Postcentral_R : -3.2184 -0.6709 -0.24488

106 -- Temporal_Mid_L <--> Paracentral_Lobule_R : -3.219 -0.64153 -0.1957

107 -- Frontal_Inf_Tri_R <--> Frontal_Mid_R : -3.2415 0.48523 0.79914

108 -- Frontal_Inf_Orb_L <--> Frontal_Sup_Orb_L : -3.2602 0.643 0.86997

109 -- Occipital_Mid_L <--> Parietal_Sup_R : -3.2822 -0.02541 0.49913

110 -- Frontal_Inf_Tri_L <--> Frontal_Mid_Orb_L : -3.283 0.50141 0.80926

111 -- Angular_R <--> Amygdala_R : -3.2839 -0.55753 -0.05533

112 -- Parietal_Inf_L <--> Cuneus_L : -3.2994 0.062349 0.5642

113 -- Parietal_Inf_L <--> Parietal_Sup_L : -3.3026 0.48735 0.80395

114 -- Thalamus_L <--> Amygdala_L : -3.3338 -0.26397 0.30244

115 -- Temporal_Mid_R <--> Parietal_Sup_R : -3.3397 -0.049326 0.48861

116 -- Frontal_Inf_Oper_L <--> Frontal_Mid_L : -3.342 0.37619 0.7529

117 -- Fusiform_L <--> Precentral_L : -3.3876 -0.31019 0.26476

118 -- Precuneus_L <--> Occipital_Inf_L : -3.3882 -0.19134 0.37854

119 -- Frontal_Sup_Medial_L <--> Frontal_Inf_Tri_L : -3.389 0.11842 0.61142

120 -- Frontal_Mid_Orb_R <--> Frontal_Mid_R : -3.4161 0.27019 0.70341

121 -- Frontal_Inf_Orb_L <--> Frontal_Mid_Orb_L : -3.4275 0.73985 0.91363

122 -- Postcentral_L <--> Fusiform_L : -3.4319 -0.31682 0.26513

123 -- Occipital_Inf_L <--> Occipital_Sup_L : -3.4434 0.59447 0.85815

124 -- Occipital_Mid_L <--> Occipital_Sup_L : -3.4502 0.77721 0.92764

125 -- Temporal_Mid_L <--> Frontal_Sup_L : -3.4522 -0.57782 -0.055855

126 -- SupraMarginal_L <--> Precentral_L : -3.453 -0.11255 0.45451

127 -- Caudate_L <--> Cingulum_Ant_L : -3.4715 0.081715 0.59704

128 -- Frontal_Inf_Tri_R <--> Frontal_Inf_Oper_R : -3.4852 0.60326 0.86359

129 -- Temporal_Mid_L <--> Paracentral_Lobule_L : -3.4896 -0.63302 -0.1358

130 -- Temporal_Pole_Sup_L <--> Supp_Motor_Area_L : -3.4925 -0.52134 0.03211

131 -- Temporal_Inf_L <--> Parietal_Sup_R : -3.4984 -0.53474 0.014564

132 -- Caudate_R <--> Rectus_R : -3.5129 -0.15912 0.42468

133 -- Frontal_Inf_Oper_L <--> Frontal_Sup_L : -3.5269 0.048487 0.58158

134 -- Precuneus_L <--> Fusiform_L : -3.5484 -0.20036 0.39437

135 -- Frontal_Inf_Tri_L <--> Frontal_Sup_L : -3.5552 0.058889 0.59166

136 -- Fusiform_L <--> Parietal_Sup_R : -3.5588 -0.27297 0.3291

137 -- Temporal_Mid_L <--> Precentral_L : -3.5627 -0.37274 0.22695

138 -- Parietal_Sup_R <--> Fusiform_R : -3.592 0.11236 0.62946

139 -- Temporal_Sup_L <--> Postcentral_L : -3.5944 -0.13719 0.45426

140 -- Parietal_Sup_L <--> Cuneus_L : -3.599 0.11902 0.63425

141 -- Cingulum_Ant_L <--> Rectus_L : -3.6508 0.37605 0.77528

142 -- Parietal_Inf_L <--> Occipital_Sup_L : -3.6799 0.080845 0.61942

143 -- Frontal_Inf_Orb_R <--> Frontal_Sup_R : -3.7083 -0.00053859 0.56996

144 -- Fusiform_R <--> Occipital_Sup_R : -3.7171 0.13794 0.65749

145 -- Parietal_Inf_L <--> Fusiform_L : -3.7481 -0.06485 0.52991

146 -- SupraMarginal_L <--> Parietal_Sup_L : -3.7554 -0.15897 0.45889

147 -- Parietal_Sup_L <--> Lingual_L : -3.7581 -0.17866 0.44313

148 -- SupraMarginal_L <--> Postcentral_L : -3.7804 -0.064384 0.53429

149 -- Frontal_Inf_Orb_L <--> Frontal_Sup_R : -3.784 -0.41258 0.21893

150 -- Frontal_Sup_Orb_L <--> Frontal_Sup_L : -3.7873 0.18544 0.69078

151 -- Frontal_Inf_Oper_L <--> Frontal_Sup_Orb_L : -3.7999 0.023838 0.59661

152 -- Parietal_Sup_R <--> Occipital_Mid_R : -3.8113 0.30183 0.752

153 -- Parietal_Sup_L <--> Occipital_Sup_L : -3.8167 0.39353 0.79428

154 -- Fusiform_R <--> Occipital_Inf_R : -3.8484 0.30466 0.75614

155 -- Occipital_Inf_L <--> Parietal_Sup_R : -3.8671 -0.14047 0.48871

156 -- Angular_L <--> Frontal_Sup_L : -3.908 -0.40535 0.24761

157 -- Temporal_Pole_Sup_L <--> Frontal_Mid_L : -3.908 -0.26648 0.38833

158 -- Frontal_Inf_Tri_L <--> Frontal_Mid_L : -3.9269 0.48321 0.83769

159 -- Cingulum_Ant_L <--> Frontal_Mid_Orb_L : -3.9593 -0.044087 0.57016

160 -- Temporal_Inf_L <--> Parietal_Sup_L : -3.9716 -0.3308 0.33663

161 -- Temporal_Mid_L <--> Precuneus_L : -3.9827 -0.27906 0.38787

162 -- Parietal_Inf_L <--> Occipital_Mid_L : -4.0666 0.068517 0.65228

163 -- Pallidum_L <--> Olfactory_L : -4.0812 0.23772 0.74228

164 -- Parietal_Inf_L <--> Occipital_Inf_L : -4.1299 -0.10442 0.54896

165 -- Frontal_Mid_L <--> Frontal_Sup_Orb_L : -4.1555 0.2833 0.76882

166 -- Cingulum_Ant_L <--> Frontal_Sup_Orb_L : -4.211 0.1282 0.69871

167 -- Temporal_Pole_Sup_L <--> Frontal_Sup_L : -4.2201 -0.33359 0.37187

168 -- Frontal_Mid_Orb_L <--> Frontal_Mid_L : -4.2716 0.3215 0.79311

169 -- Frontal_Inf_Tri_L <--> Frontal_Sup_Orb_L : -4.3597 0.26875 0.77684

170 -- Fusiform_R <--> Occipital_Mid_R : -4.3808 0.16573 0.7319

171 -- Frontal_Mid_Orb_L <--> Frontal_Sup_L : -4.4358 0.036321 0.67041

172 -- Frontal_Sup_Medial_L <--> Frontal_Sup_Orb_L : -4.484 0.28213 0.7908

173 -- Frontal_Sup_Medial_L <--> Frontal_Mid_Orb_L : -4.5771 0.10967 0.72111

174 -- Cingulum_Ant_L <--> Frontal_Inf_Orb_L : -4.6356 -0.066099 0.63147

175 -- Frontal_Inf_Orb_R <--> Frontal_Mid_R : -4.7154 0.081825 0.71921

176 -- Temporal_Mid_L <--> Parietal_Sup_R : -4.793 -0.63386 0.089464

177 -- Parietal_Sup_L <--> Fusiform_L : -4.8833 -0.092887 0.64119

178 -- Parietal_Sup_L <--> Occipital_Mid_L : -5.2893 0.1057 0.77406

179 -- Temporal_Mid_L <--> Parietal_Sup_L : -5.3731 -0.39522 0.47845

180 -- Parietal_Sup_L <--> Occipital_Inf_L : -5.4014 -0.097854 0.68881

181 -- Frontal_Inf_Orb_L <--> Frontal_Mid_L : -5.4353 0.091129 0.77836

182 -- Frontal_Sup_Medial_L <--> Frontal_Inf_Orb_L : -5.7698 -0.0020165 0.7642

183 -- Frontal_Inf_Orb_L <--> Frontal_Sup_L : -5.971 -0.20141 0.68538
